# Supplementary figures and images for: 1-methylnicotinamide attenuated inflammation and regulated flora in Necrotizing enterocolitis
Source: PLoS One. 2025 Jun 26;20(6):e0324068. doi: 10.1371/journal.pone.0324068 (PMC12200687; doi:10.1371/journal.pone.0324068)

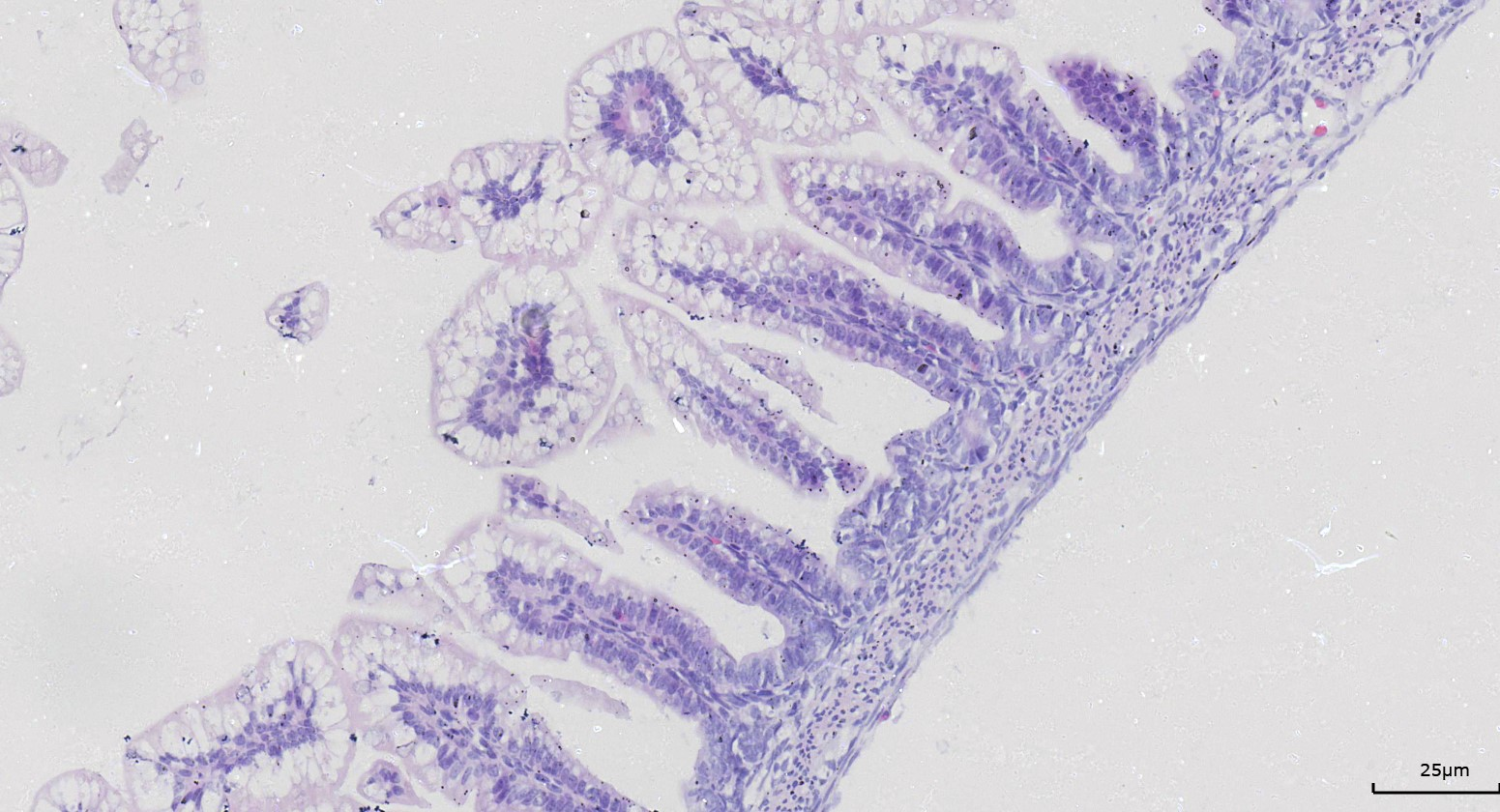

Supplement: S2 File — (ZIP) [file pone.0324068.s002.zip › S2-File/Control/Control 1.tiff]

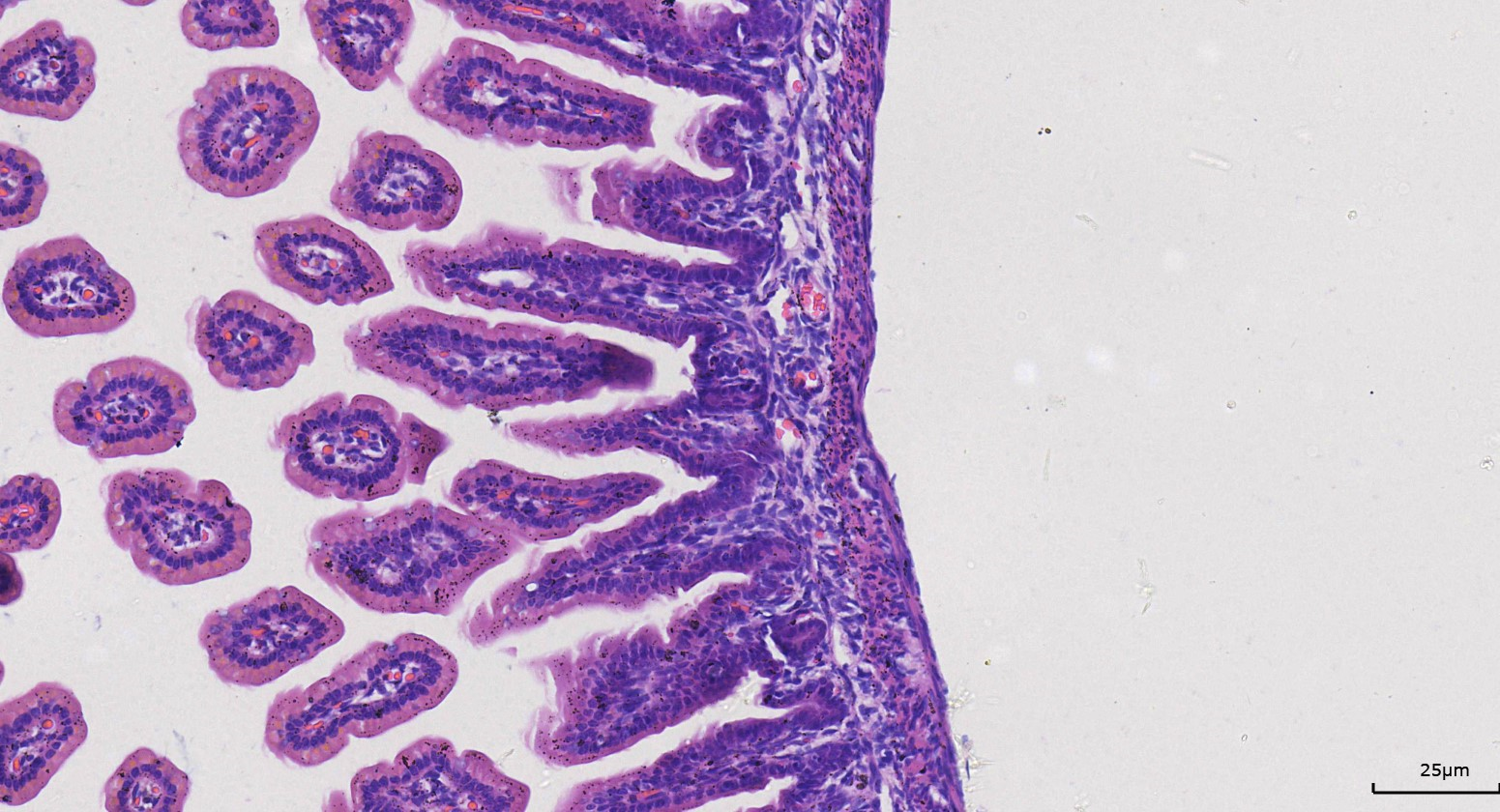

Supplement: S2 File — (ZIP) [file pone.0324068.s002.zip › S2-File/Control/Control 2.tiff]

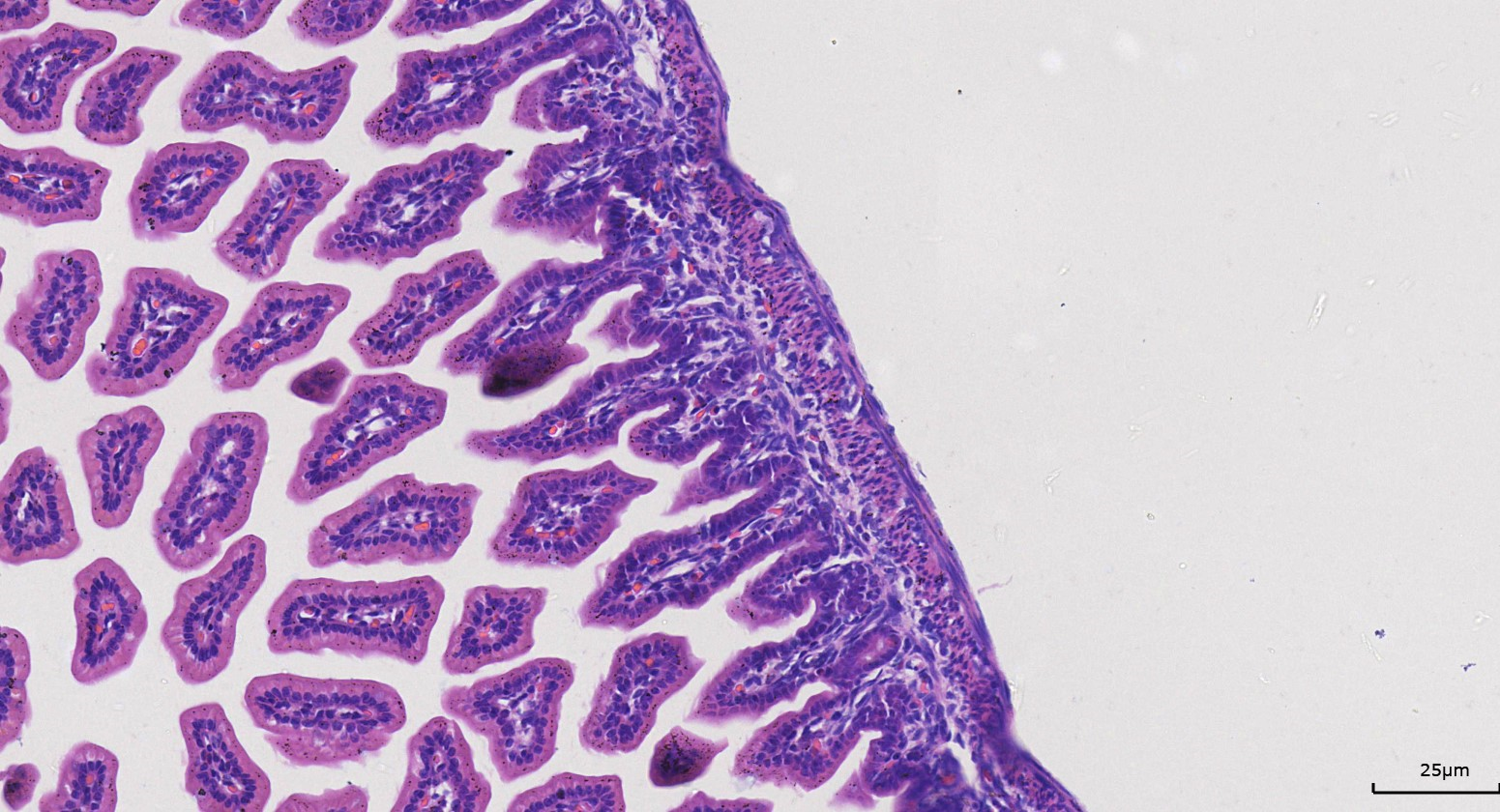

Supplement: S2 File — (ZIP) [file pone.0324068.s002.zip › S2-File/Control/Control 3.tiff]

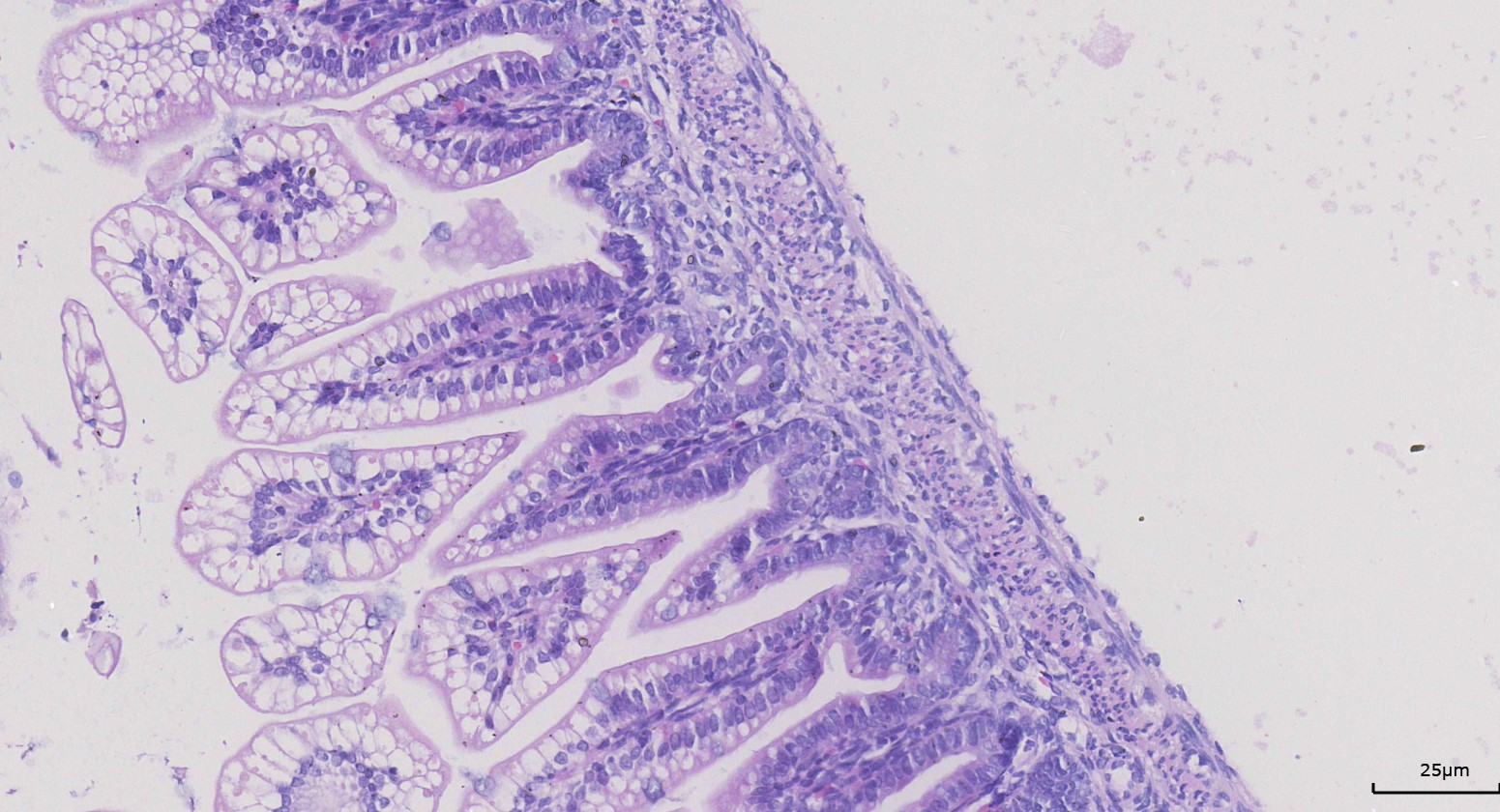

Supplement: S2 File — (ZIP) [file pone.0324068.s002.zip › S2-File/Control/Control 5.tiff]

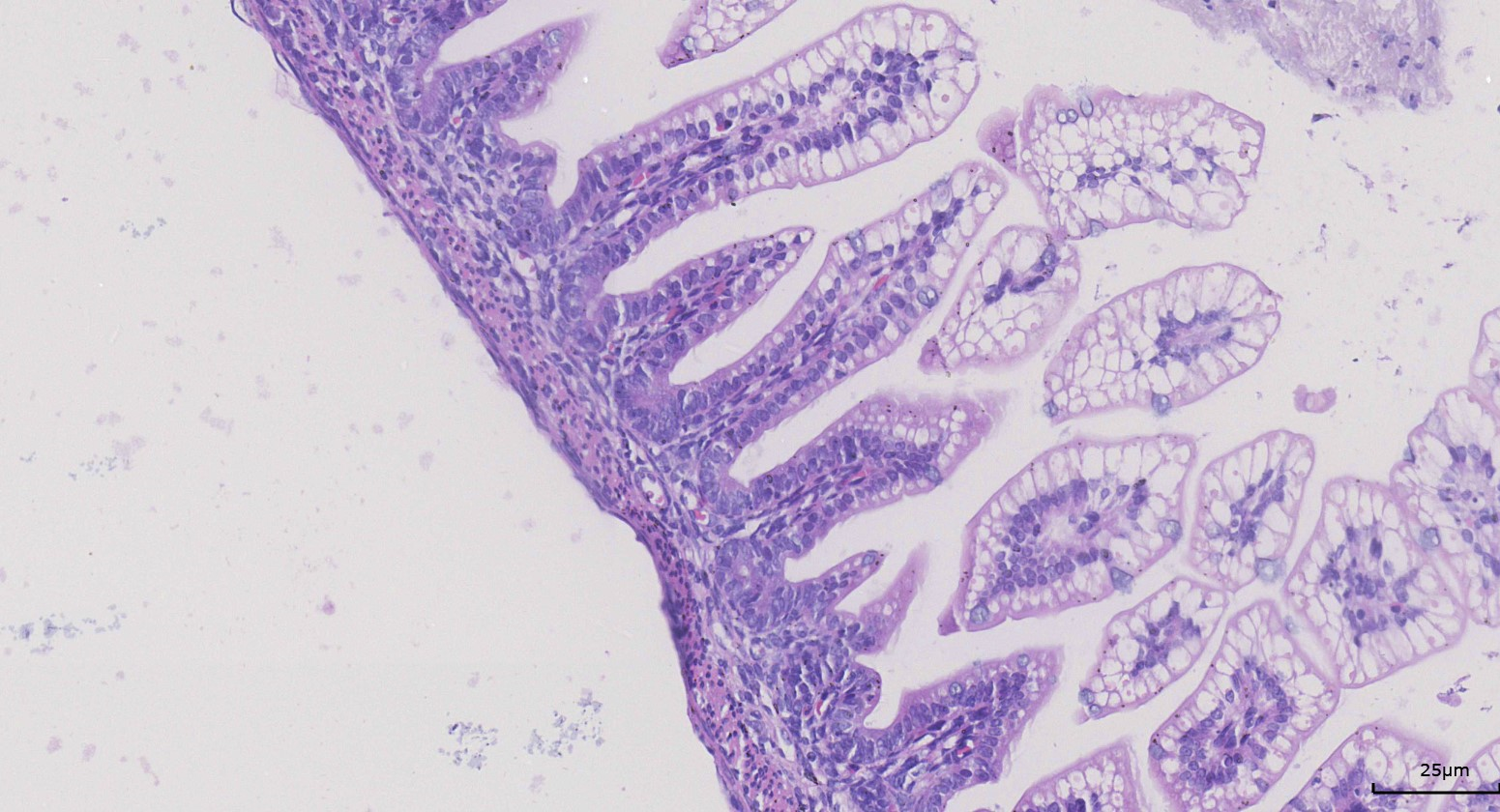

Supplement: S2 File — (ZIP) [file pone.0324068.s002.zip › S2-File/Control/Control 6.tiff]

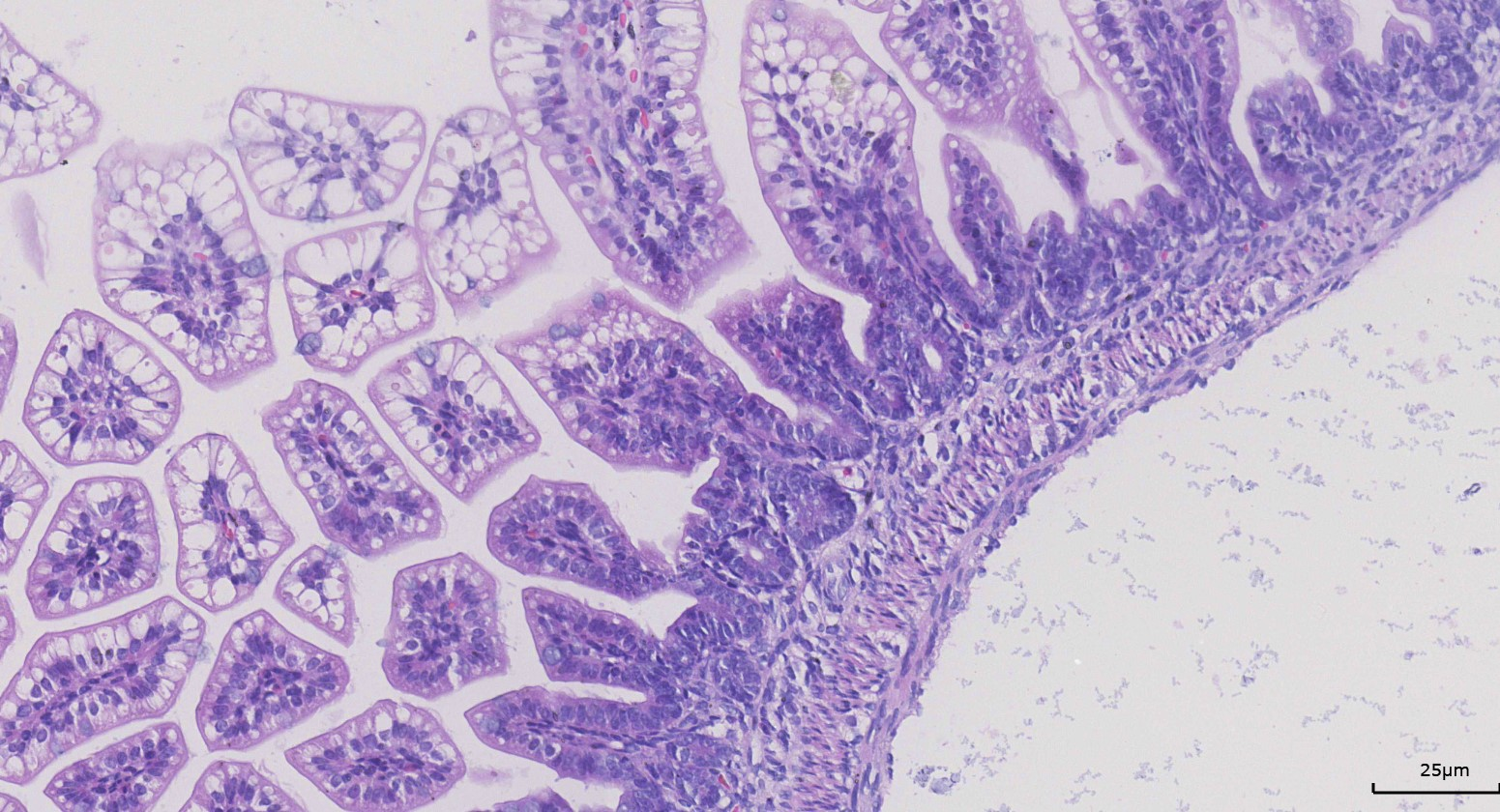

Supplement: S2 File — (ZIP) [file pone.0324068.s002.zip › S2-File/Control/Control 7.tiff]

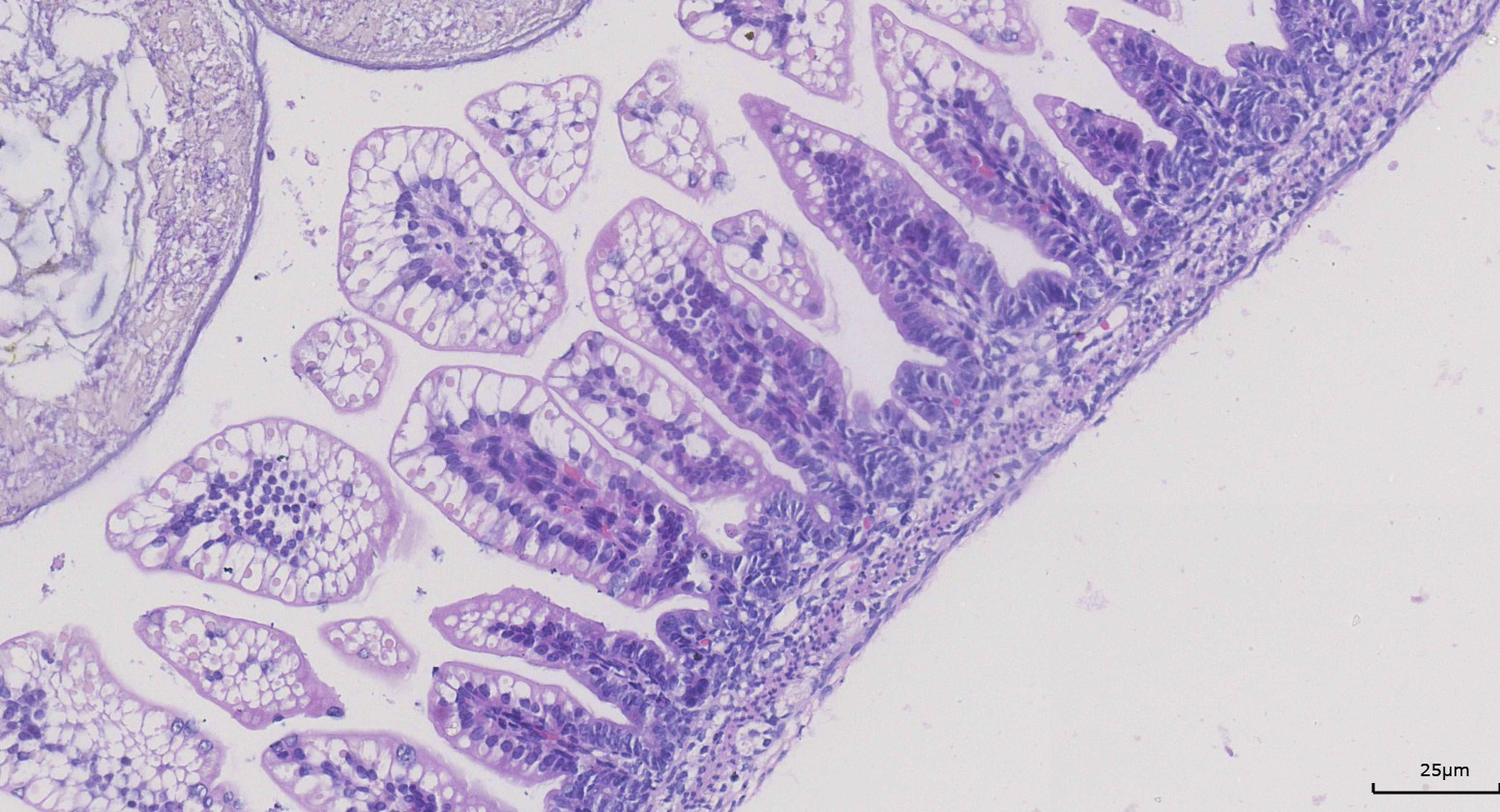

Supplement: S2 File — (ZIP) [file pone.0324068.s002.zip › S2-File/Control/Control 8.tiff]

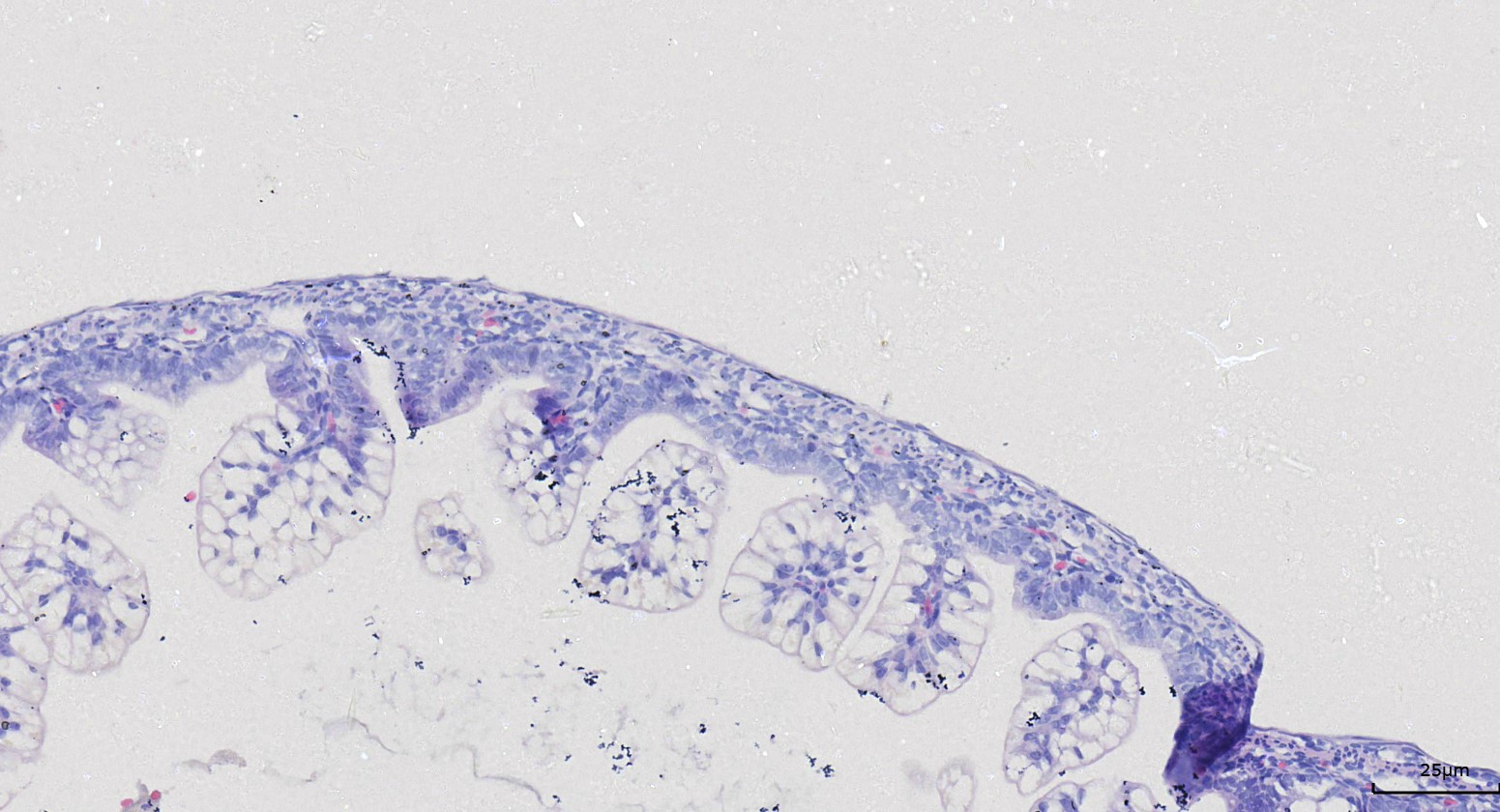

Supplement: S2 File — (ZIP) [file pone.0324068.s002.zip › S2-File/NEC/NEC 1.tiff]

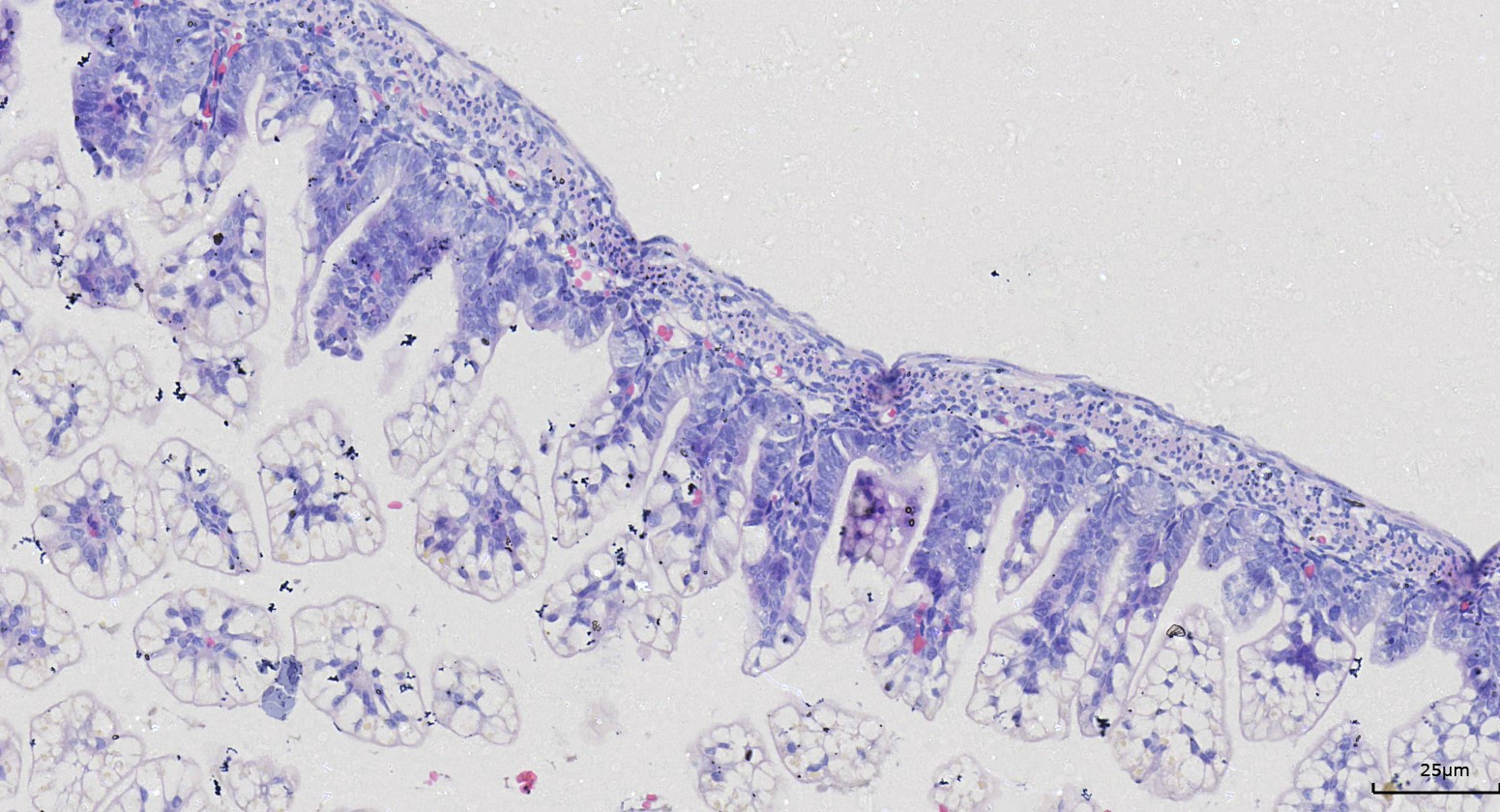

Supplement: S2 File — (ZIP) [file pone.0324068.s002.zip › S2-File/NEC/NEC 2.tiff]

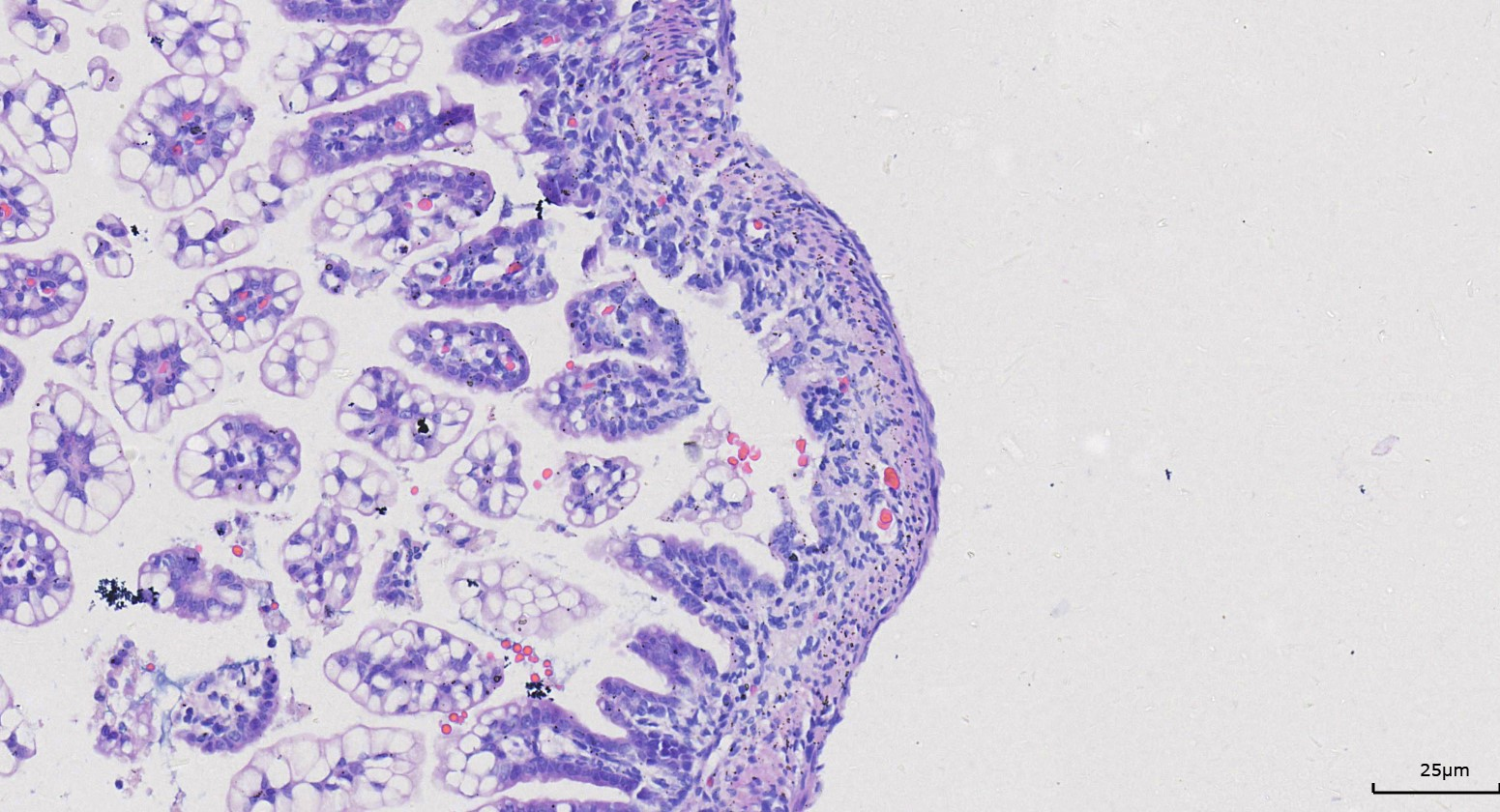

Supplement: S2 File — (ZIP) [file pone.0324068.s002.zip › S2-File/NEC/NEC 3.tiff]

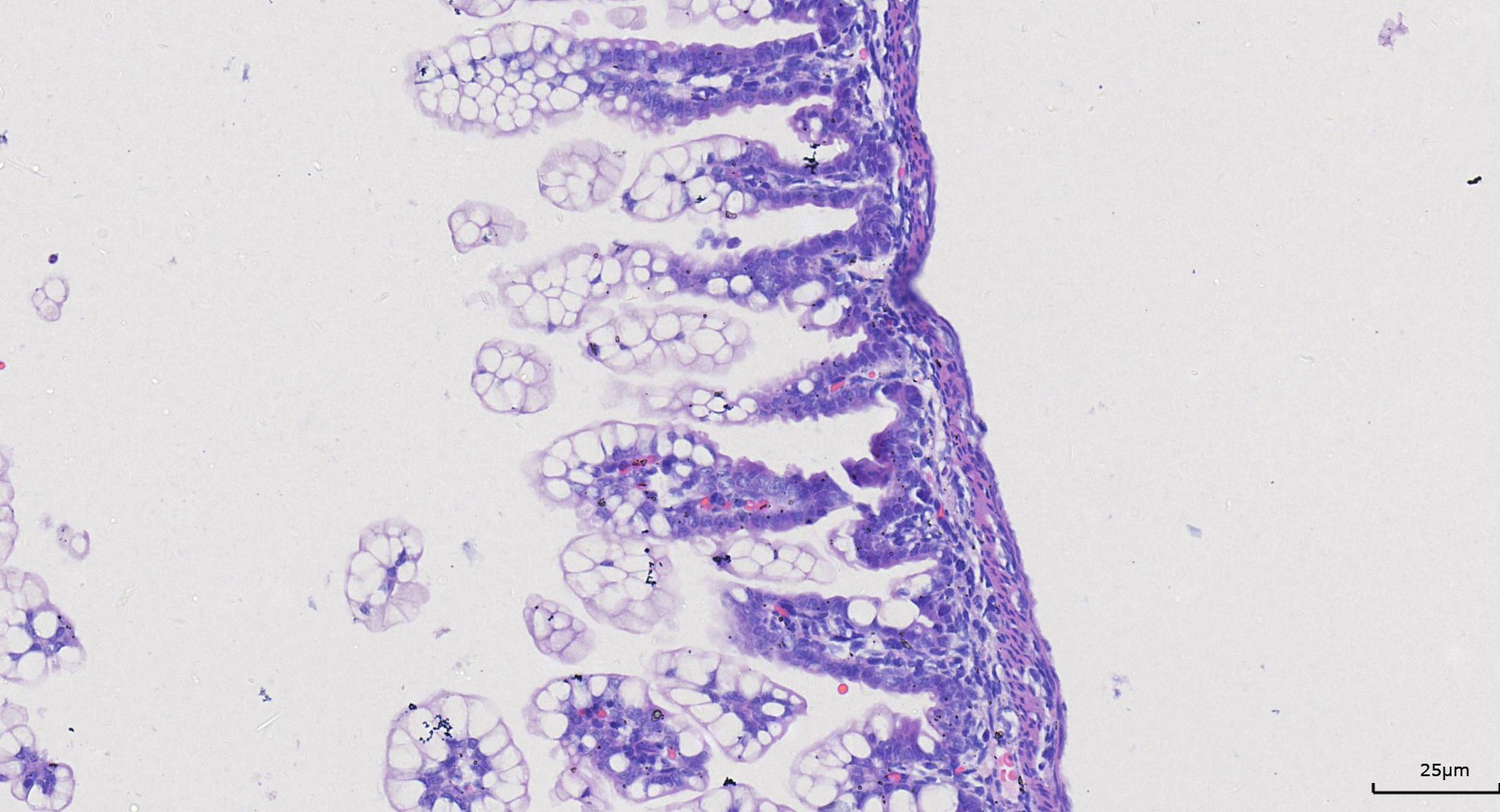

Supplement: S2 File — (ZIP) [file pone.0324068.s002.zip › S2-File/NEC/NEC 4.tiff]

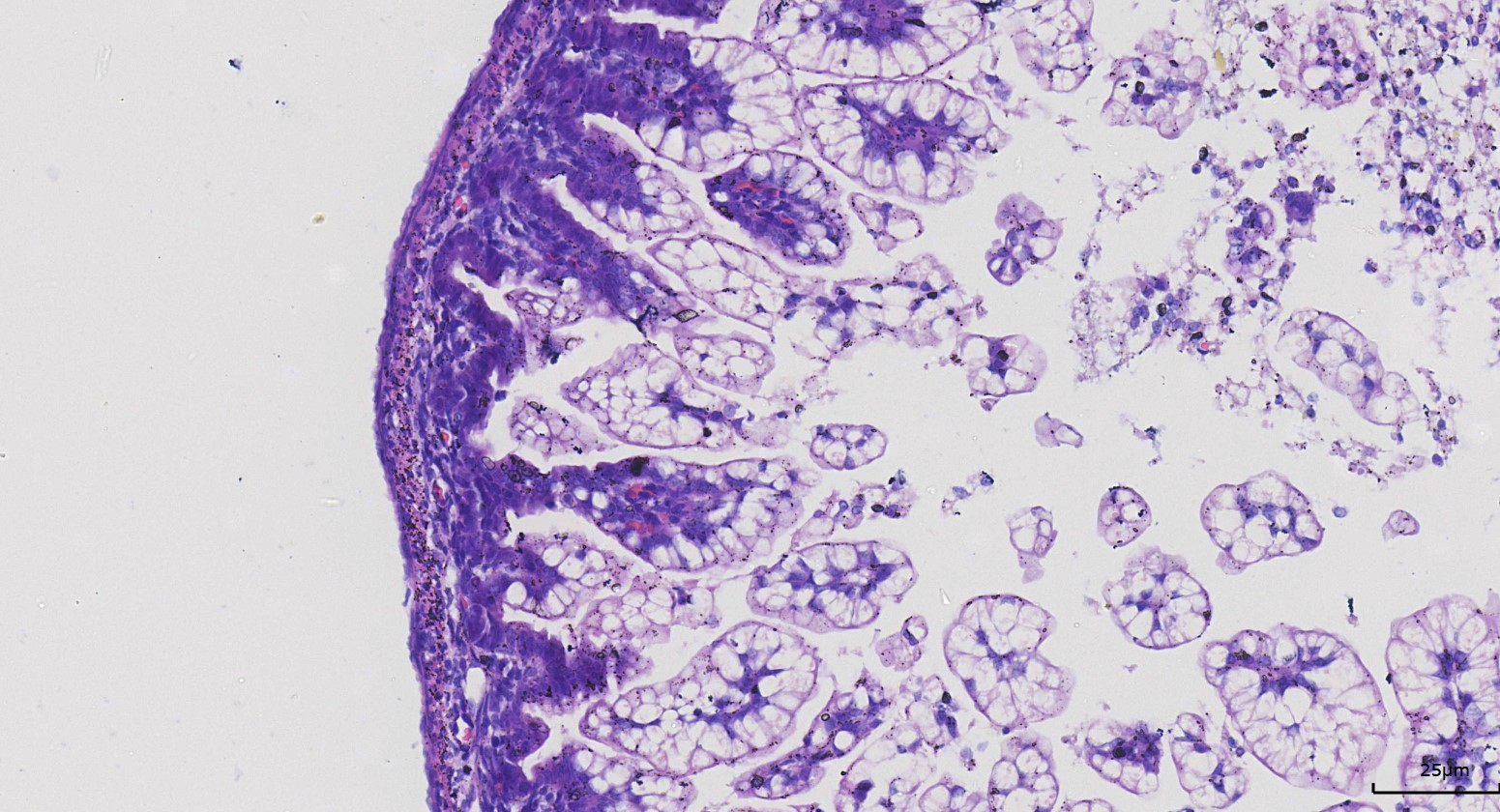

Supplement: S2 File — (ZIP) [file pone.0324068.s002.zip › S2-File/NEC/NEC 5.tiff]

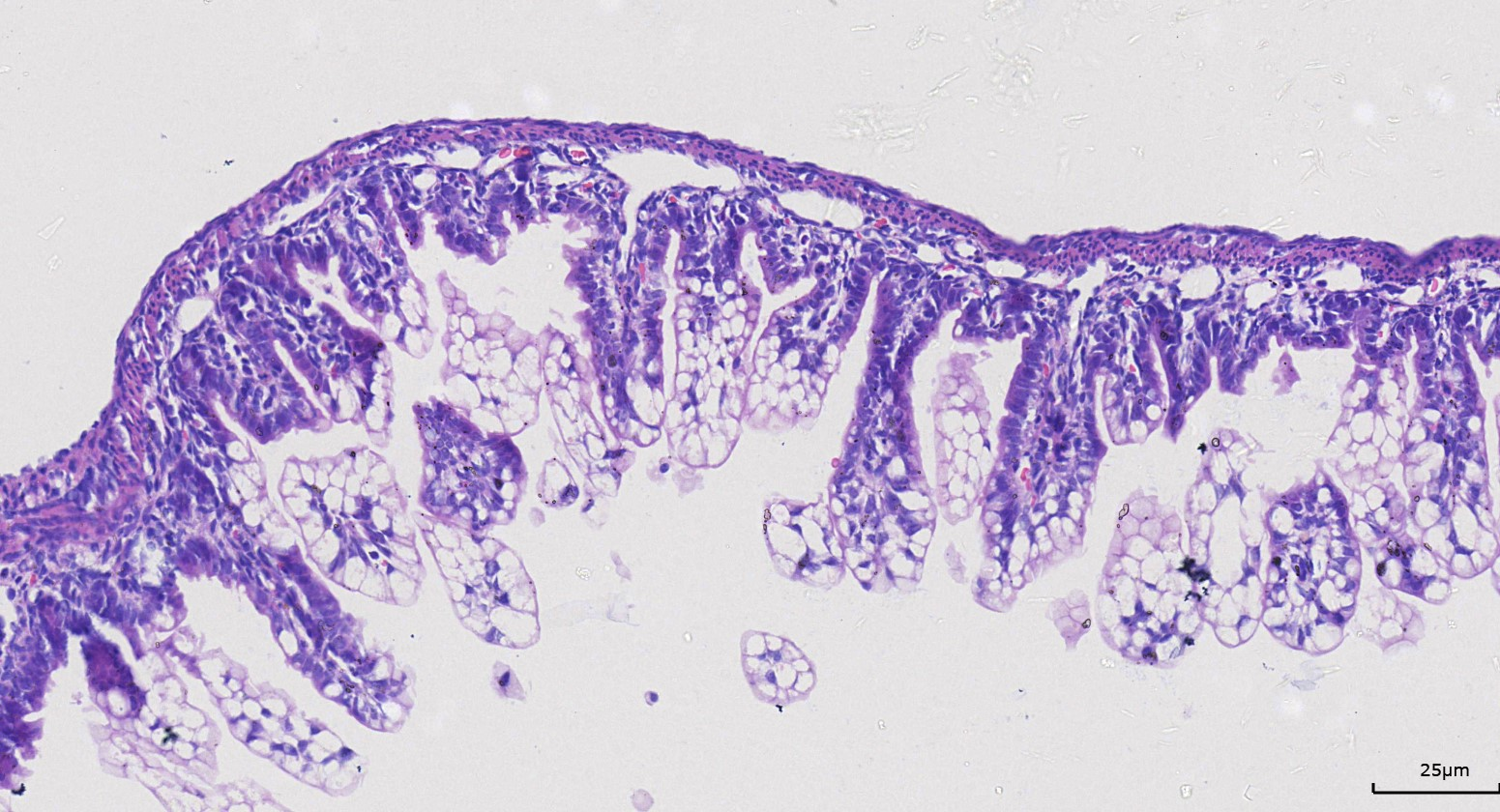

Supplement: S2 File — (ZIP) [file pone.0324068.s002.zip › S2-File/NEC/NEC 6.tiff]

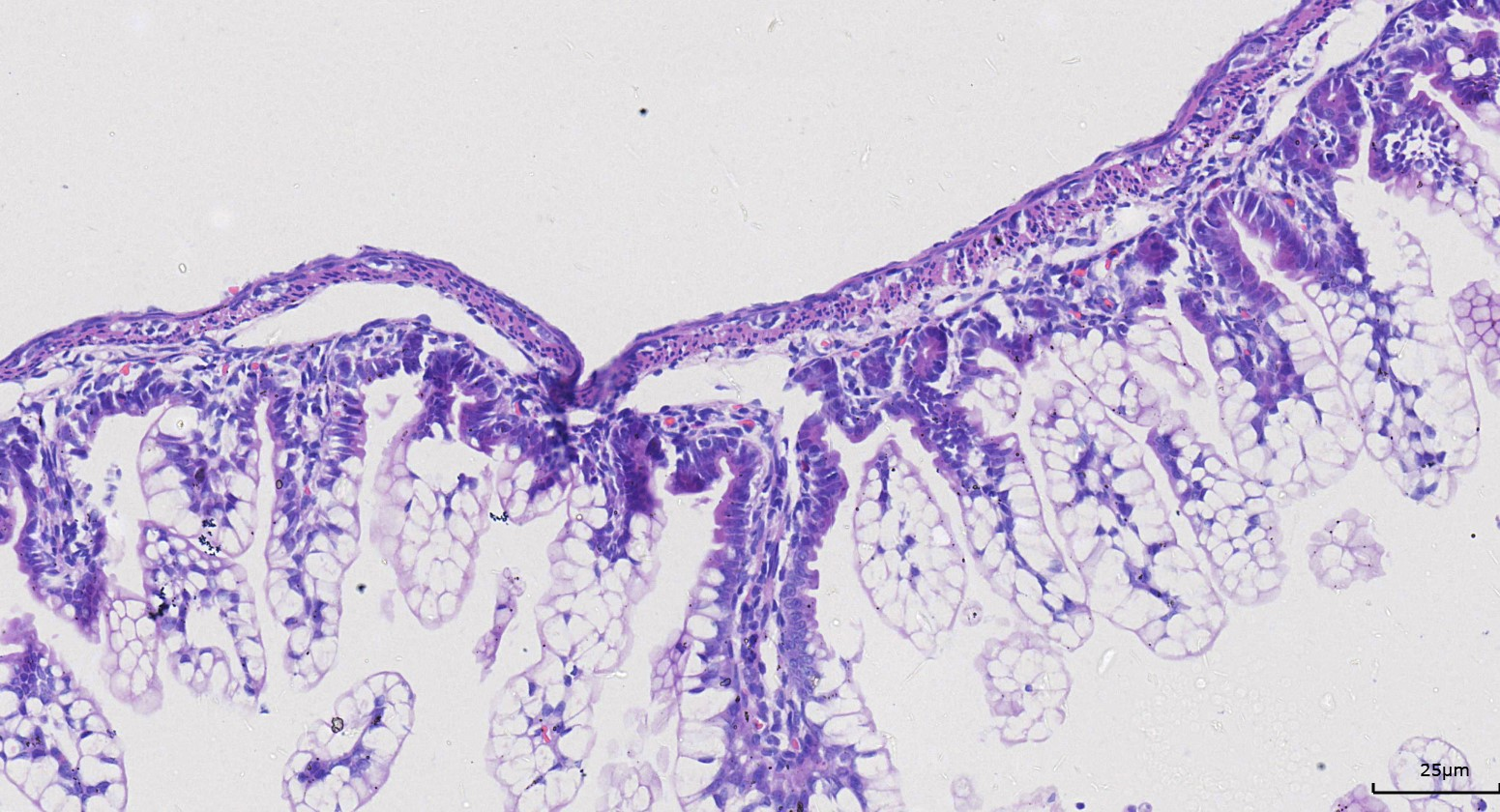

Supplement: S2 File — (ZIP) [file pone.0324068.s002.zip › S2-File/NEC/NEC 7.tiff]

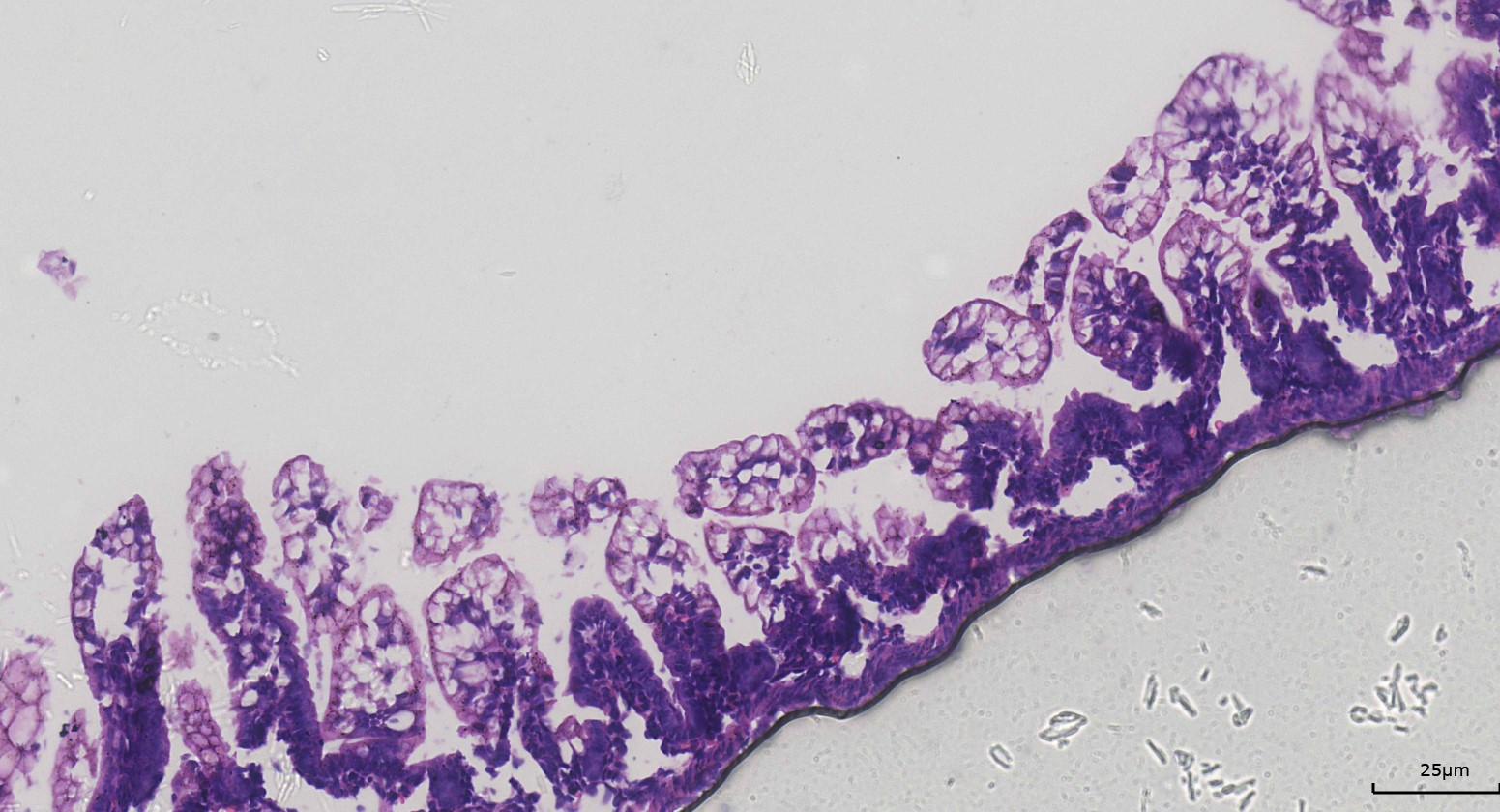

Supplement: S2 File — (ZIP) [file pone.0324068.s002.zip › S2-File/NEC/NEC 8.tiff]

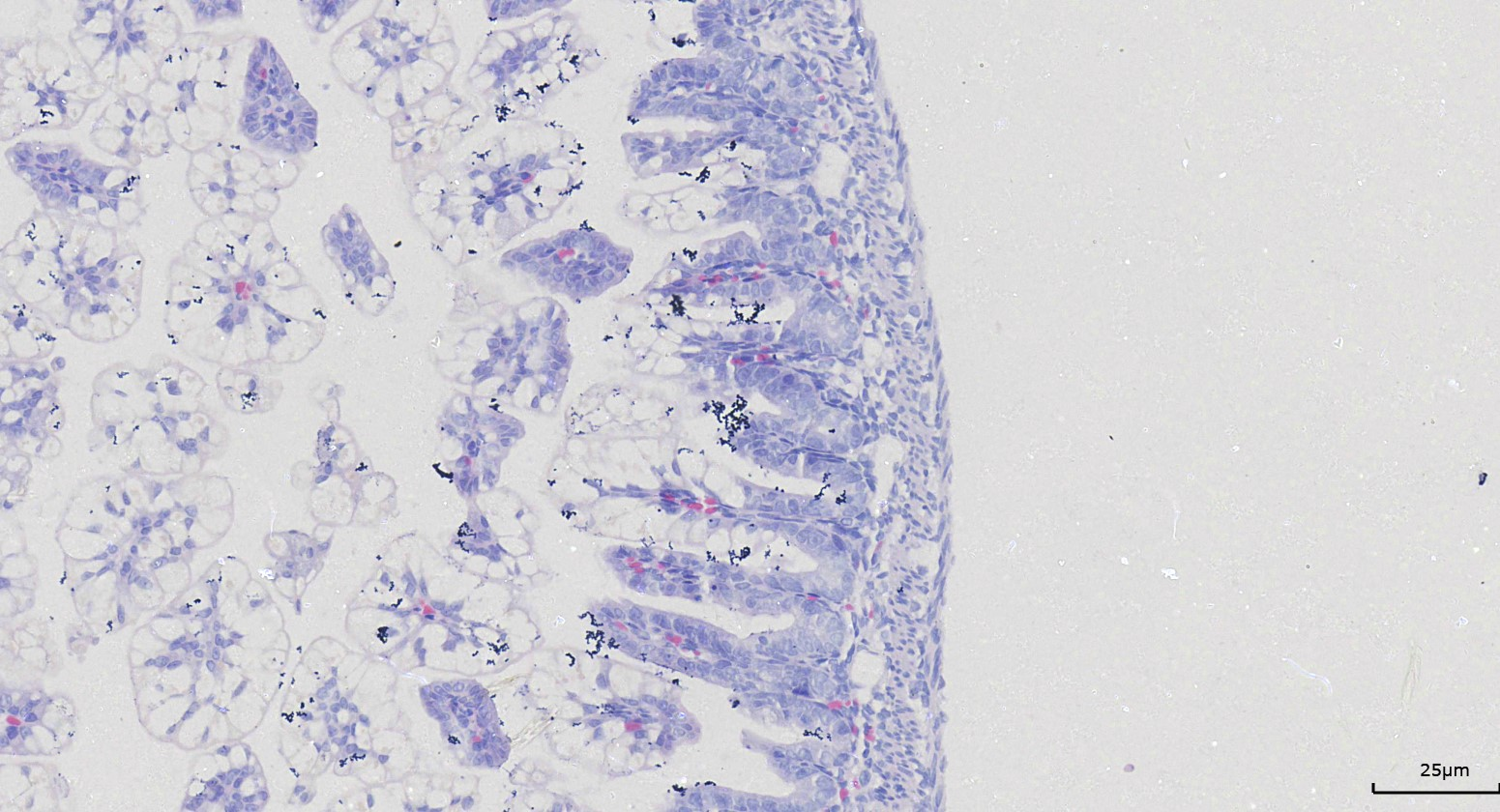

Supplement: S2 File — (ZIP) [file pone.0324068.s002.zip › S2-File/NEC+1-MNA/NEC+1-MNA 1.tiff]

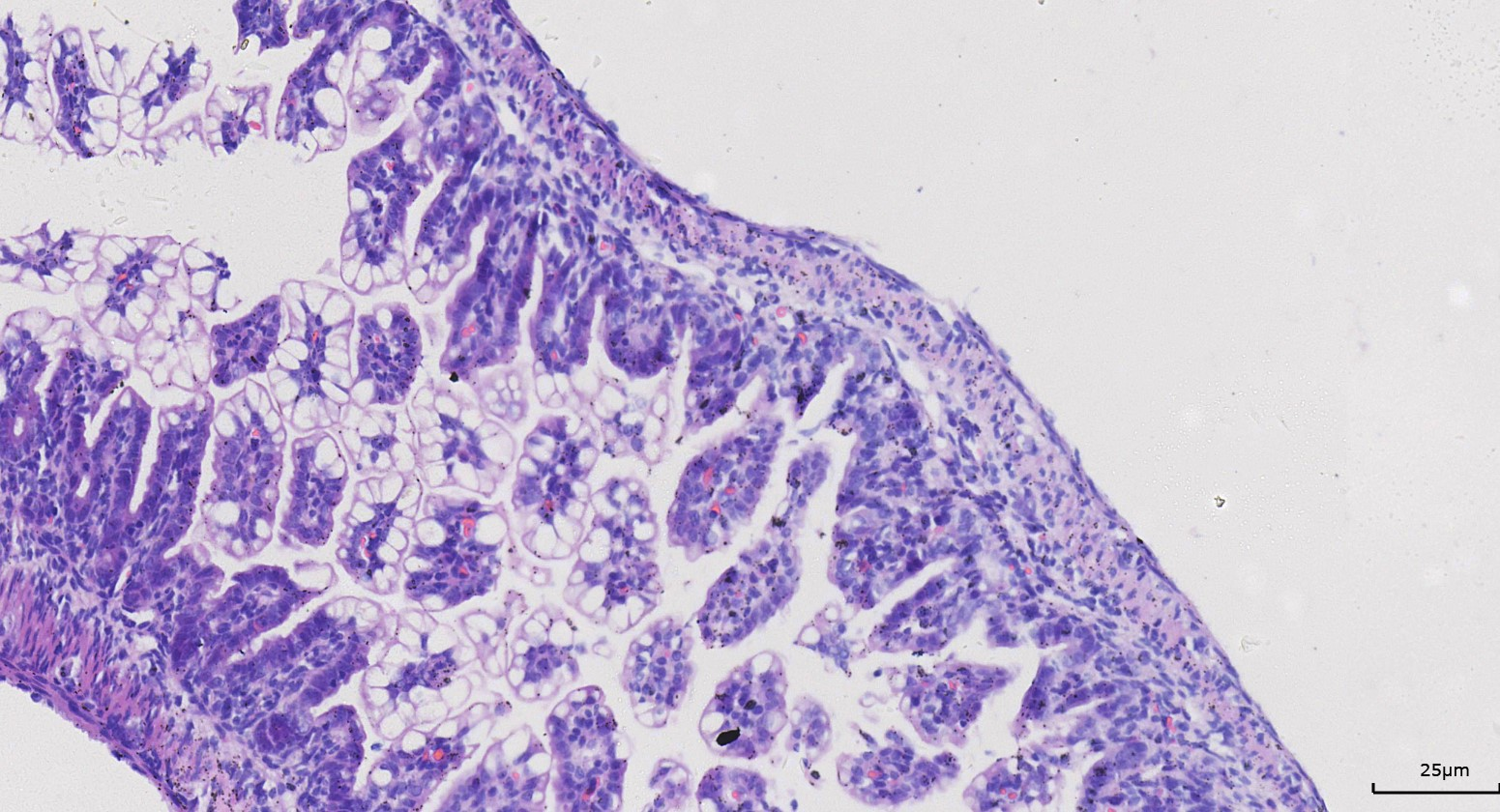

Supplement: S2 File — (ZIP) [file pone.0324068.s002.zip › S2-File/NEC+1-MNA/NEC+1-MNA 2.tiff]

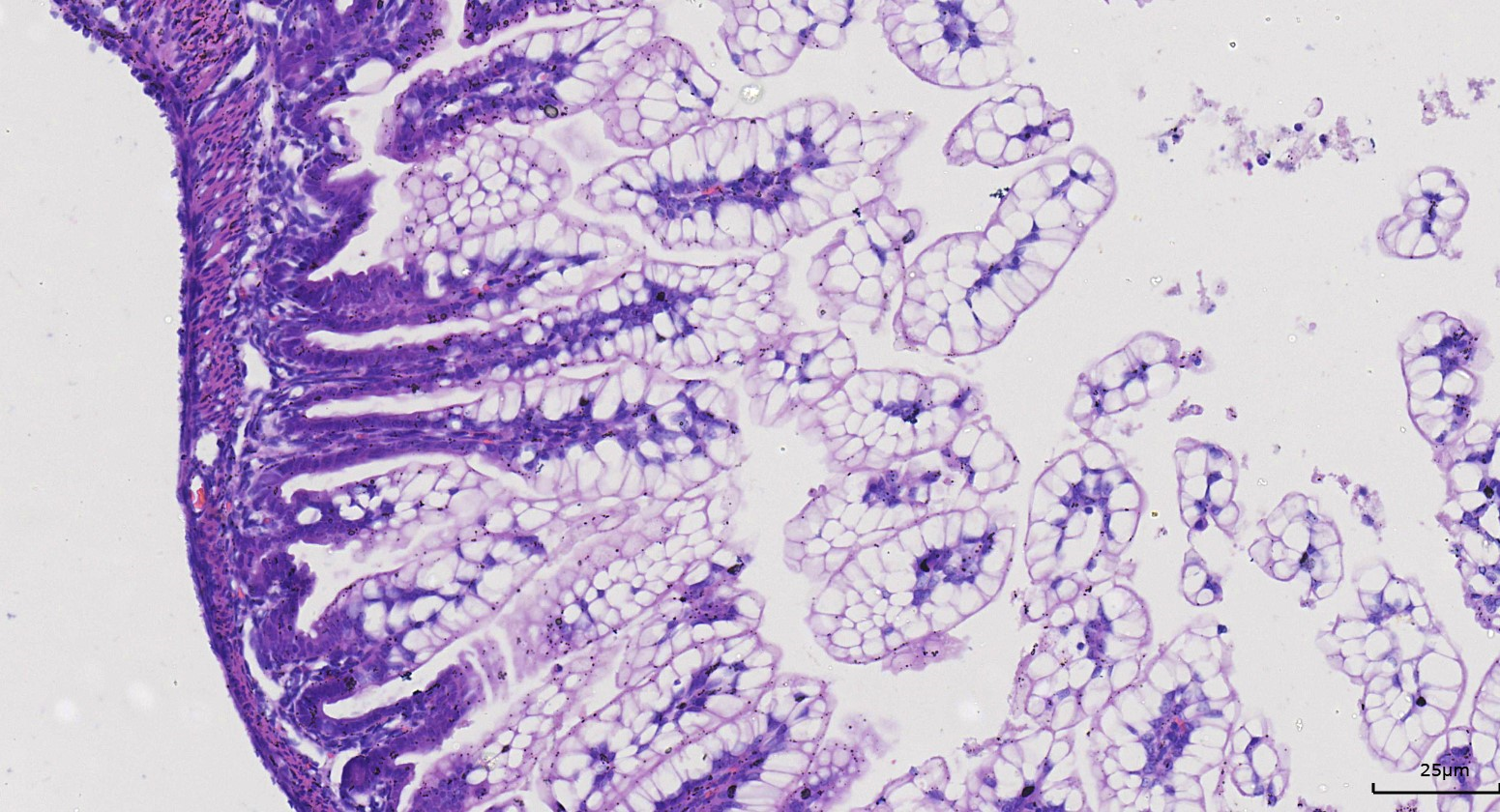

Supplement: S2 File — (ZIP) [file pone.0324068.s002.zip › S2-File/NEC+1-MNA/NEC+1-MNA 3.tiff]

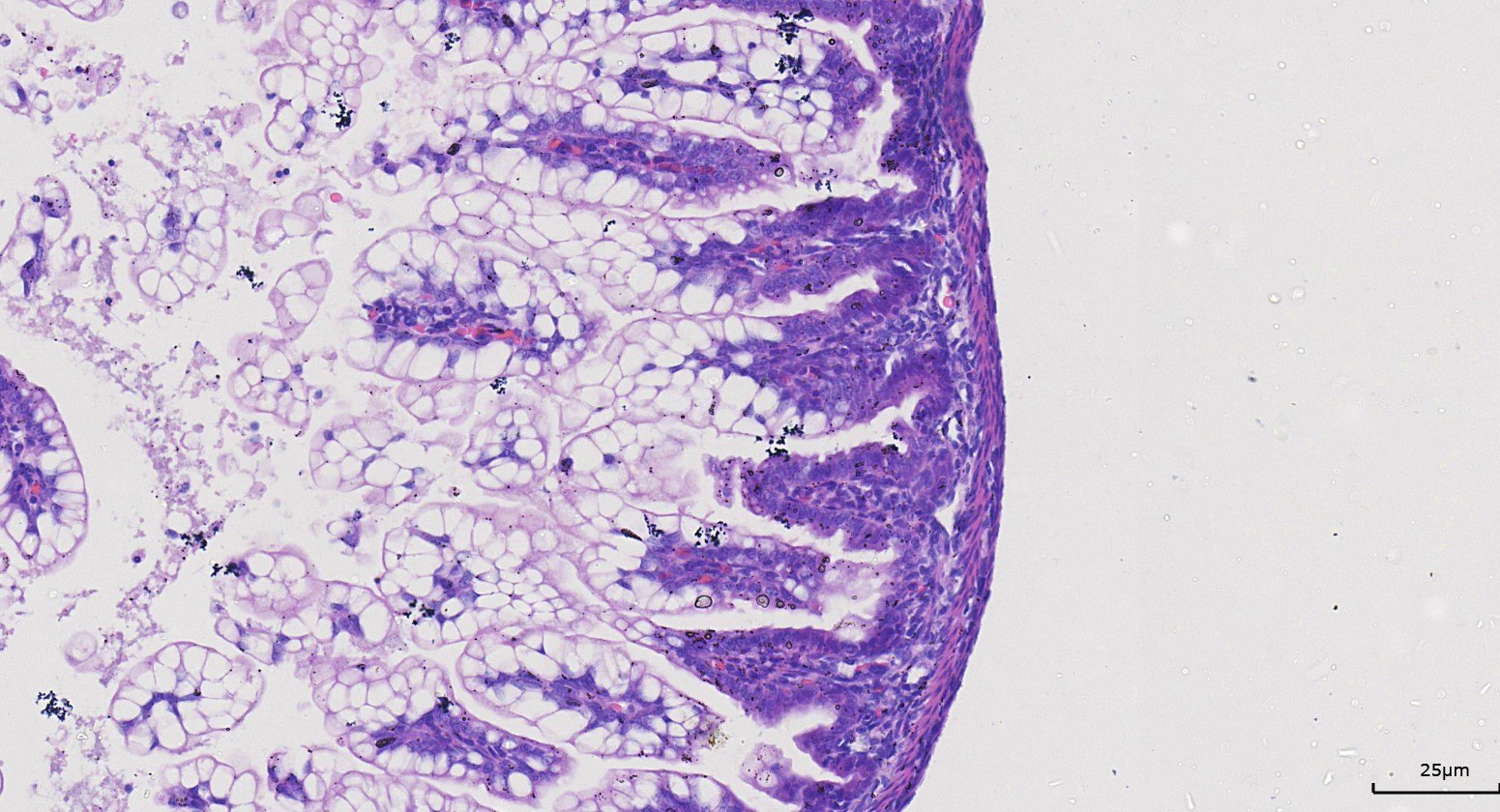

Supplement: S2 File — (ZIP) [file pone.0324068.s002.zip › S2-File/NEC+1-MNA/NEC+1-MNA 4.tiff]

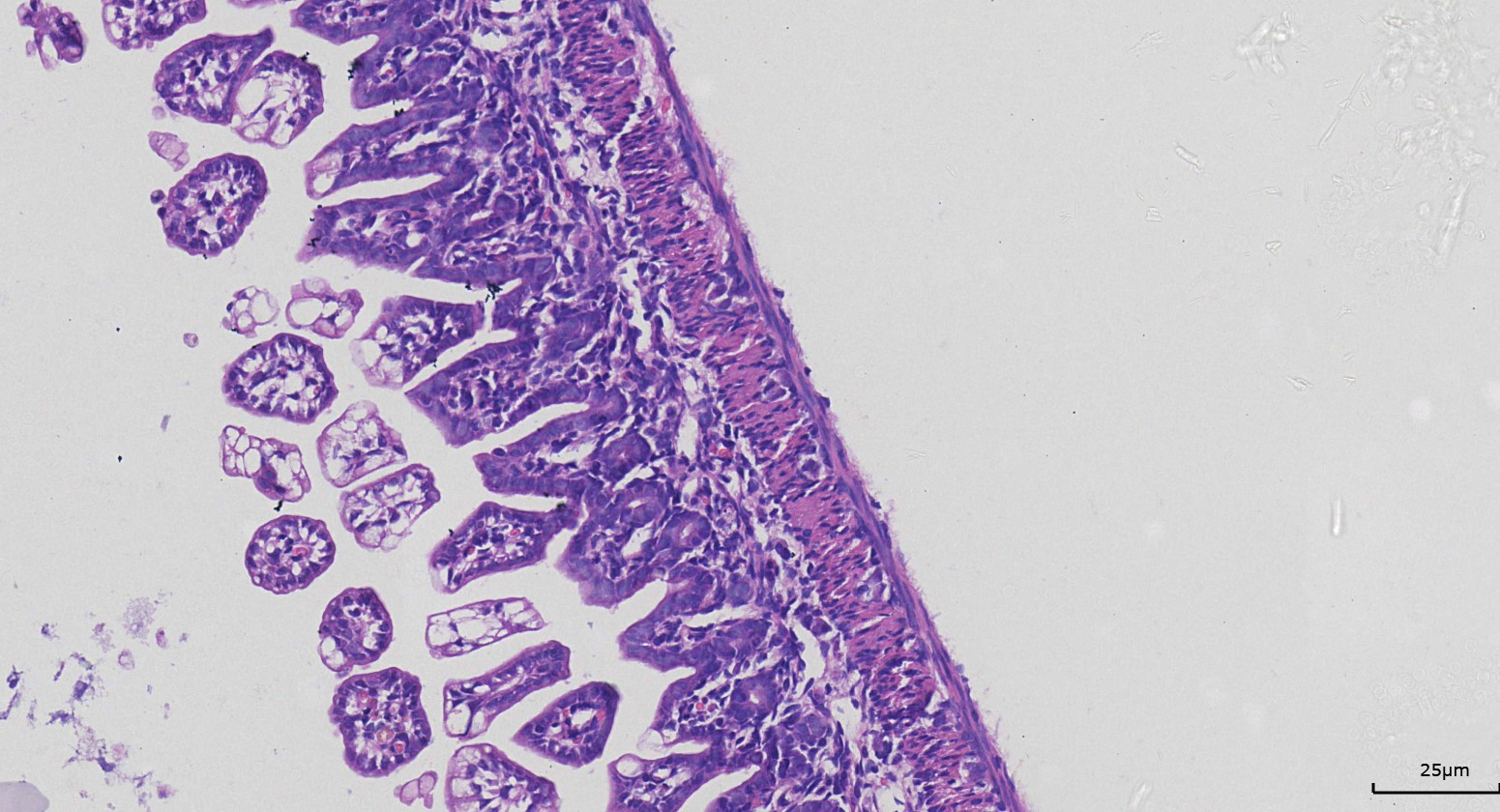

Supplement: S2 File — (ZIP) [file pone.0324068.s002.zip › S2-File/NEC+1-MNA/NEC+1-MNA 5.tiff]

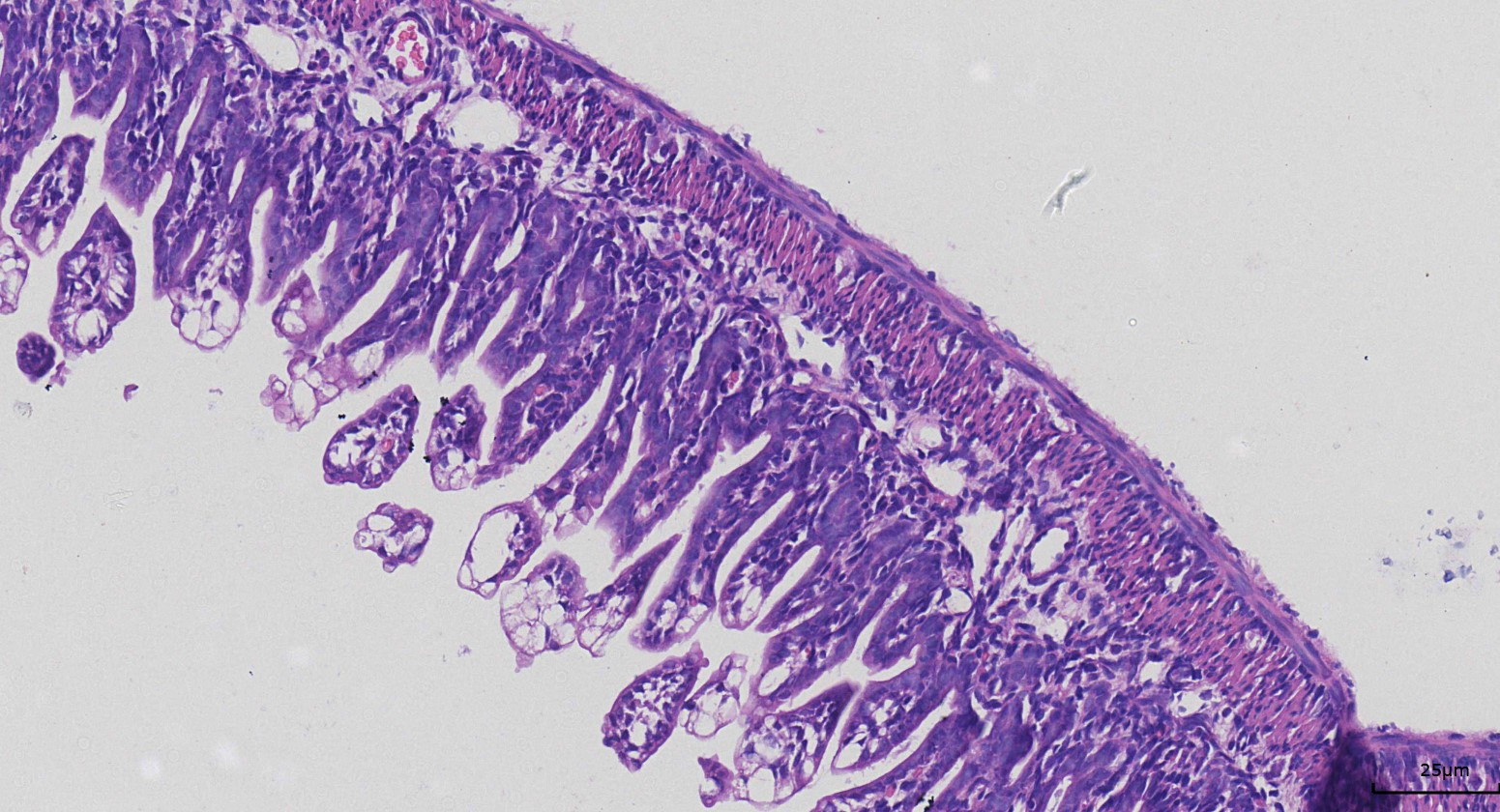

Supplement: S2 File — (ZIP) [file pone.0324068.s002.zip › S2-File/NEC+1-MNA/NEC+1-MNA 6.tiff]

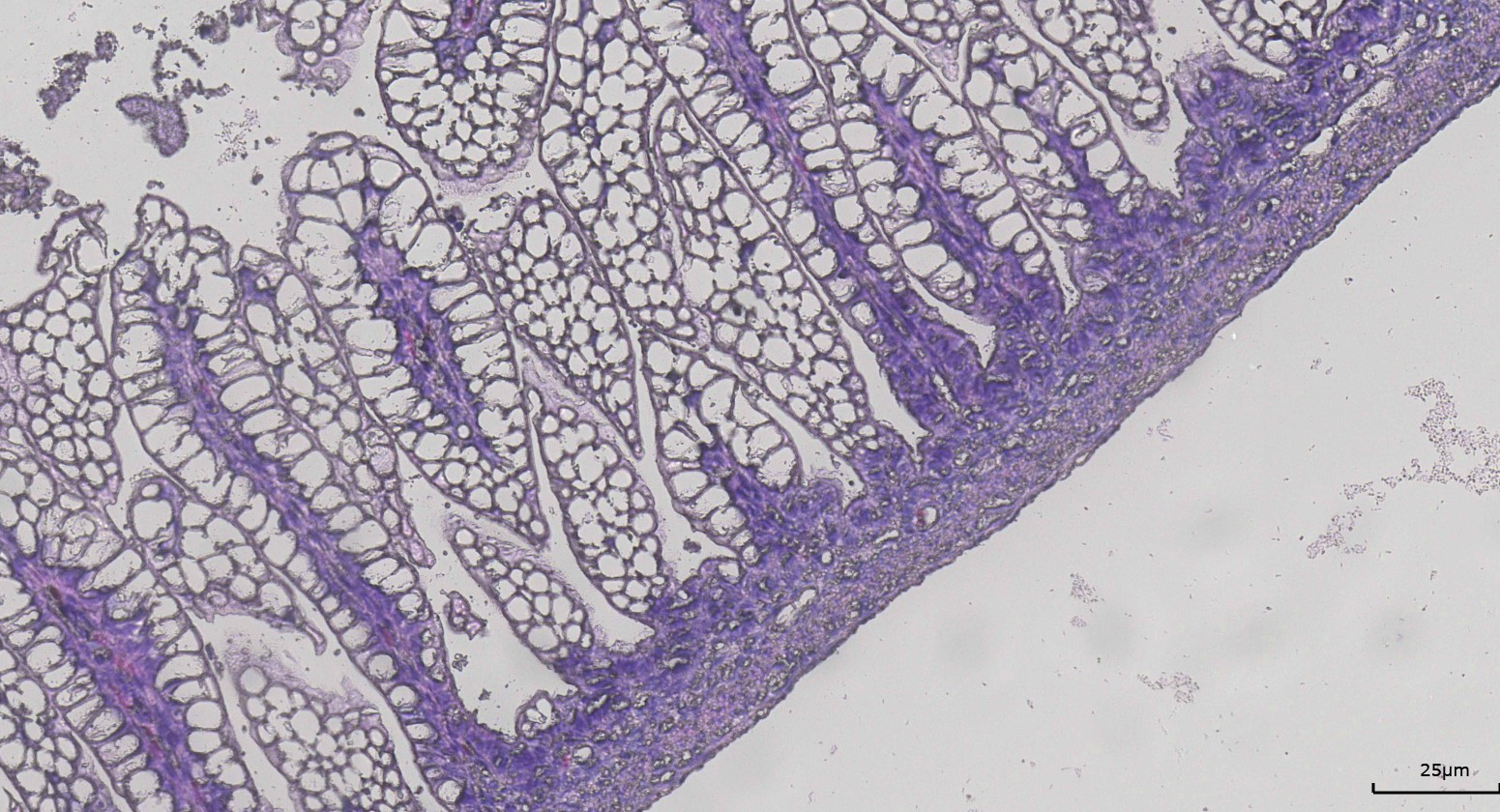

Supplement: S2 File — (ZIP) [file pone.0324068.s002.zip › S2-File/NEC+1-MNA/NEC+1-MNA 7.tiff]

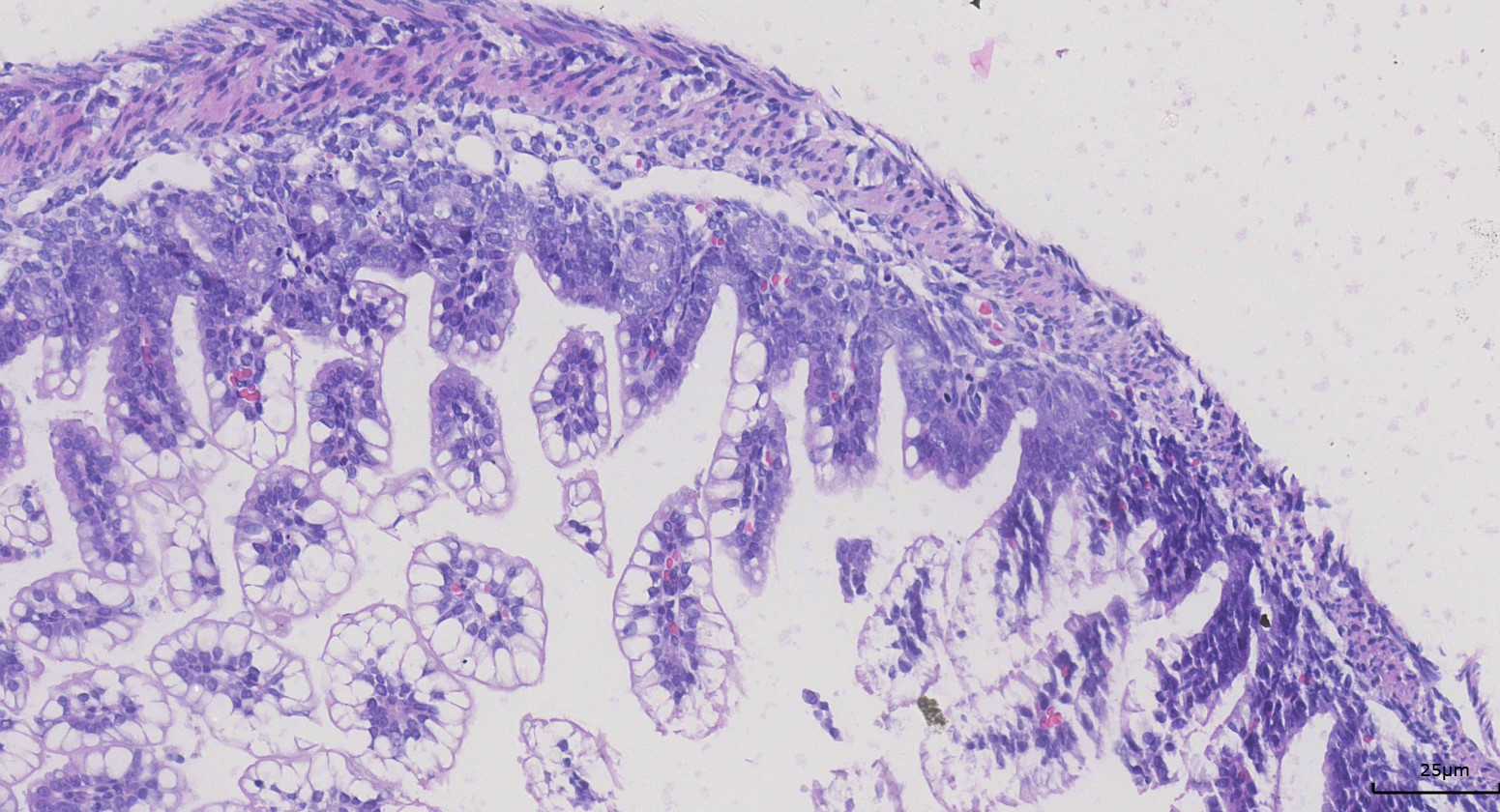

Supplement: S2 File — (ZIP) [file pone.0324068.s002.zip › S2-File/NEC+1-MNA/NEC+1-MNA 8.tiff]

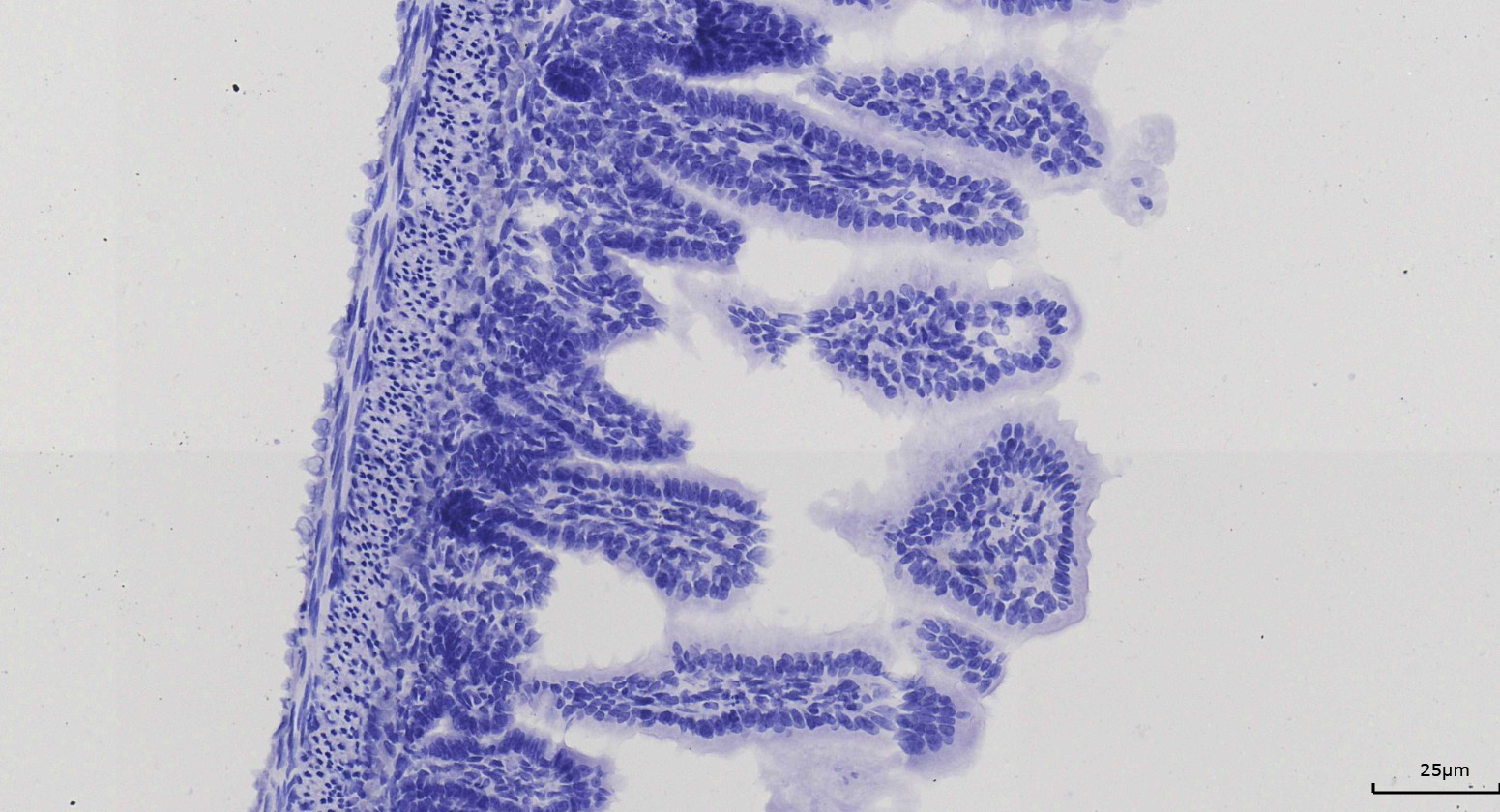

Supplement: S3 File — (ZIP) [file pone.0324068.s003.zip › S3-File/Control.tiff]

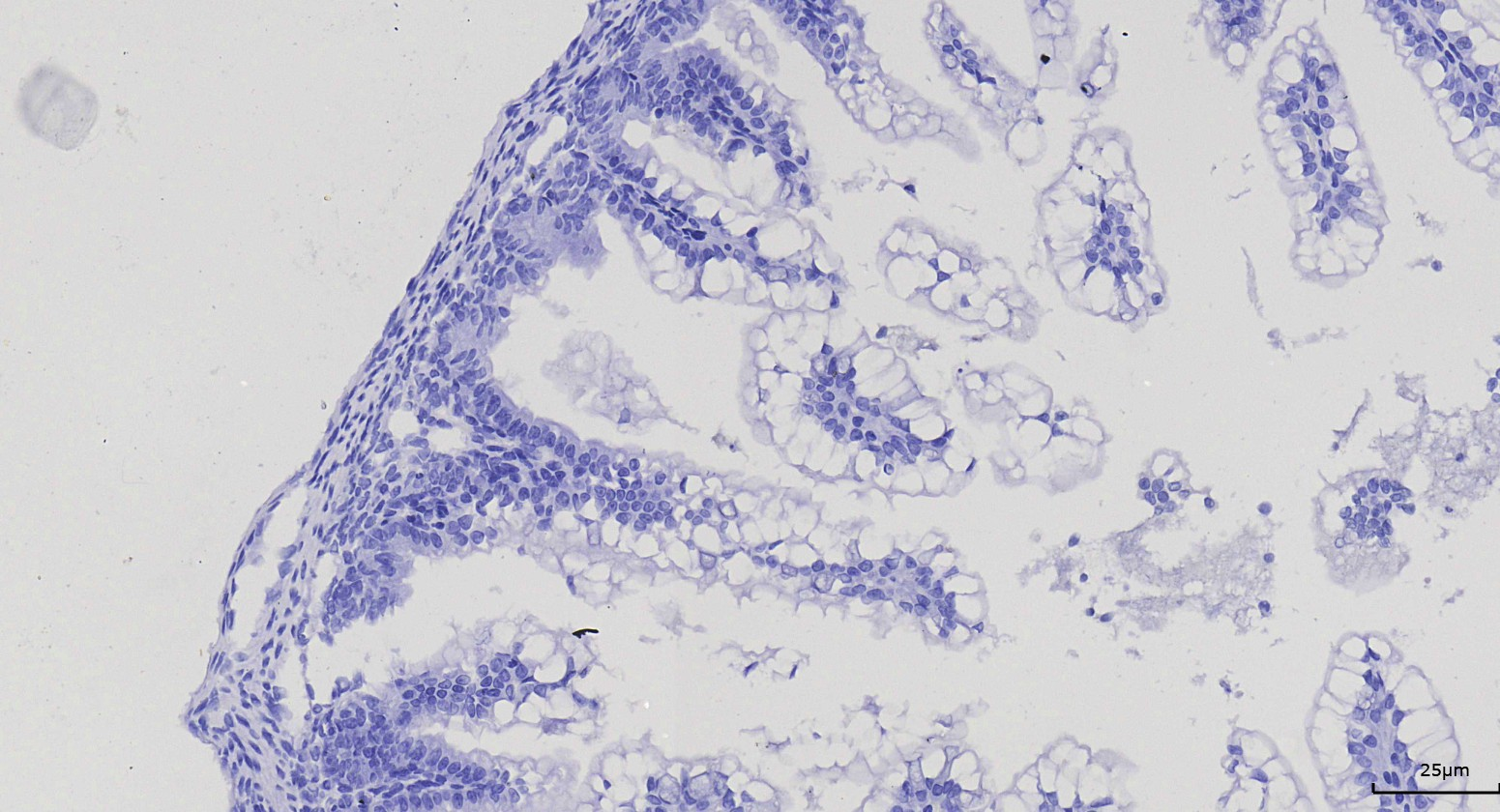

Supplement: S3 File — (ZIP) [file pone.0324068.s003.zip › S3-File/NEC+1-MNA.tiff]

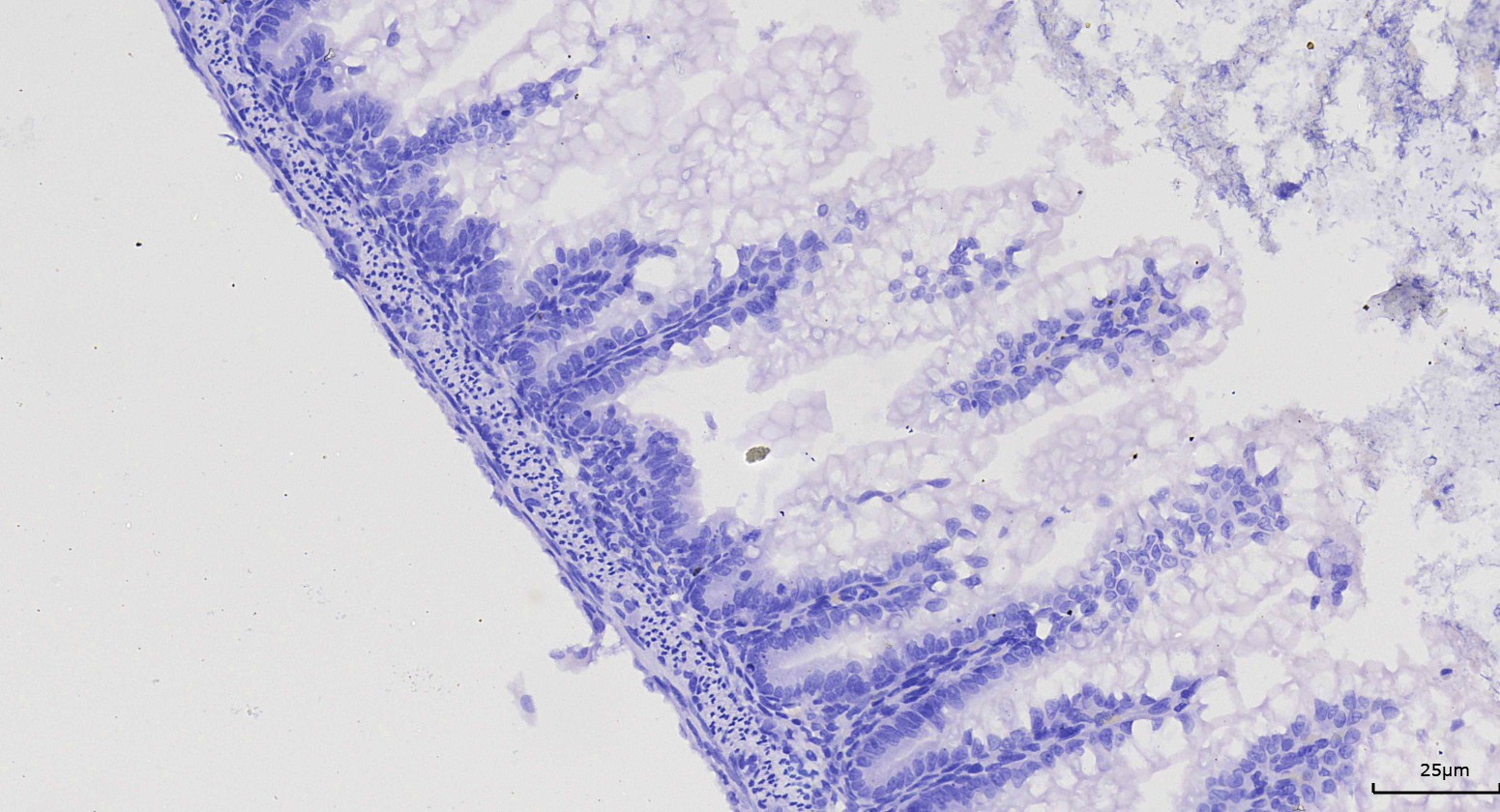

Supplement: S3 File — (ZIP) [file pone.0324068.s003.zip › S3-File/NEC.tiff]

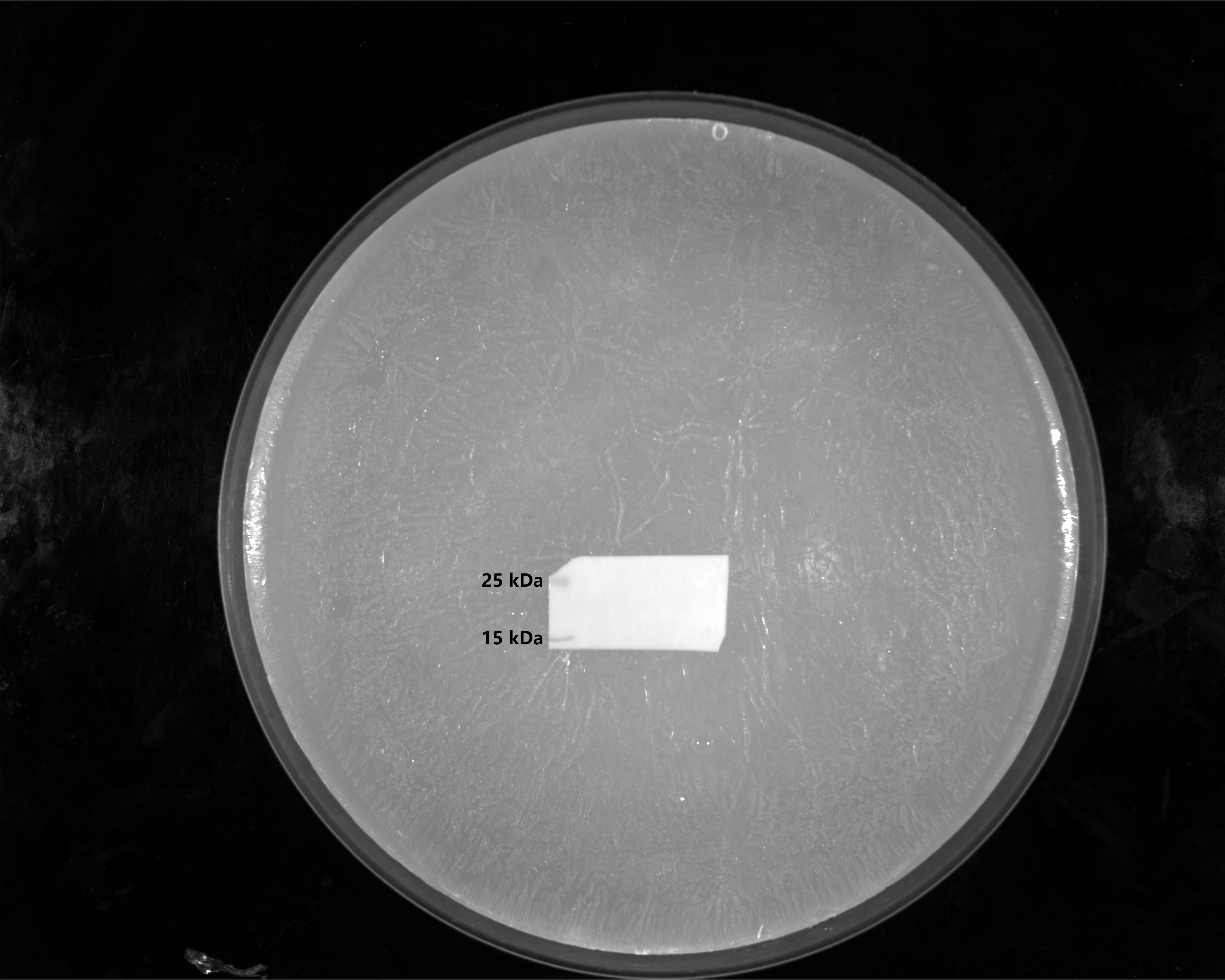

Supplement: S4 File — (ZIP) [file pone.0324068.s004.zip › NNMT(Figure I)/human NNMT/NNMT(Human)1 marker.png]

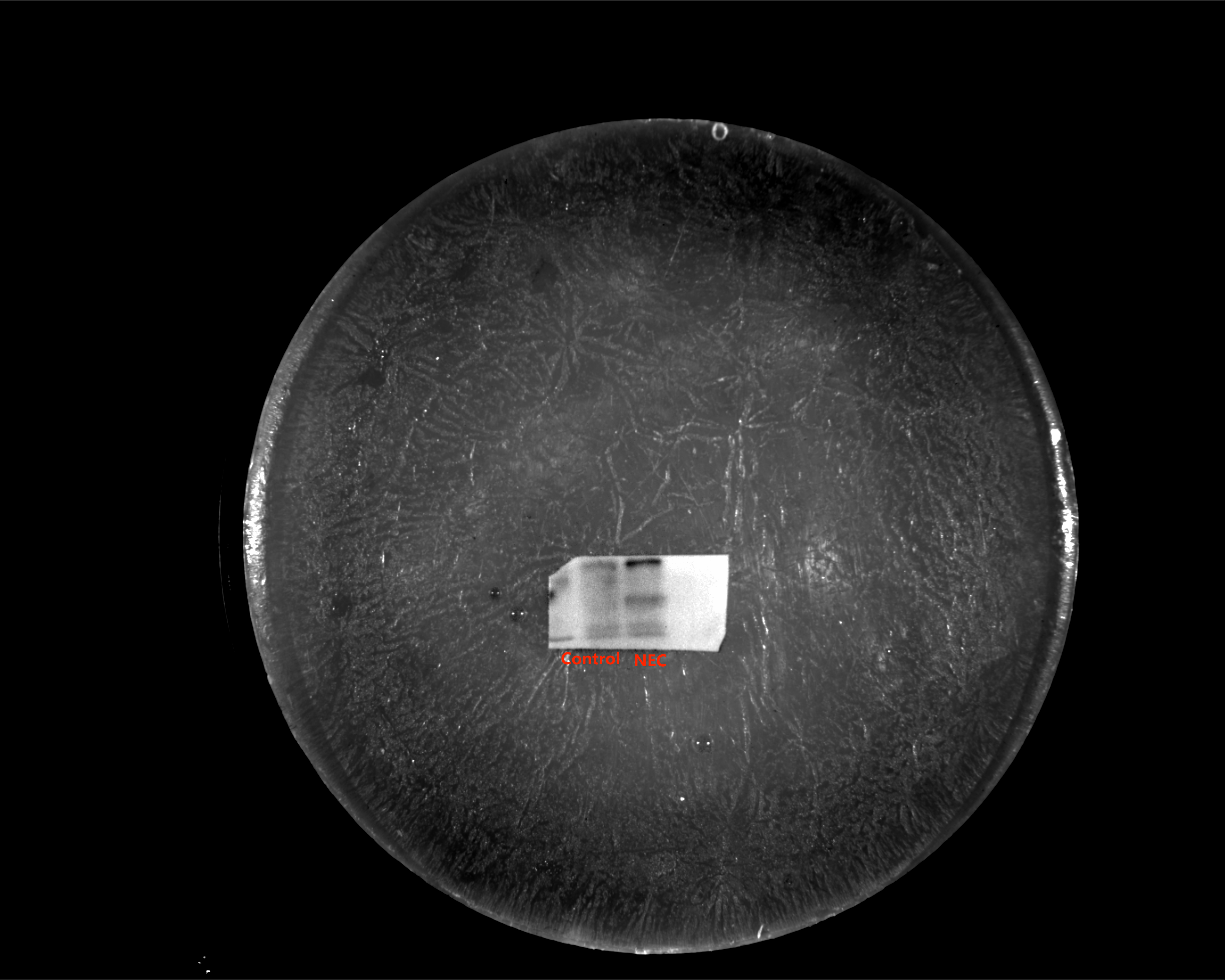

Supplement: S4 File — (ZIP) [file pone.0324068.s004.zip › NNMT(Figure I)/human NNMT/NNMT(Human)1.png]

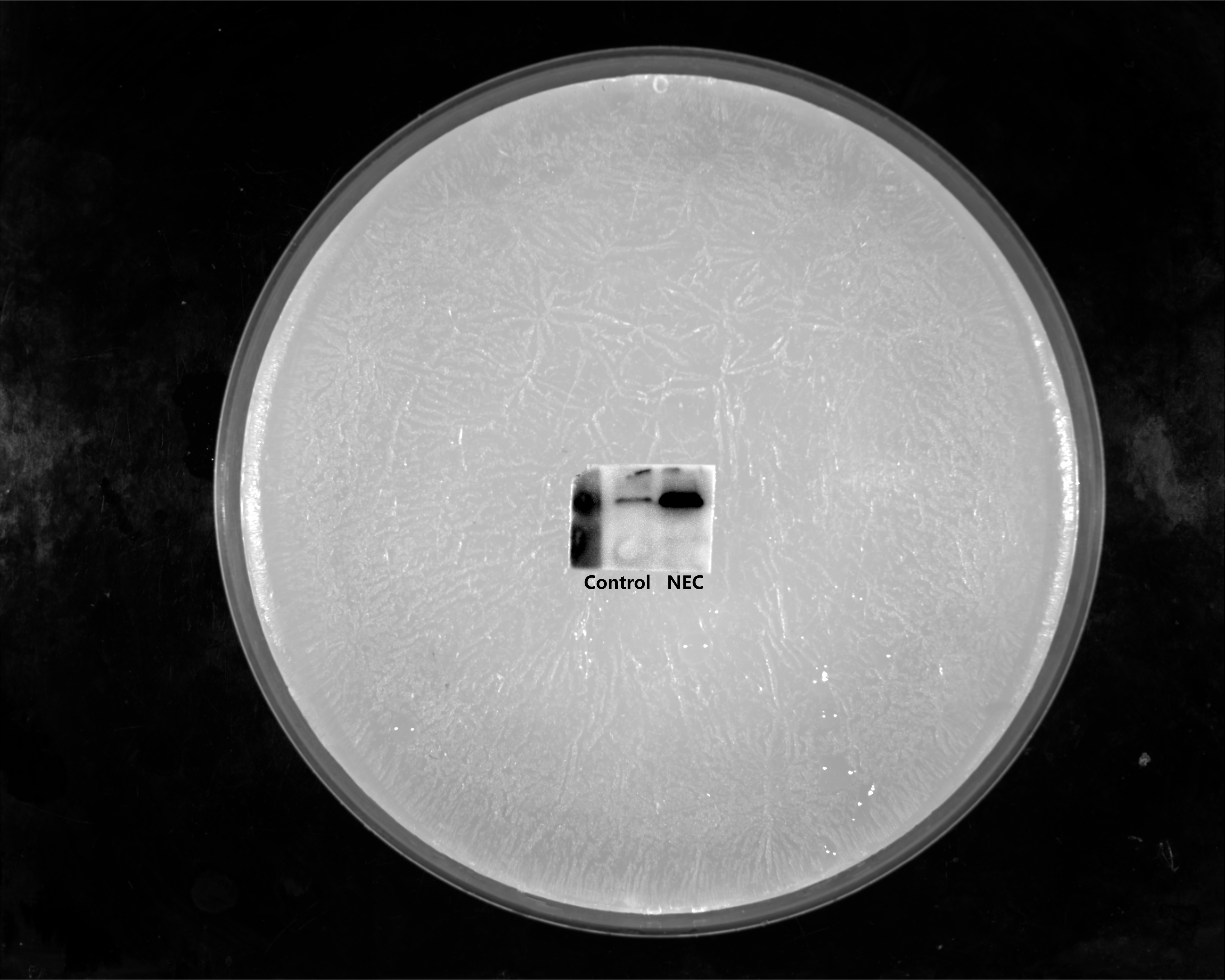

Supplement: S4 File — (ZIP) [file pone.0324068.s004.zip › NNMT(Figure I)/human NNMT/NNMT(Human)2 .png]

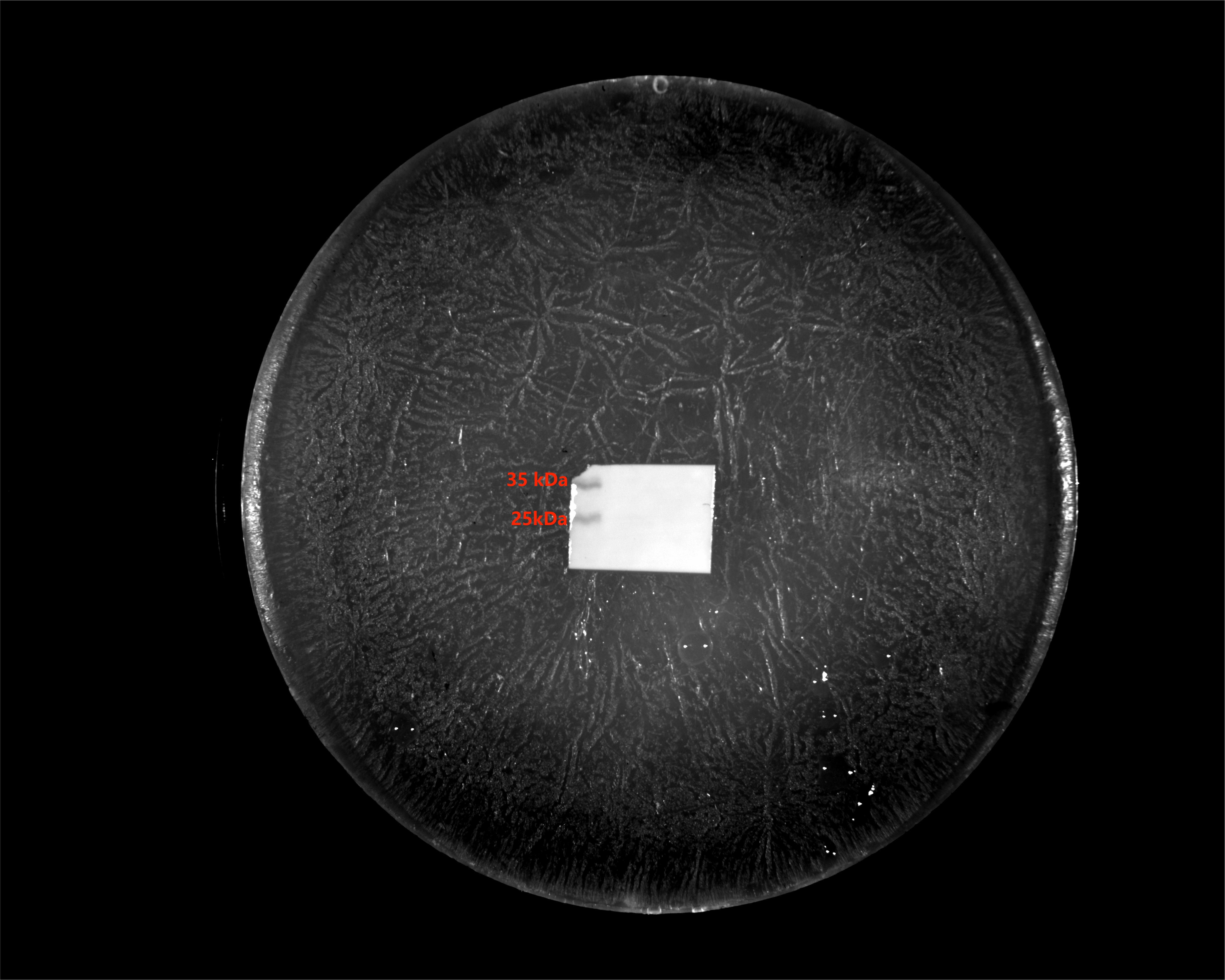

Supplement: S4 File — (ZIP) [file pone.0324068.s004.zip › NNMT(Figure I)/human NNMT/NNMT(Human)2 marker.png]

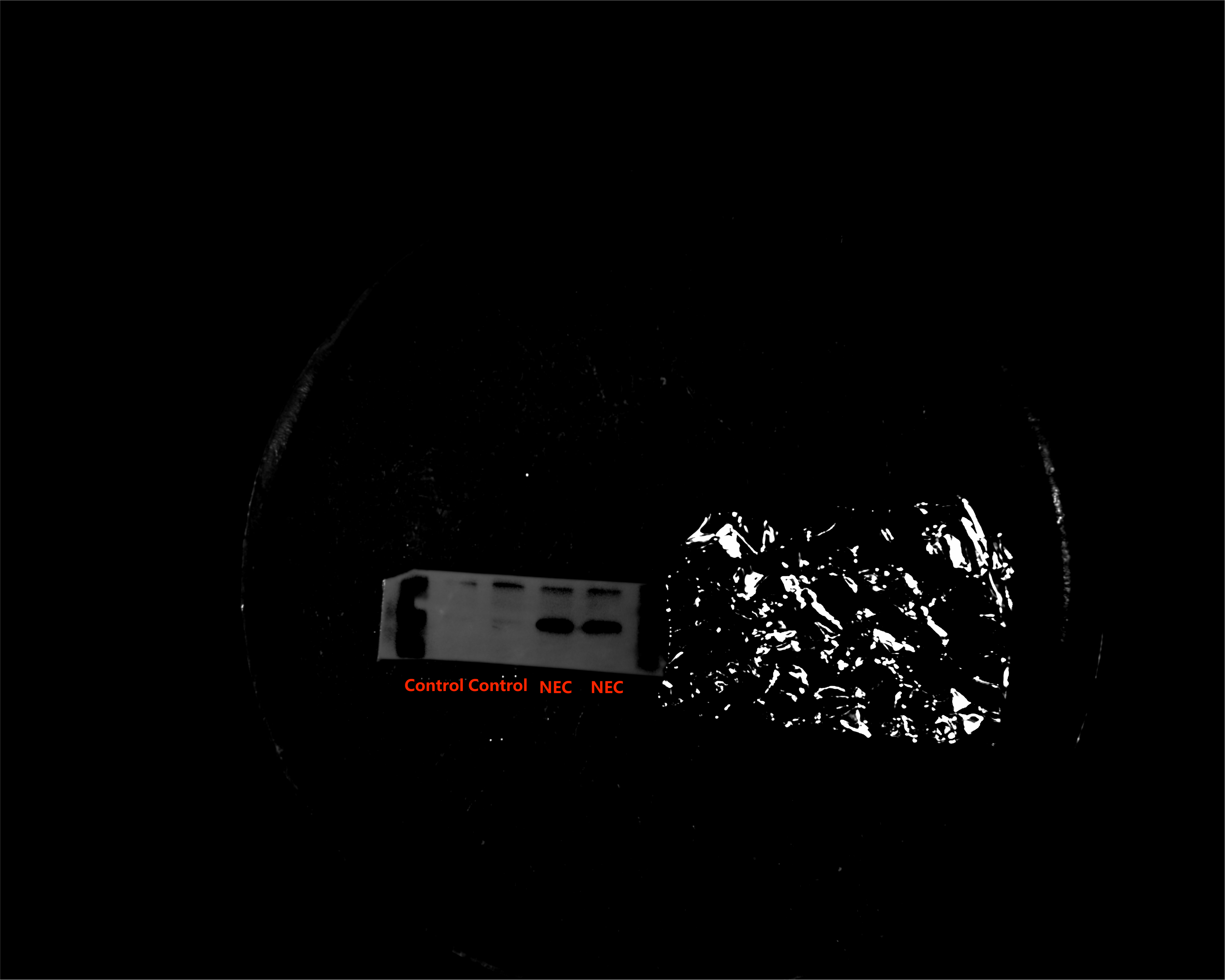

Supplement: S4 File — (ZIP) [file pone.0324068.s004.zip › NNMT(Figure I)/human NNMT/NNMT(Human)3 .png]

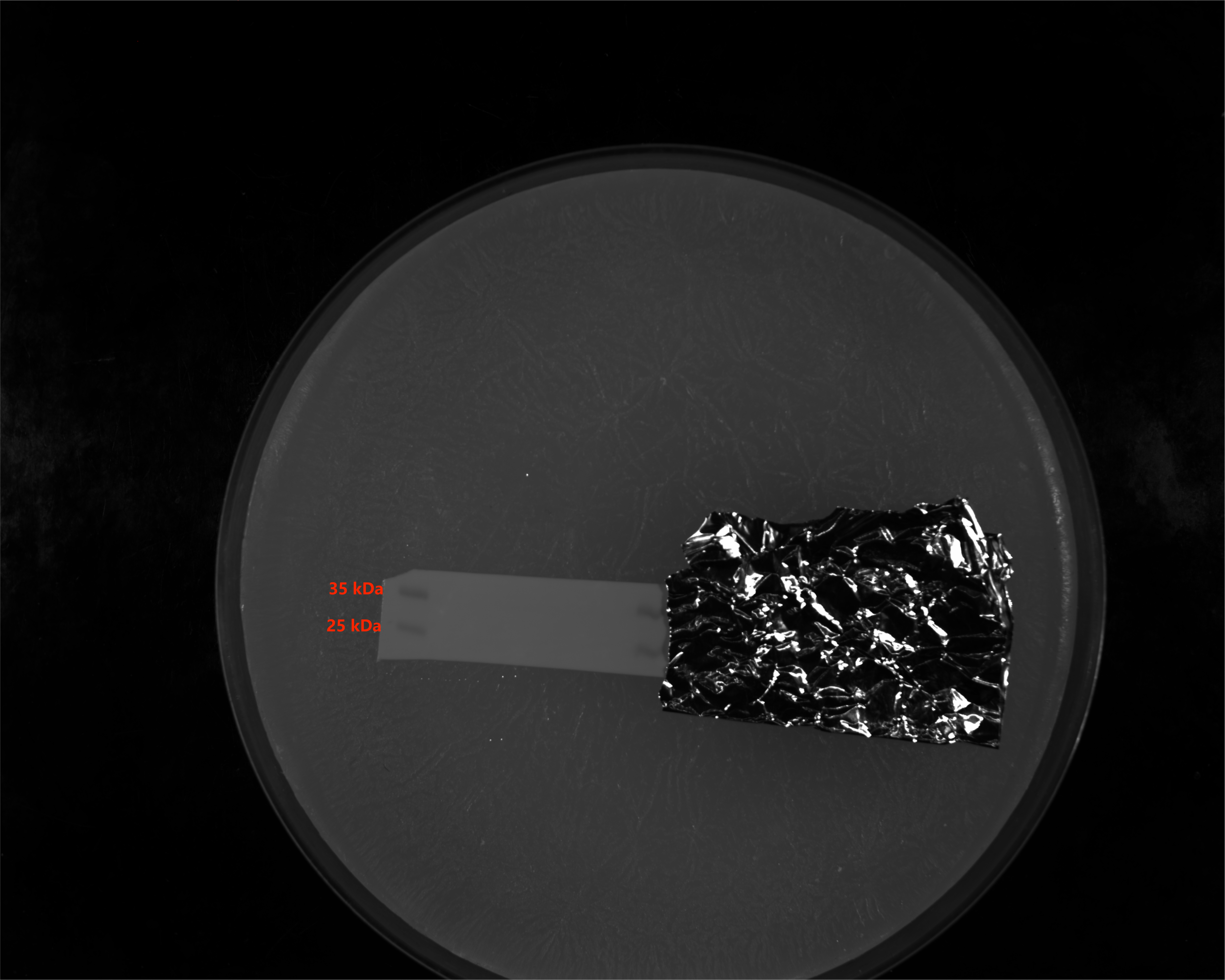

Supplement: S4 File — (ZIP) [file pone.0324068.s004.zip › NNMT(Figure I)/human NNMT/NNMT(Human)3 marker .png]

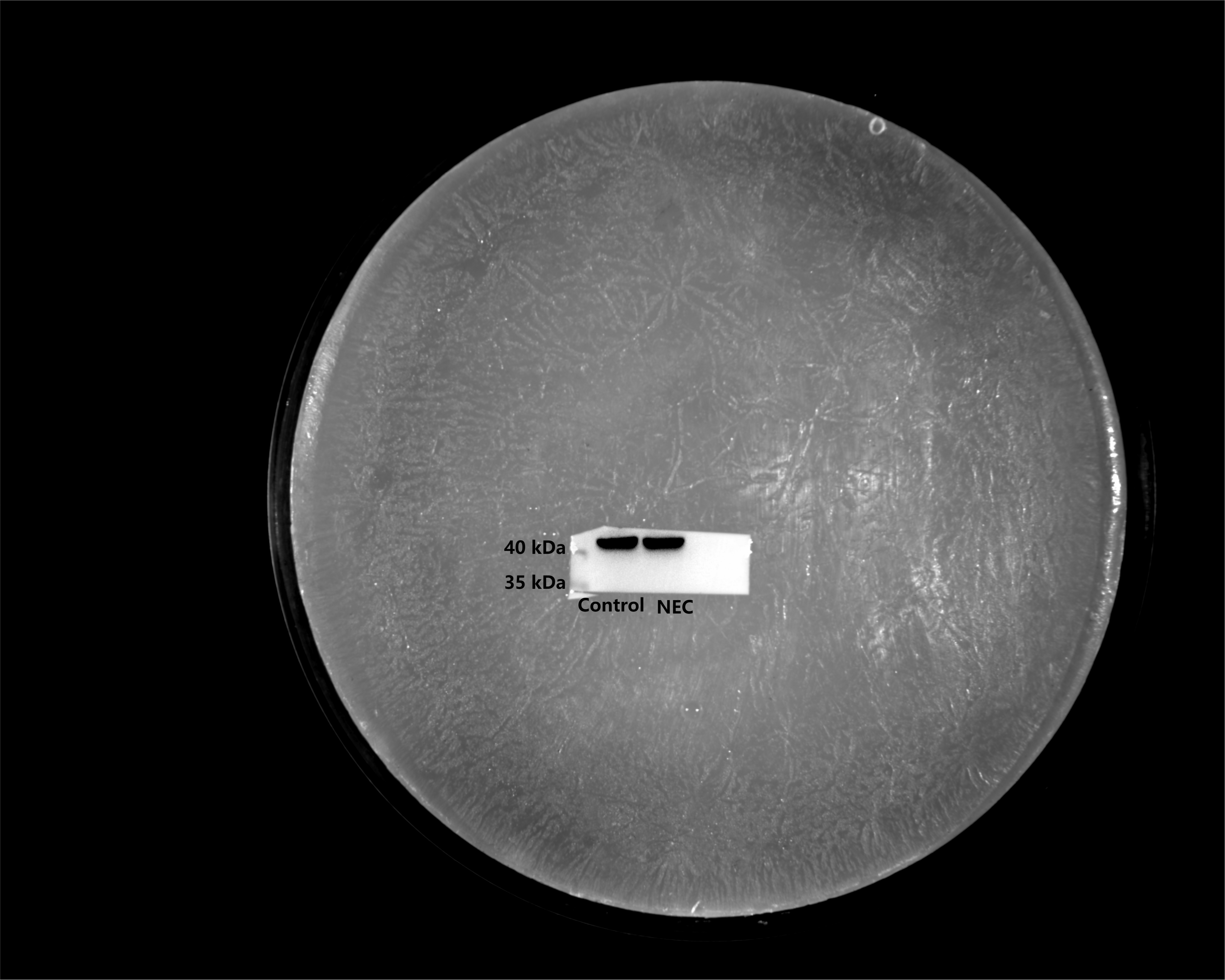

Supplement: S4 File — (ZIP) [file pone.0324068.s004.zip › NNMT(Figure I)/human NNMT/β-actin for NNMT(Human) 1.png]

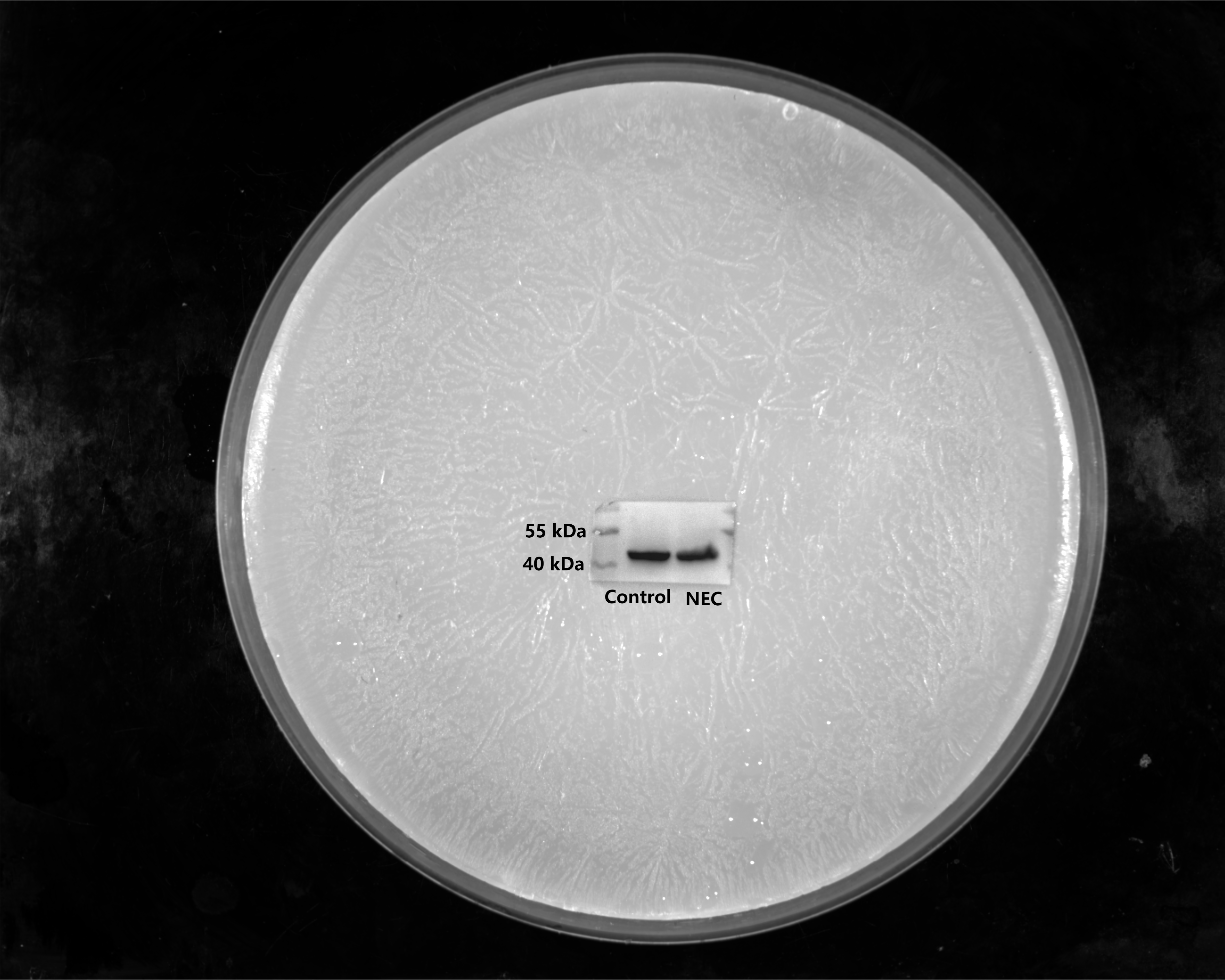

Supplement: S4 File — (ZIP) [file pone.0324068.s004.zip › NNMT(Figure I)/human NNMT/β-actin for NNMT(Human)2.png]

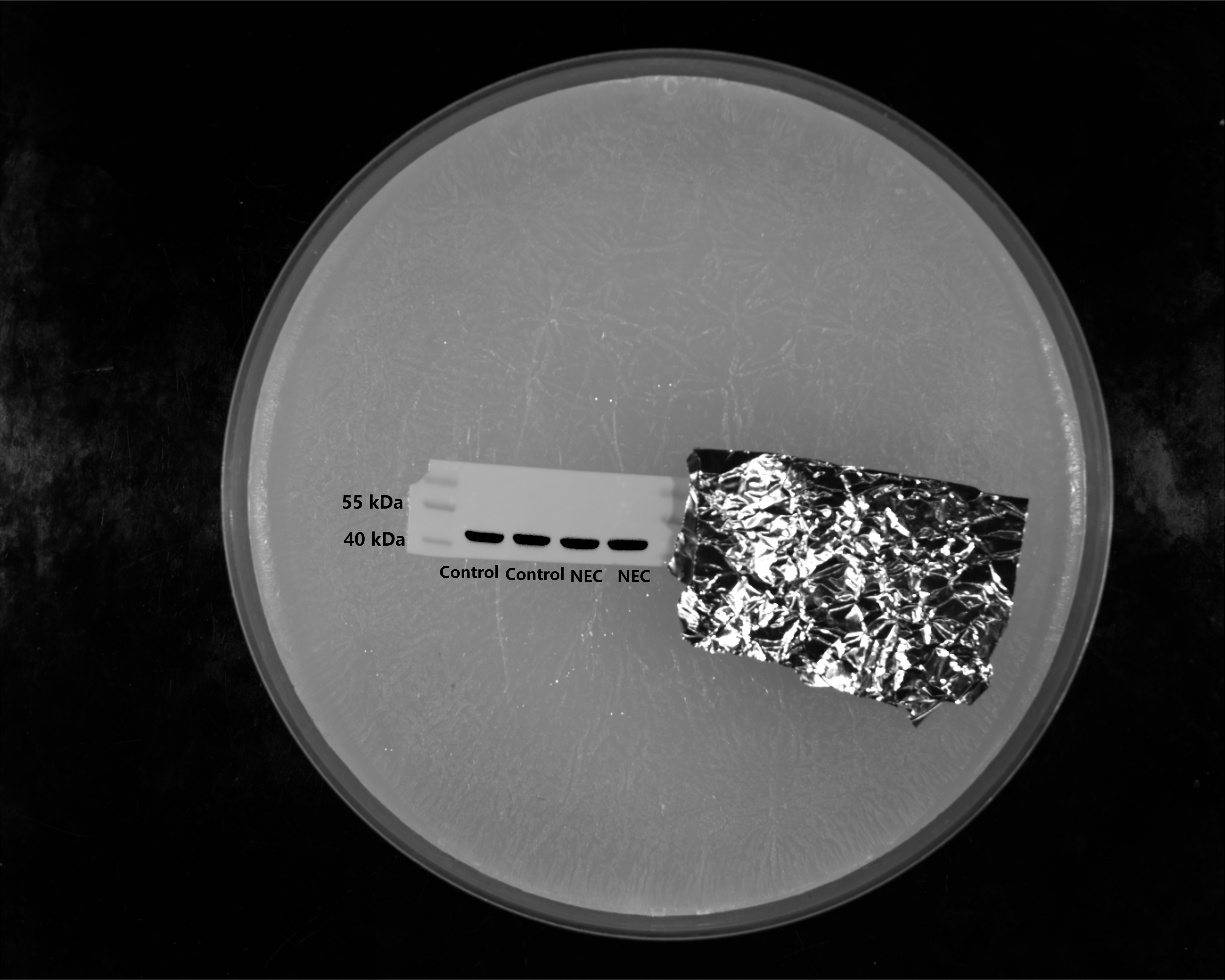

Supplement: S4 File — (ZIP) [file pone.0324068.s004.zip › NNMT(Figure I)/human NNMT/β-actin for NNMT(Human)3.png]

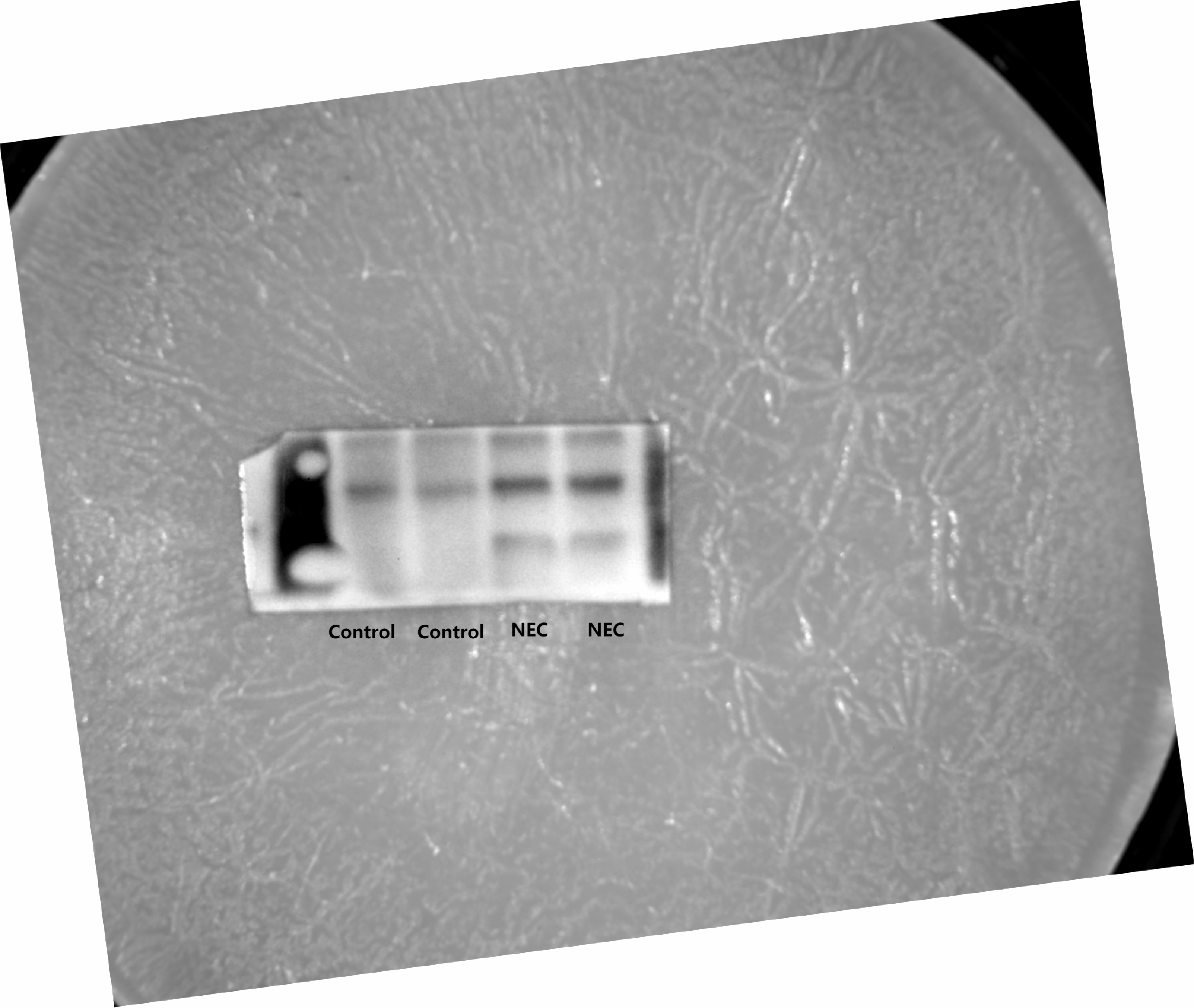

Supplement: S4 File — (ZIP) [file pone.0324068.s004.zip › NNMT(Figure I)/Rat NNMT/NNMT (Rat) 1.png]

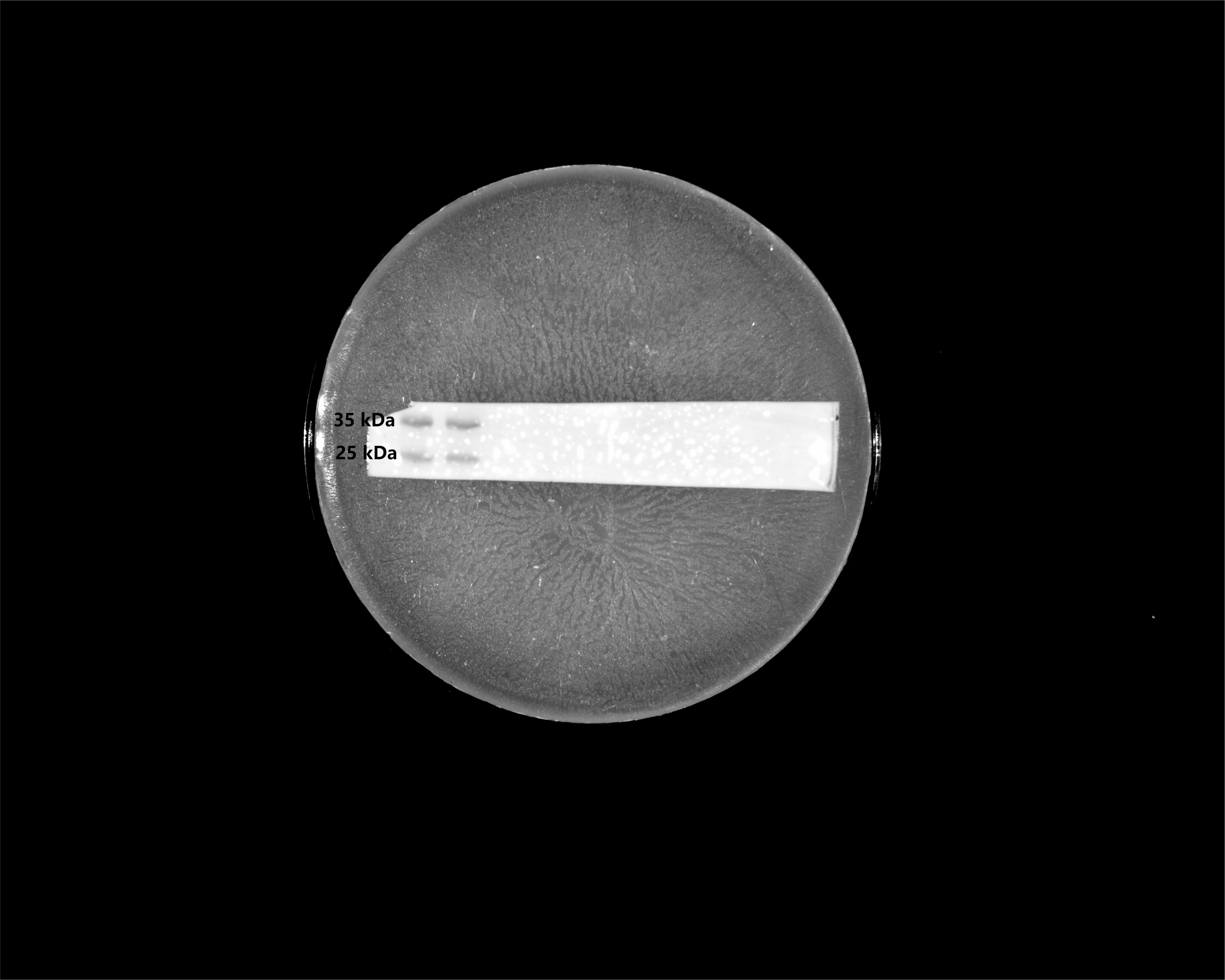

Supplement: S4 File — (ZIP) [file pone.0324068.s004.zip › NNMT(Figure I)/Rat NNMT/NNMT (Rat) 2 marker.png]

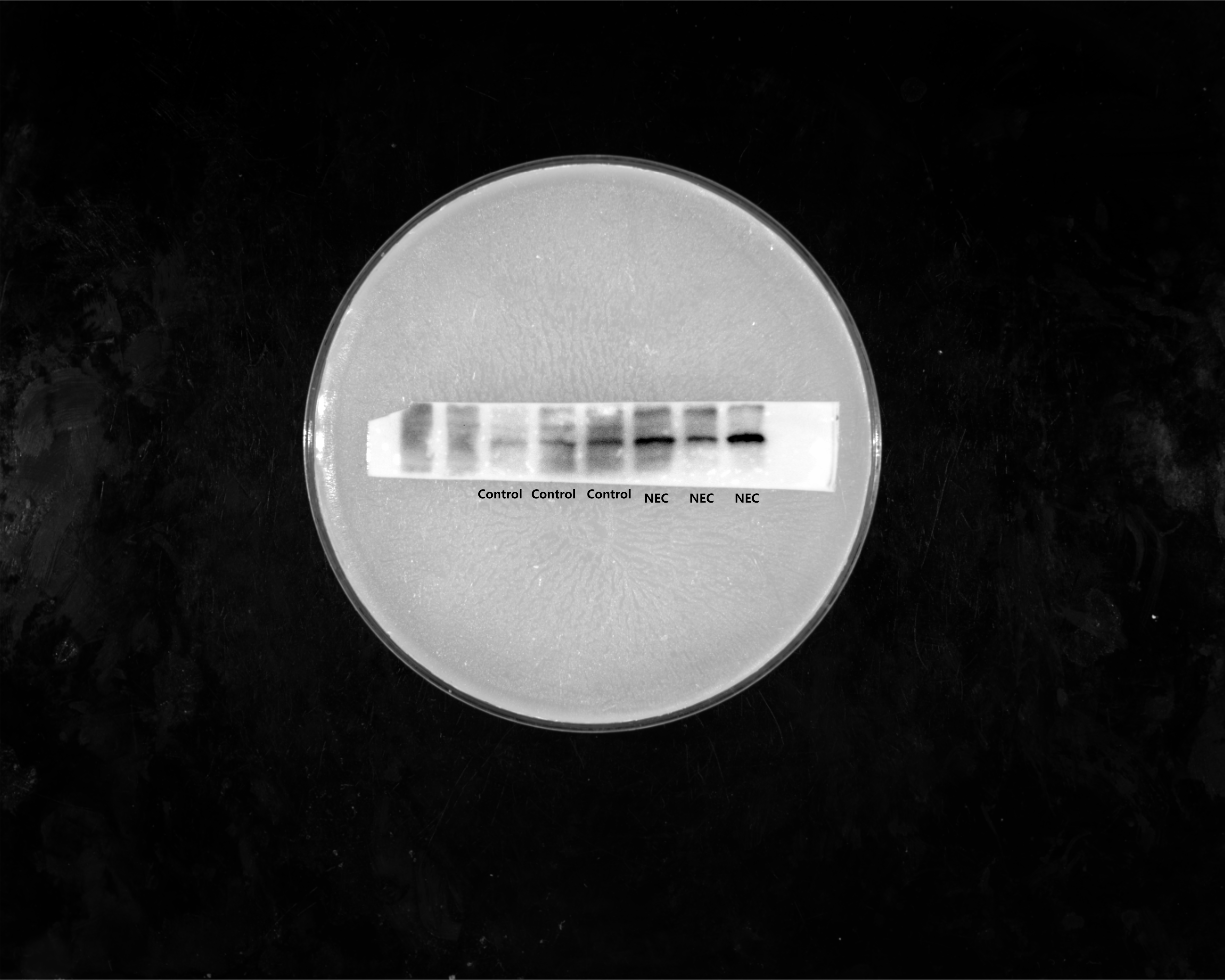

Supplement: S4 File — (ZIP) [file pone.0324068.s004.zip › NNMT(Figure I)/Rat NNMT/NNMT (Rat) 2.png]

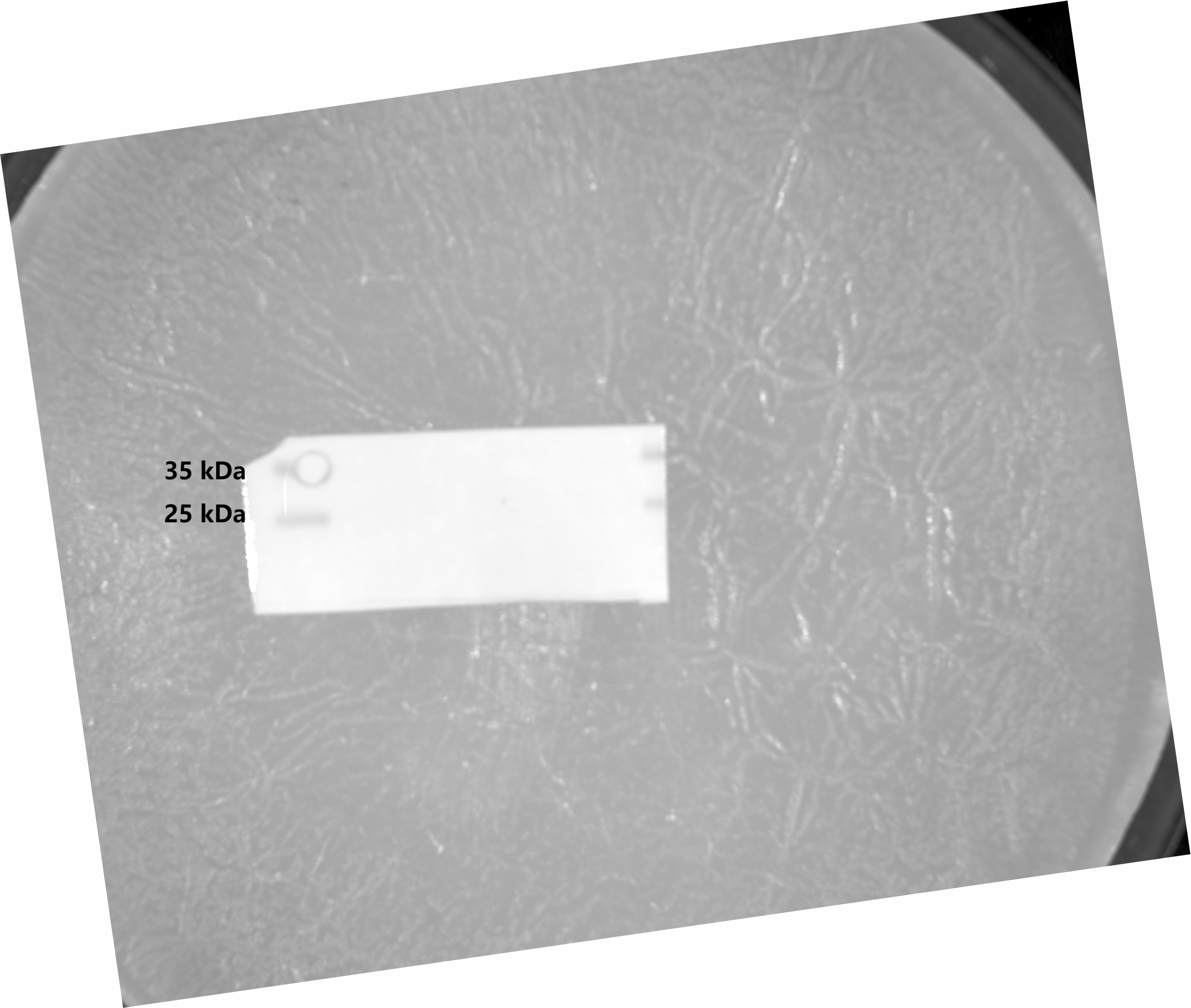

Supplement: S4 File — (ZIP) [file pone.0324068.s004.zip › NNMT(Figure I)/Rat NNMT/NNMT(Rat)1 marker.png]

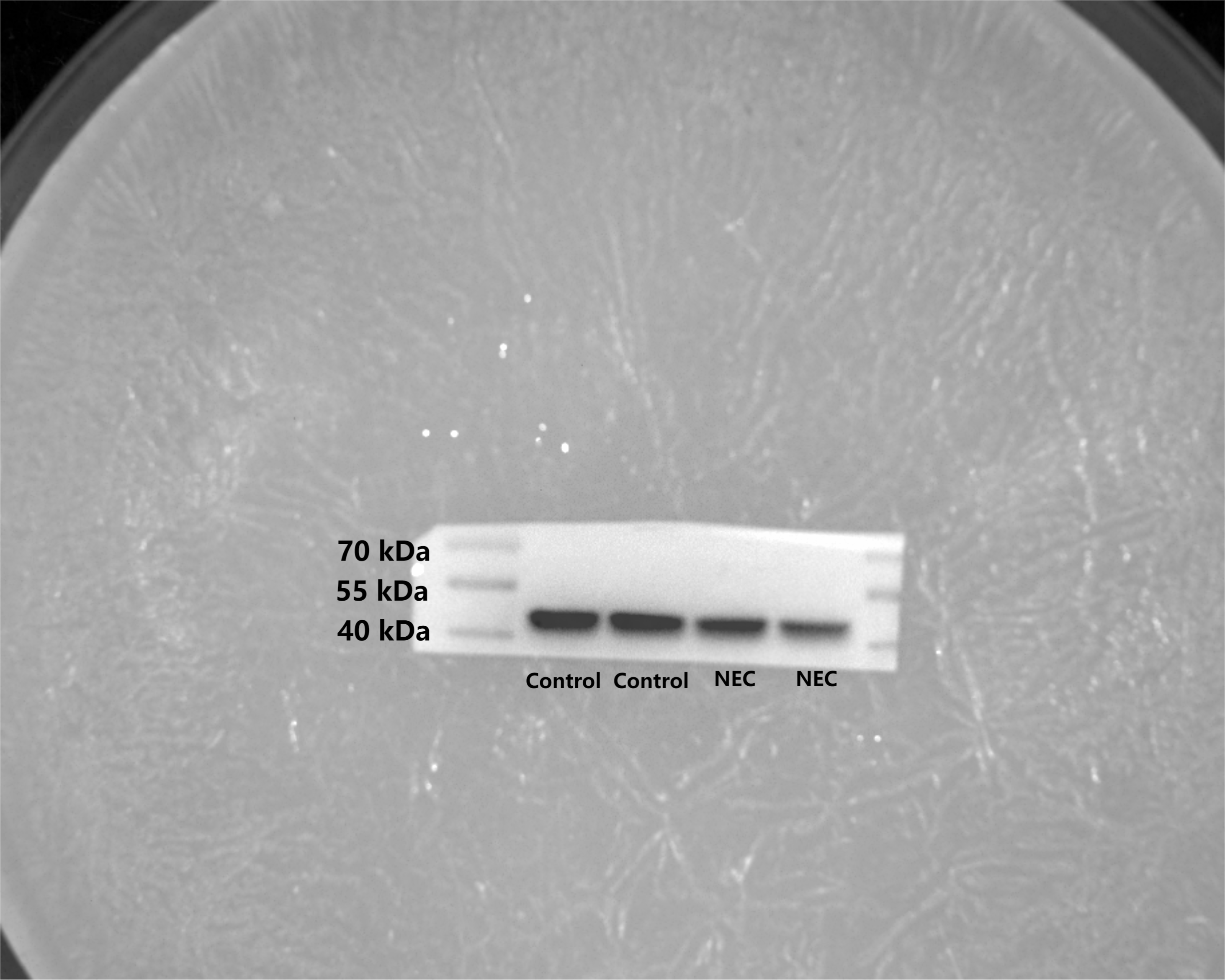

Supplement: S4 File — (ZIP) [file pone.0324068.s004.zip › NNMT(Figure I)/Rat NNMT/β-actin for NNMT(Rat) 1.png]

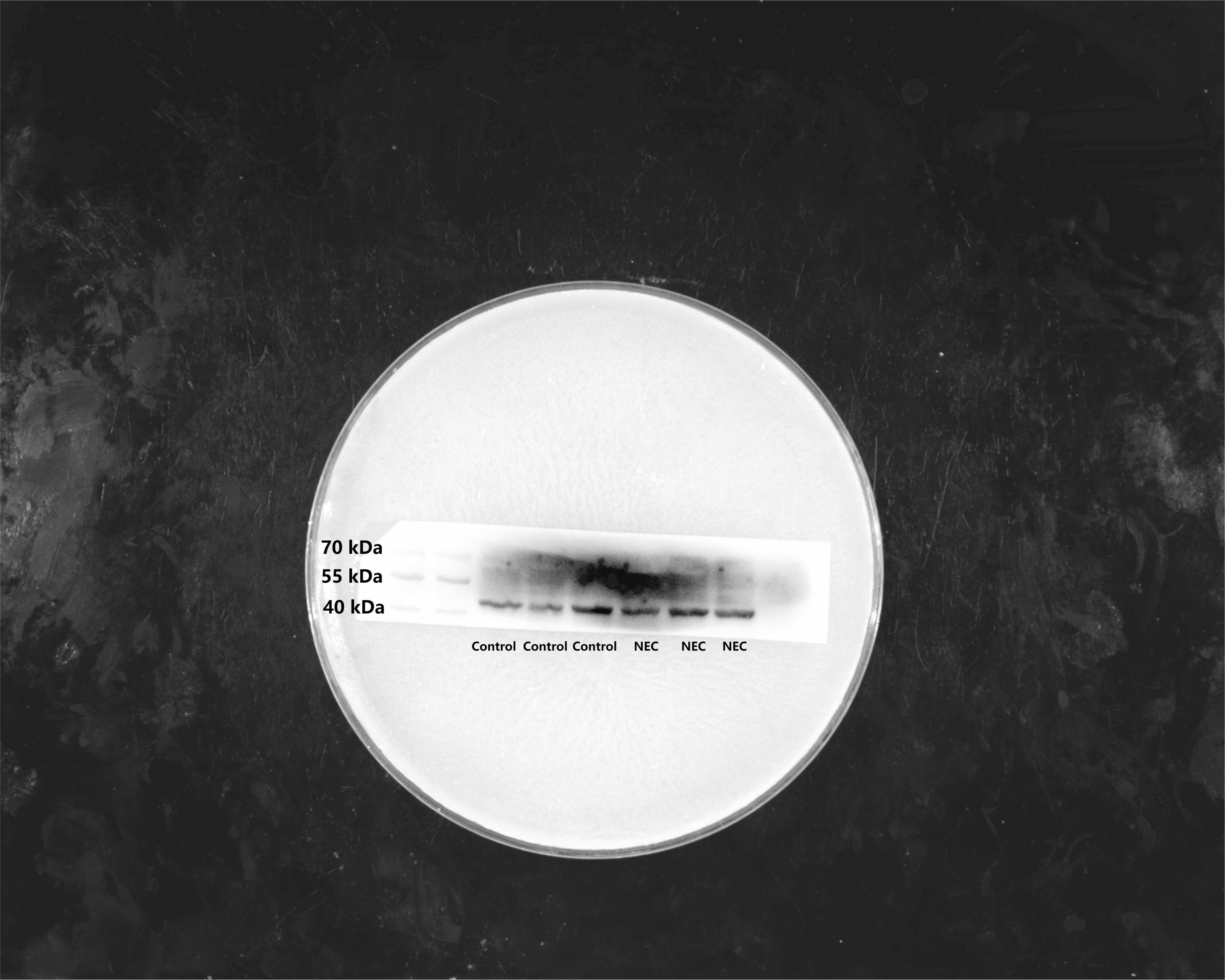

Supplement: S4 File — (ZIP) [file pone.0324068.s004.zip › NNMT(Figure I)/Rat NNMT/β-actin for NNMT(Rat)2.png]

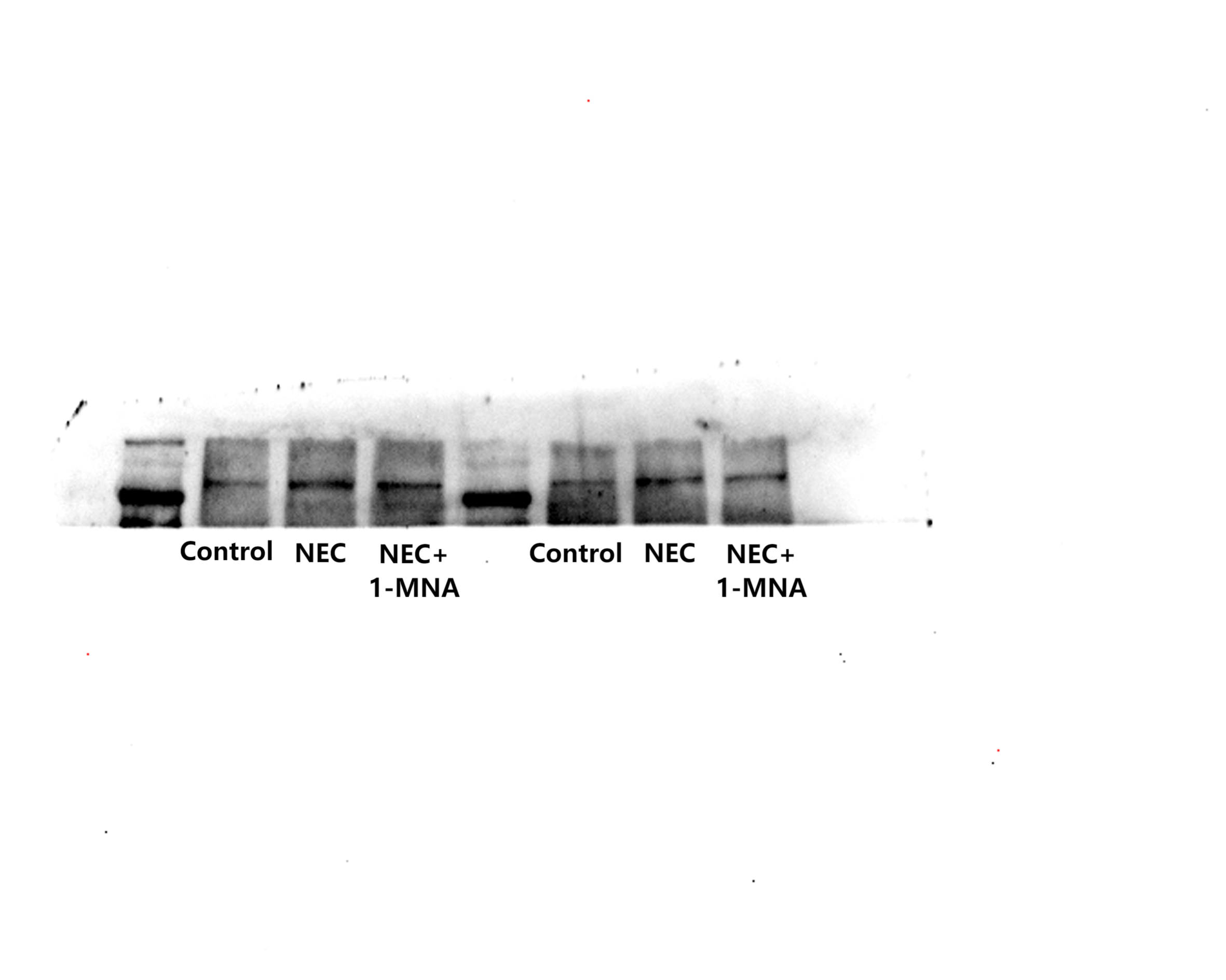

Supplement: S5 File — (ZIP) [file pone.0324068.s005.zip › TLR4(Figure IV)/TLR4-1.png]

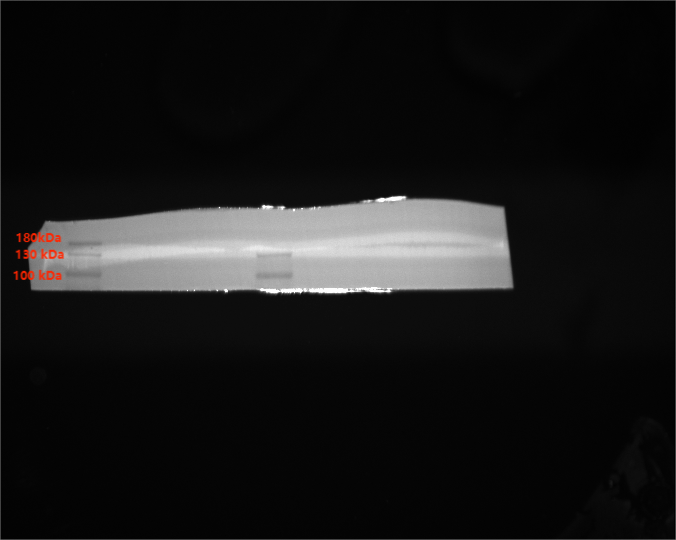

Supplement: S5 File — (ZIP) [file pone.0324068.s005.zip › TLR4(Figure IV)/TLR4-1_marker.png]

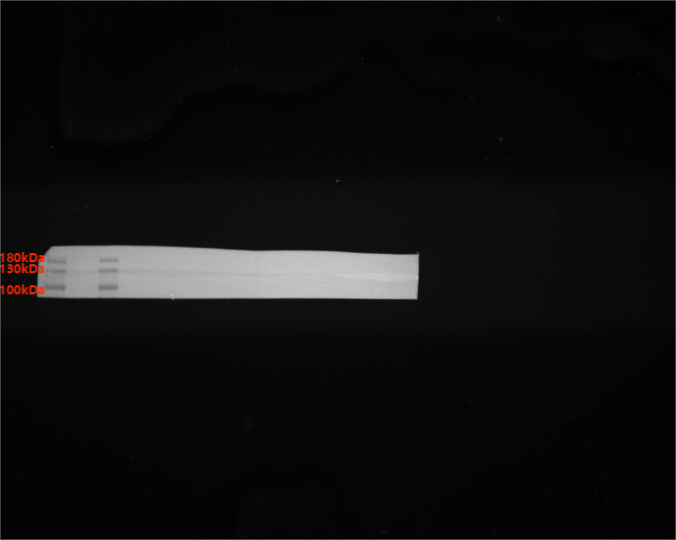

Supplement: S5 File — (ZIP) [file pone.0324068.s005.zip › TLR4(Figure IV)/TLR4-2 marker.png]

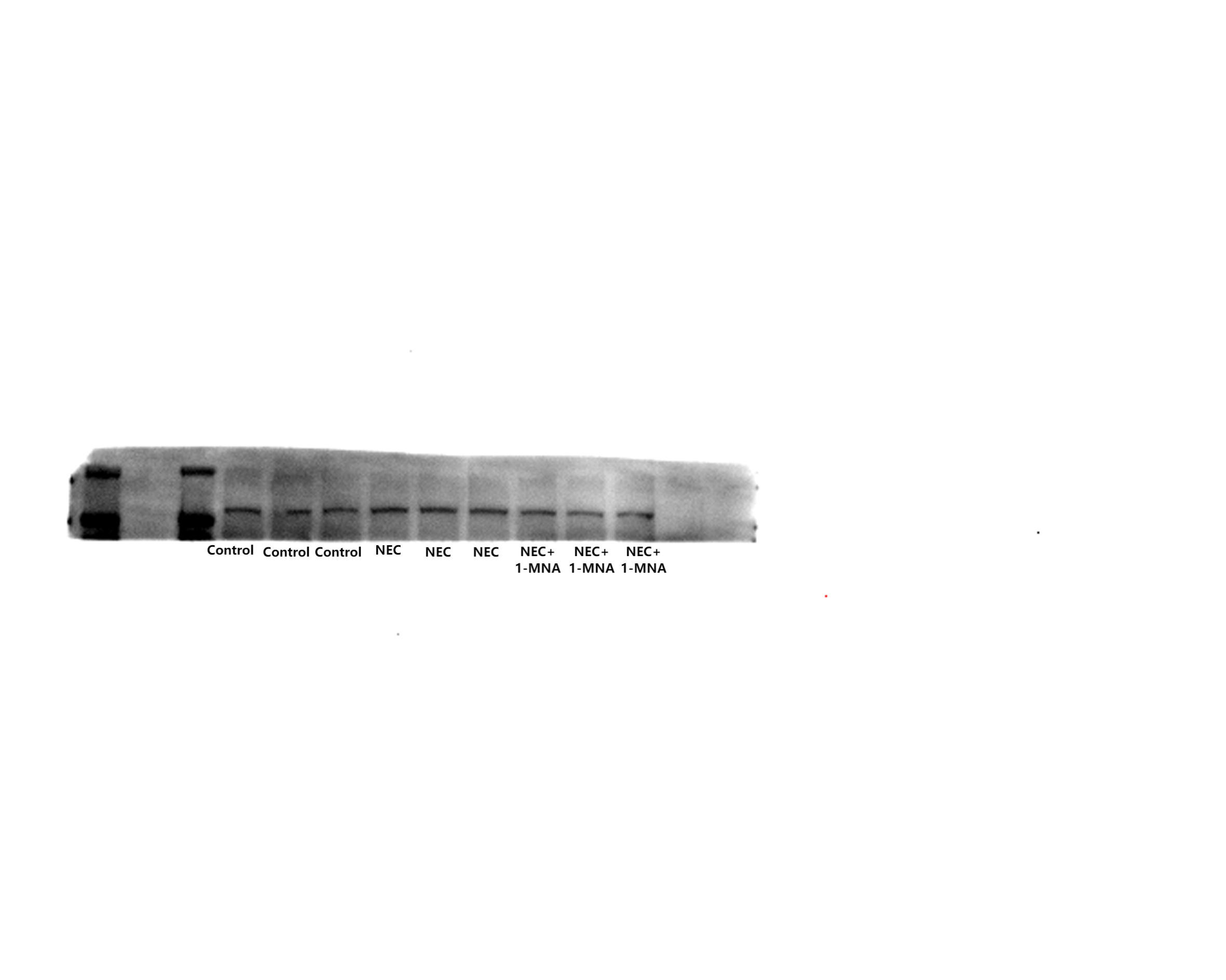

Supplement: S5 File — (ZIP) [file pone.0324068.s005.zip › TLR4(Figure IV)/TLR4-2.png]

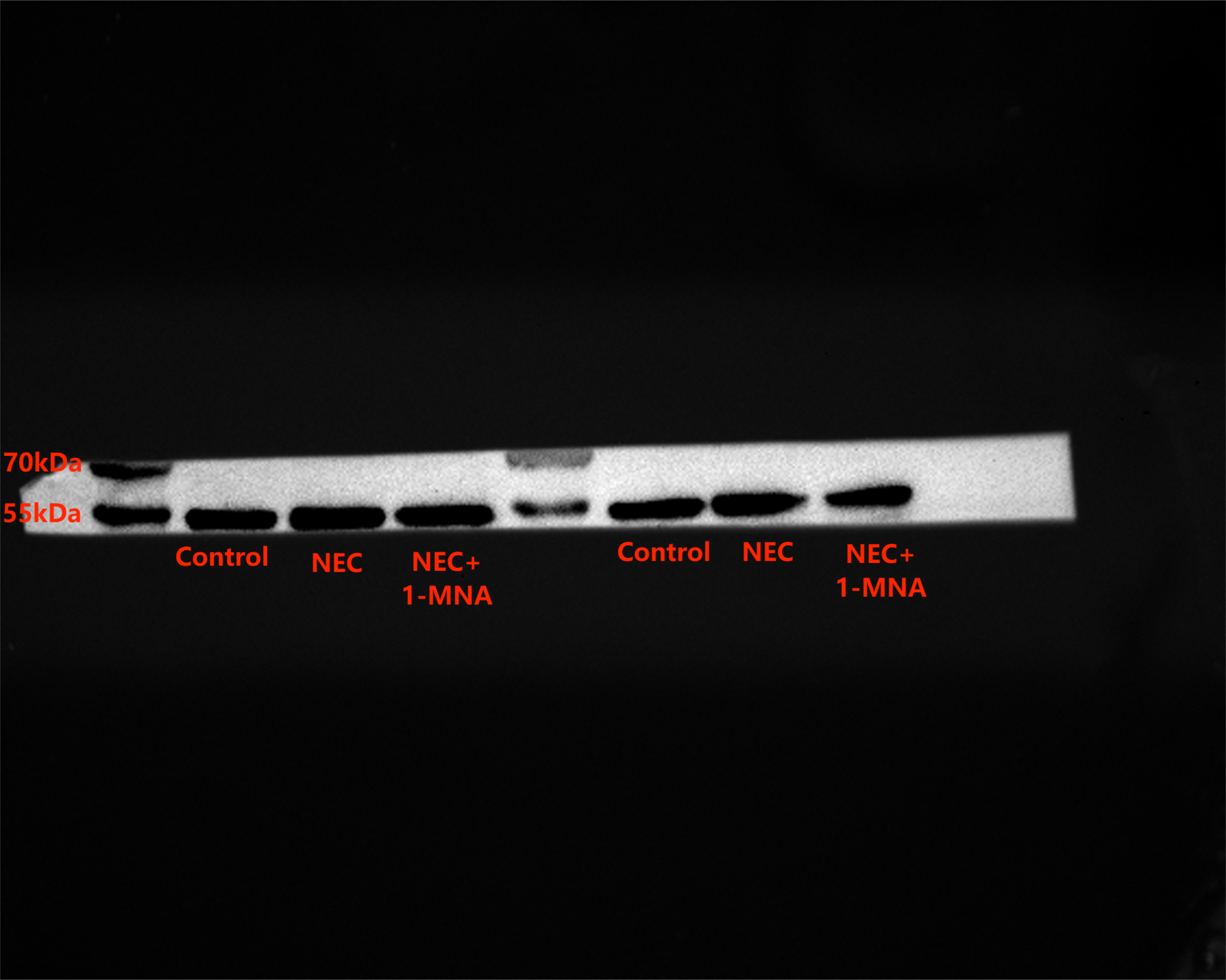

Supplement: S5 File — (ZIP) [file pone.0324068.s005.zip › TLR4(Figure IV)/β-tubulin for TLR4-1.png]

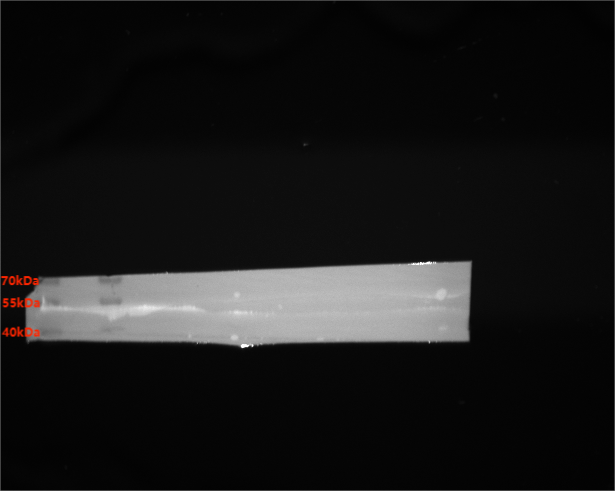

Supplement: S5 File — (ZIP) [file pone.0324068.s005.zip › TLR4(Figure IV)/β-tubulin for TLR4-2 marker.png]

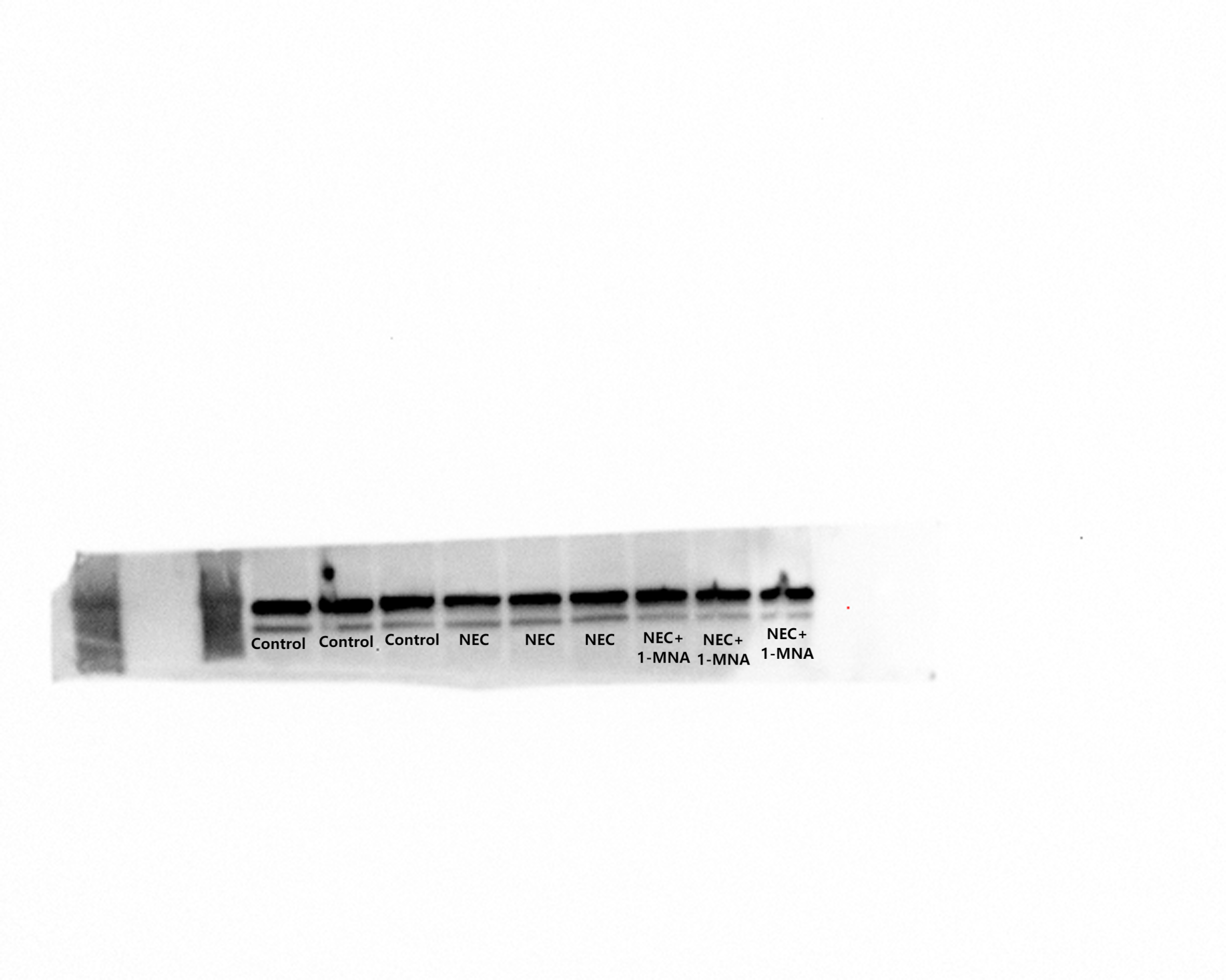

Supplement: S5 File — (ZIP) [file pone.0324068.s005.zip › TLR4(Figure IV)/β-tubulin for TLR4-2.png]

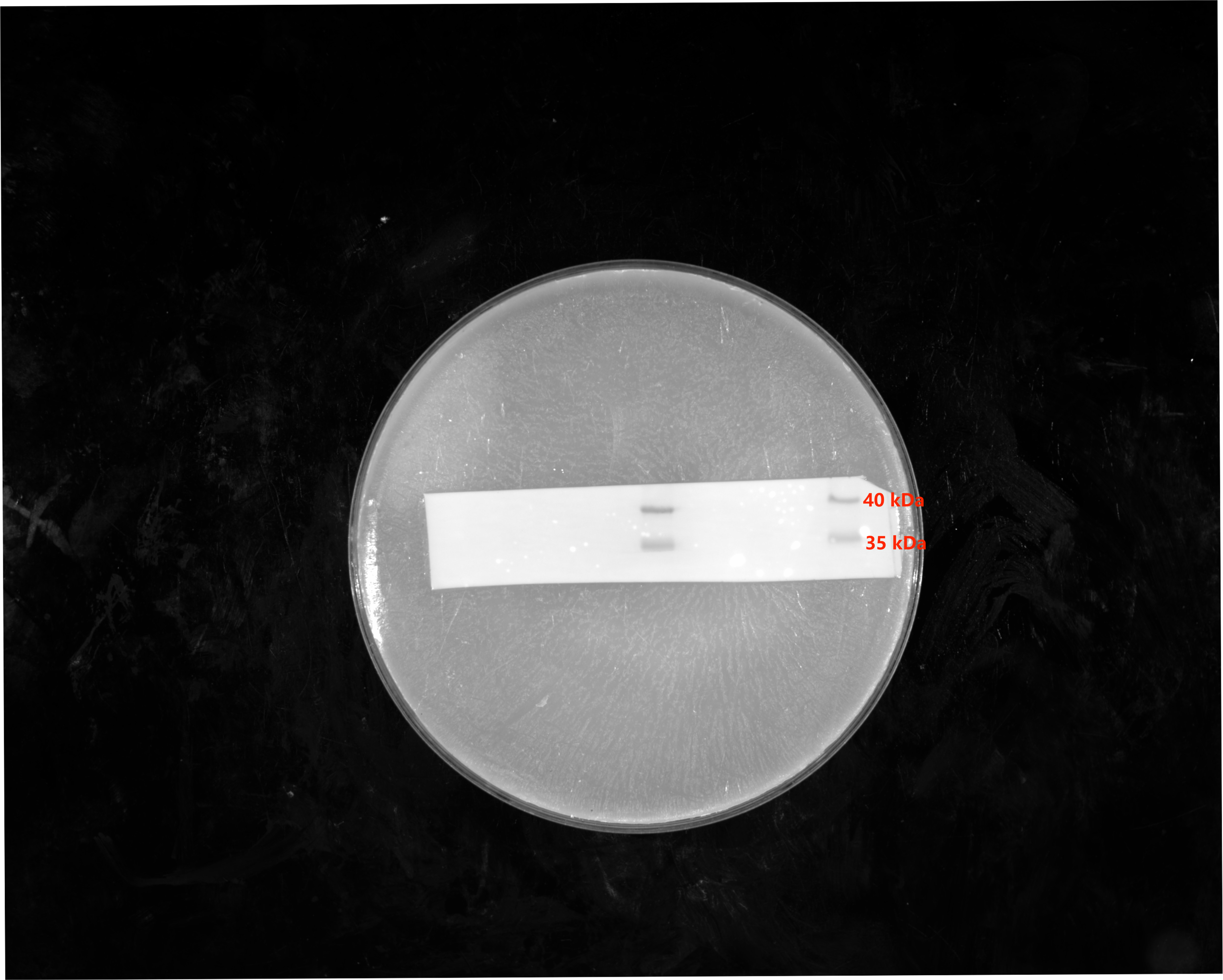

Supplement: S6 File — (ZIP) [file pone.0324068.s006.zip › Phospho-IκBα(Figure IV)/Phospho-IκBα 1 marker.png]

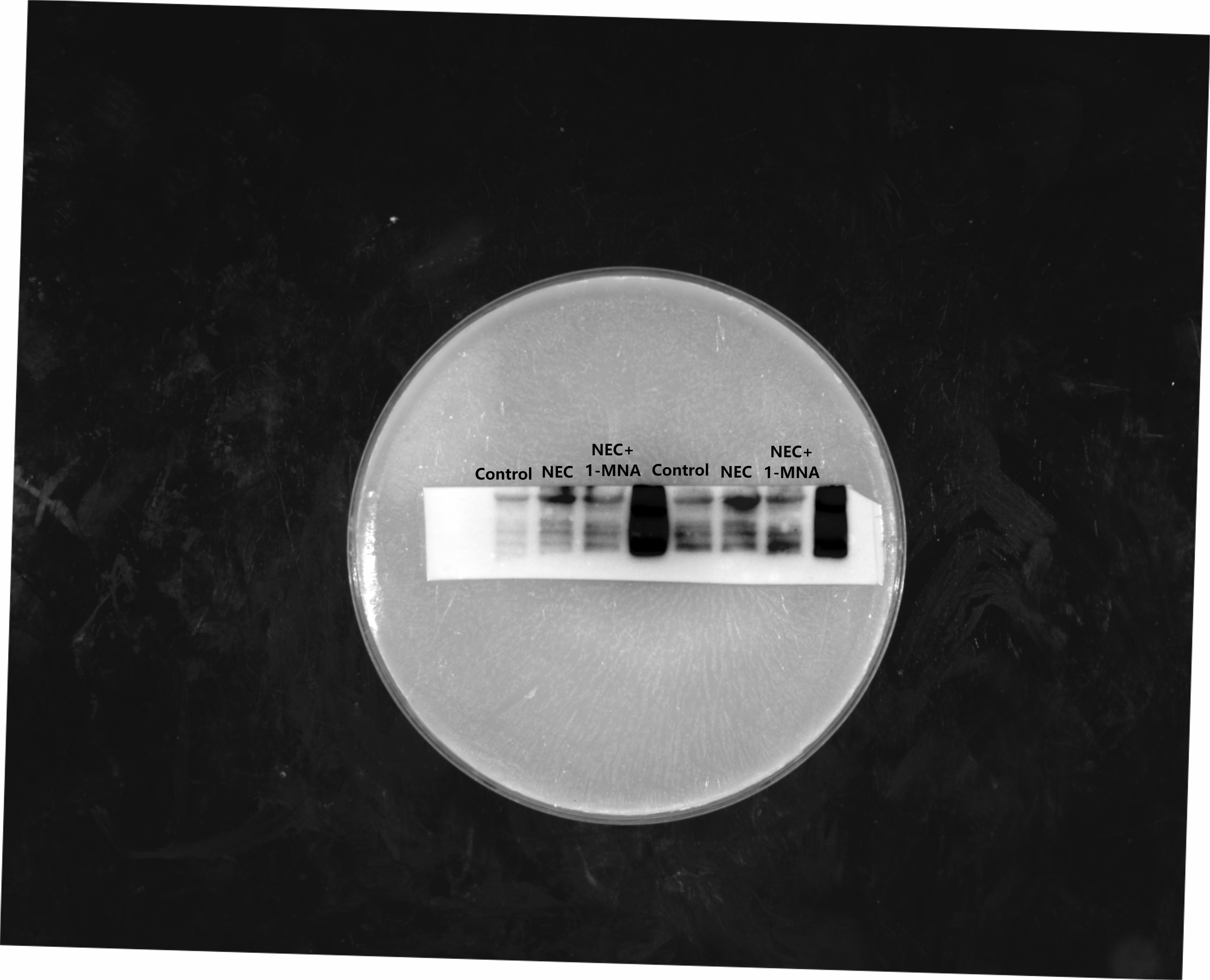

Supplement: S6 File — (ZIP) [file pone.0324068.s006.zip › Phospho-IκBα(Figure IV)/Phospho-IκBα 1.png]

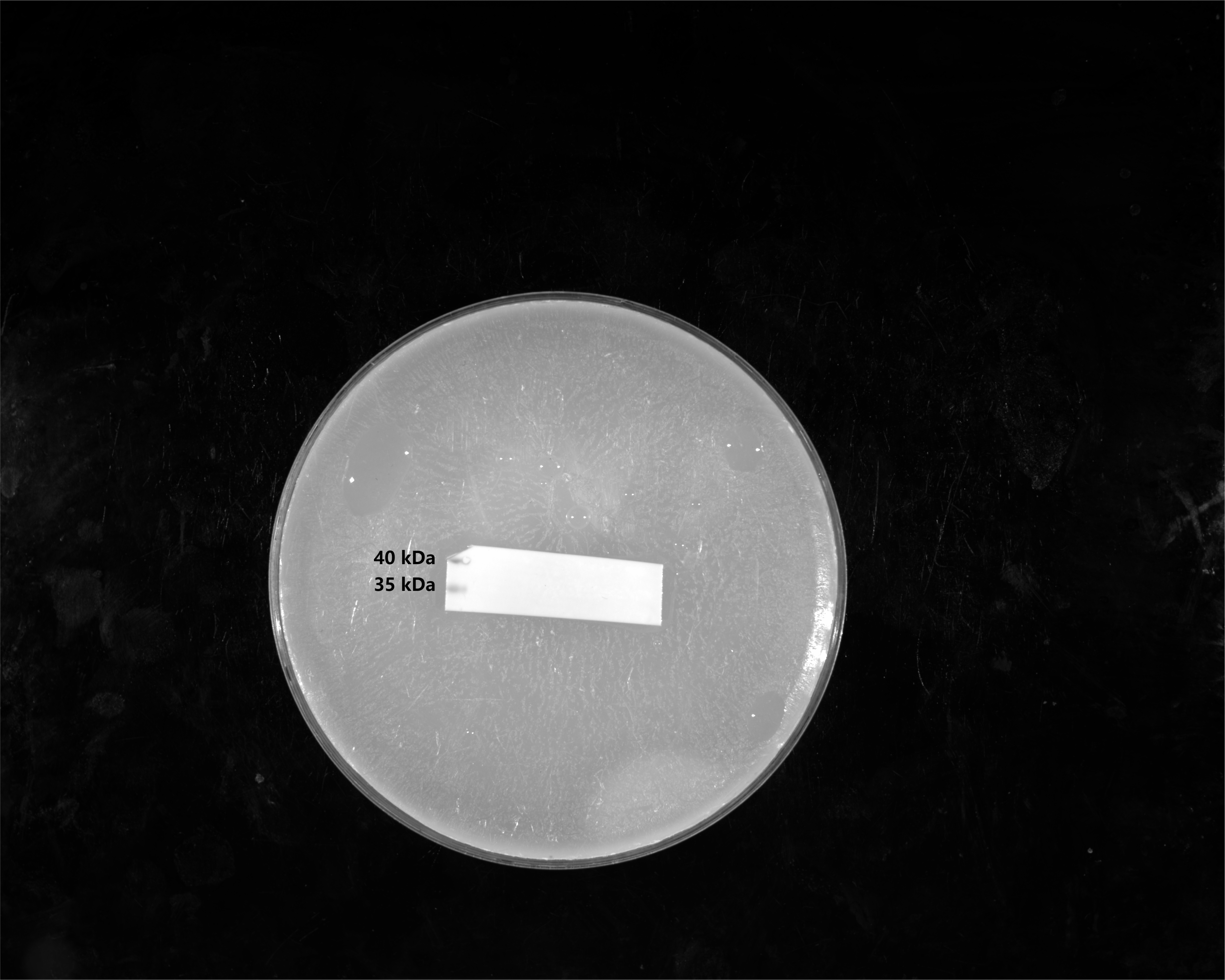

Supplement: S6 File — (ZIP) [file pone.0324068.s006.zip › Phospho-IκBα(Figure IV)/Phospho-IκBα 2 marker.png]

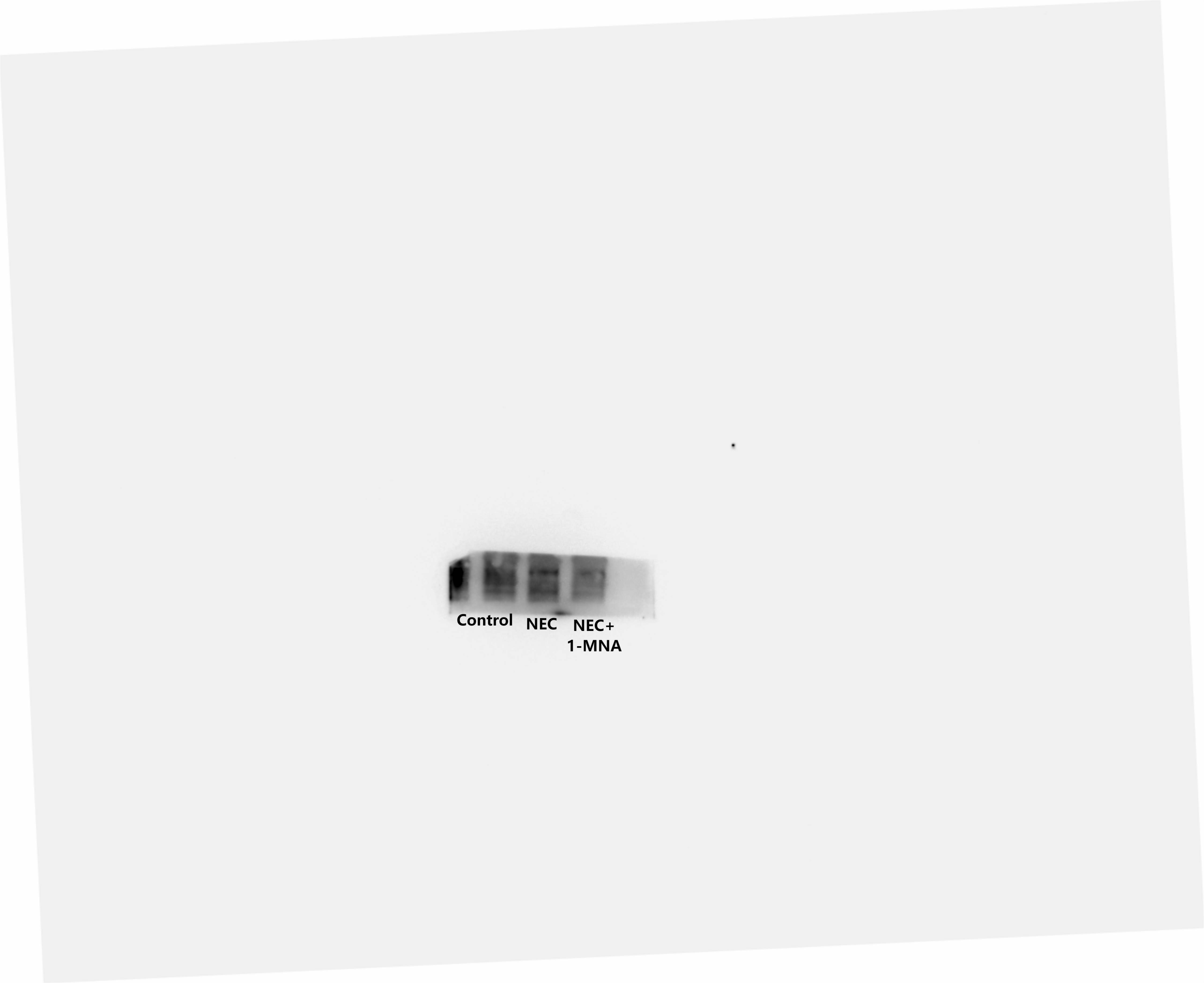

Supplement: S6 File — (ZIP) [file pone.0324068.s006.zip › Phospho-IκBα(Figure IV)/Phospho-IκBα 2.png]

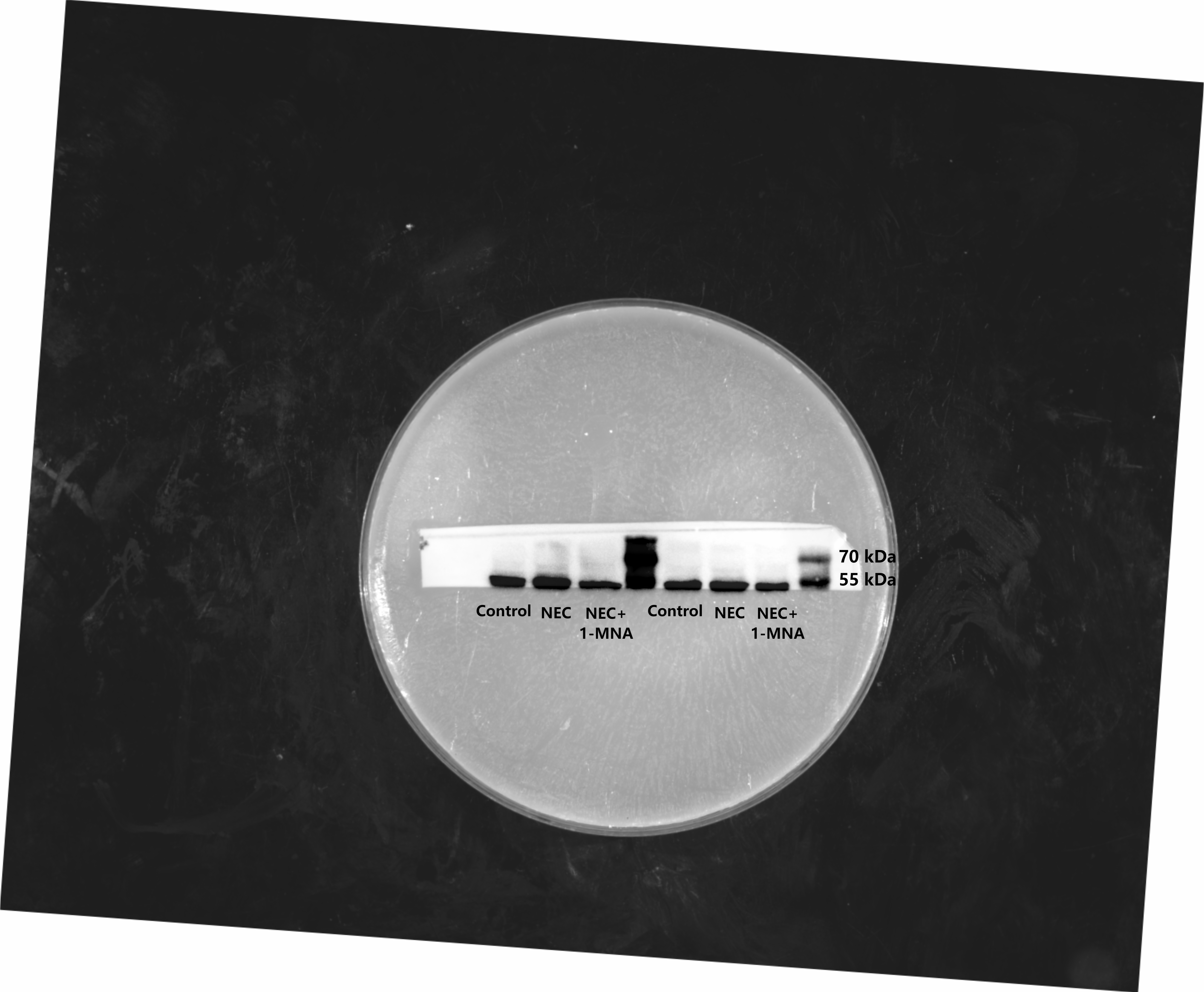

Supplement: S6 File — (ZIP) [file pone.0324068.s006.zip › Phospho-IκBα(Figure IV)/β-tubulin for Phospho-IκBα 1.png]

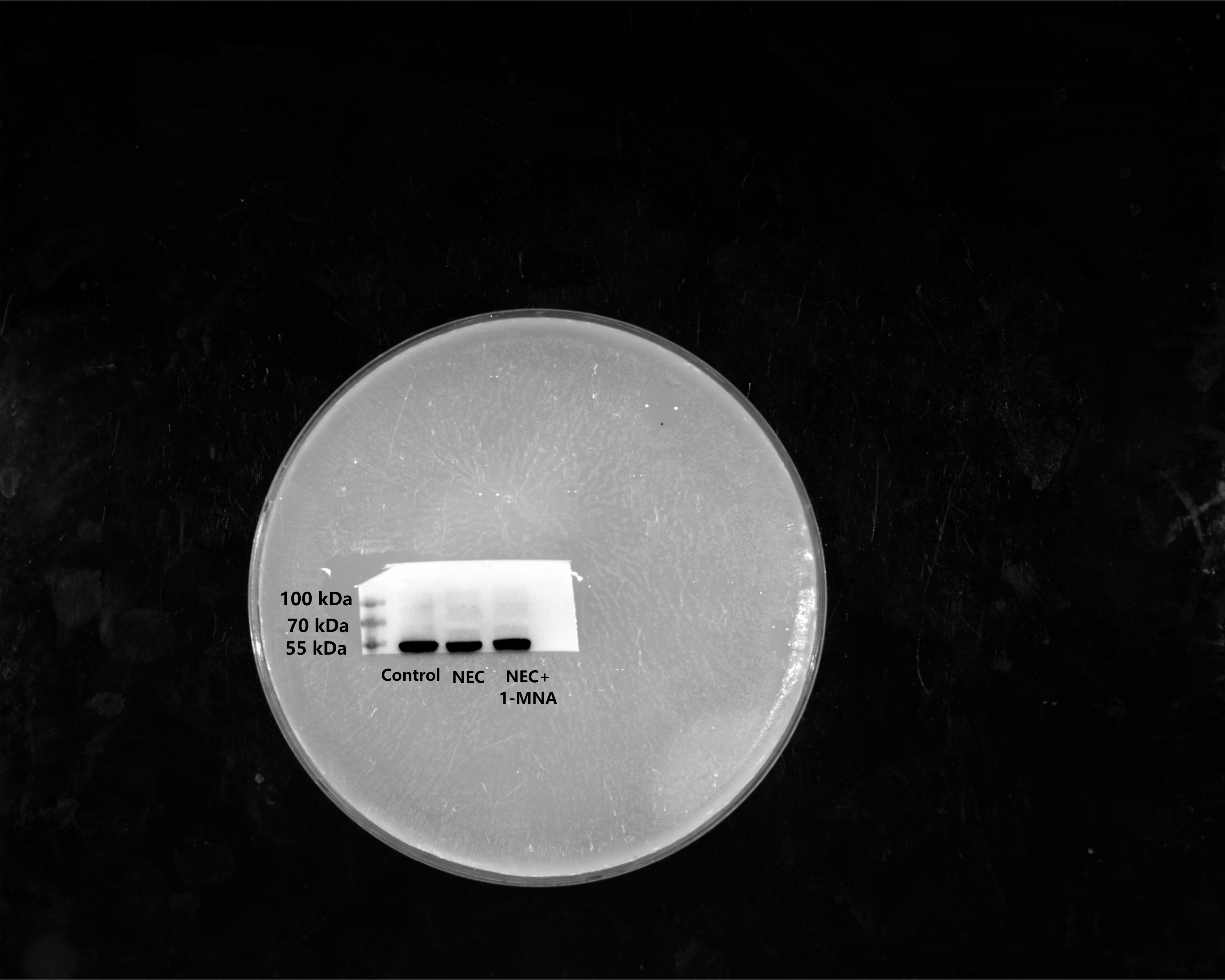

Supplement: S6 File — (ZIP) [file pone.0324068.s006.zip › Phospho-IκBα(Figure IV)/β-tubulin for Phospho-IκBα 2.png]

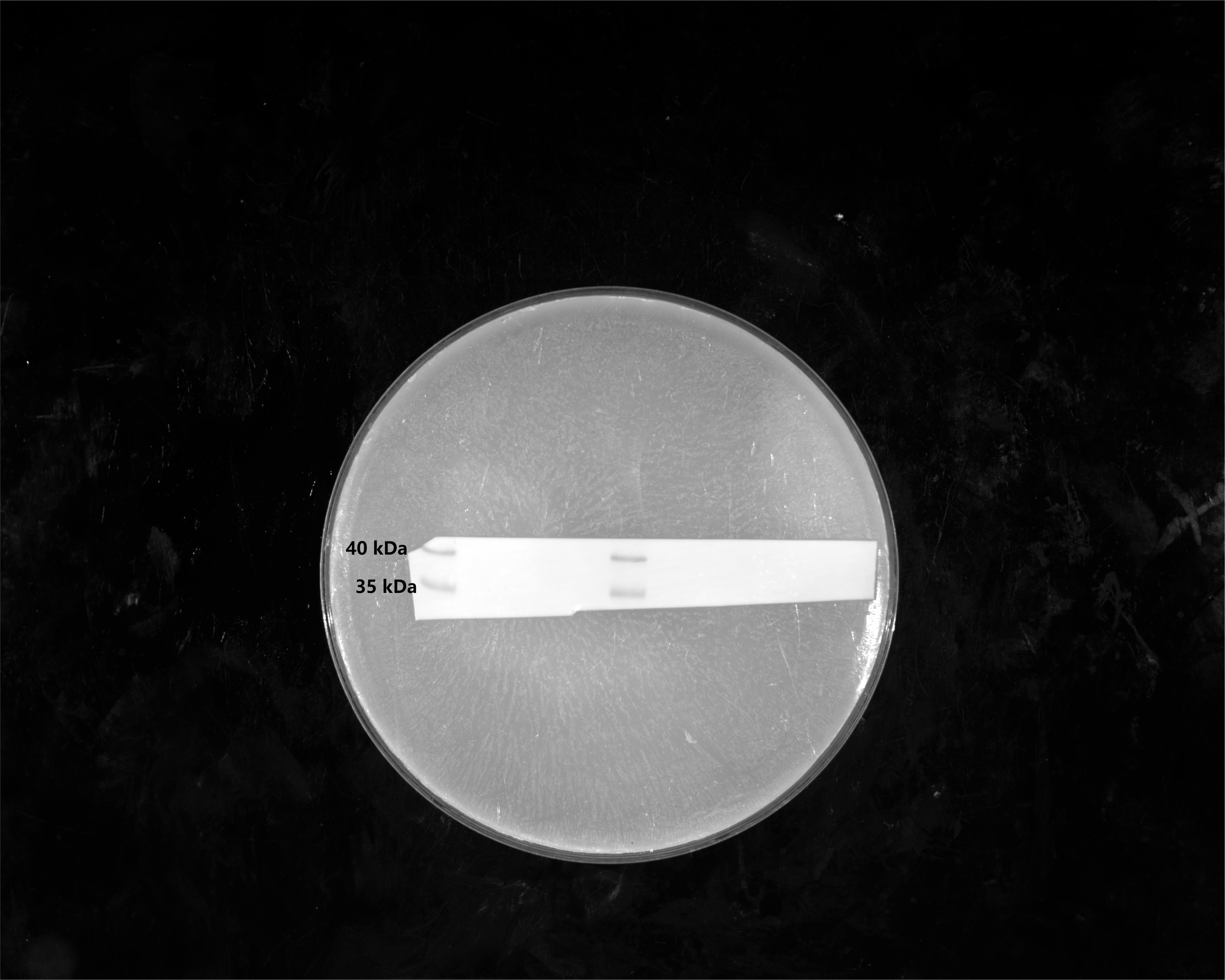

Supplement: S7 File — (ZIP) [file pone.0324068.s007.zip › IκB α(Figure IV)/IκB α 1 marker.png]

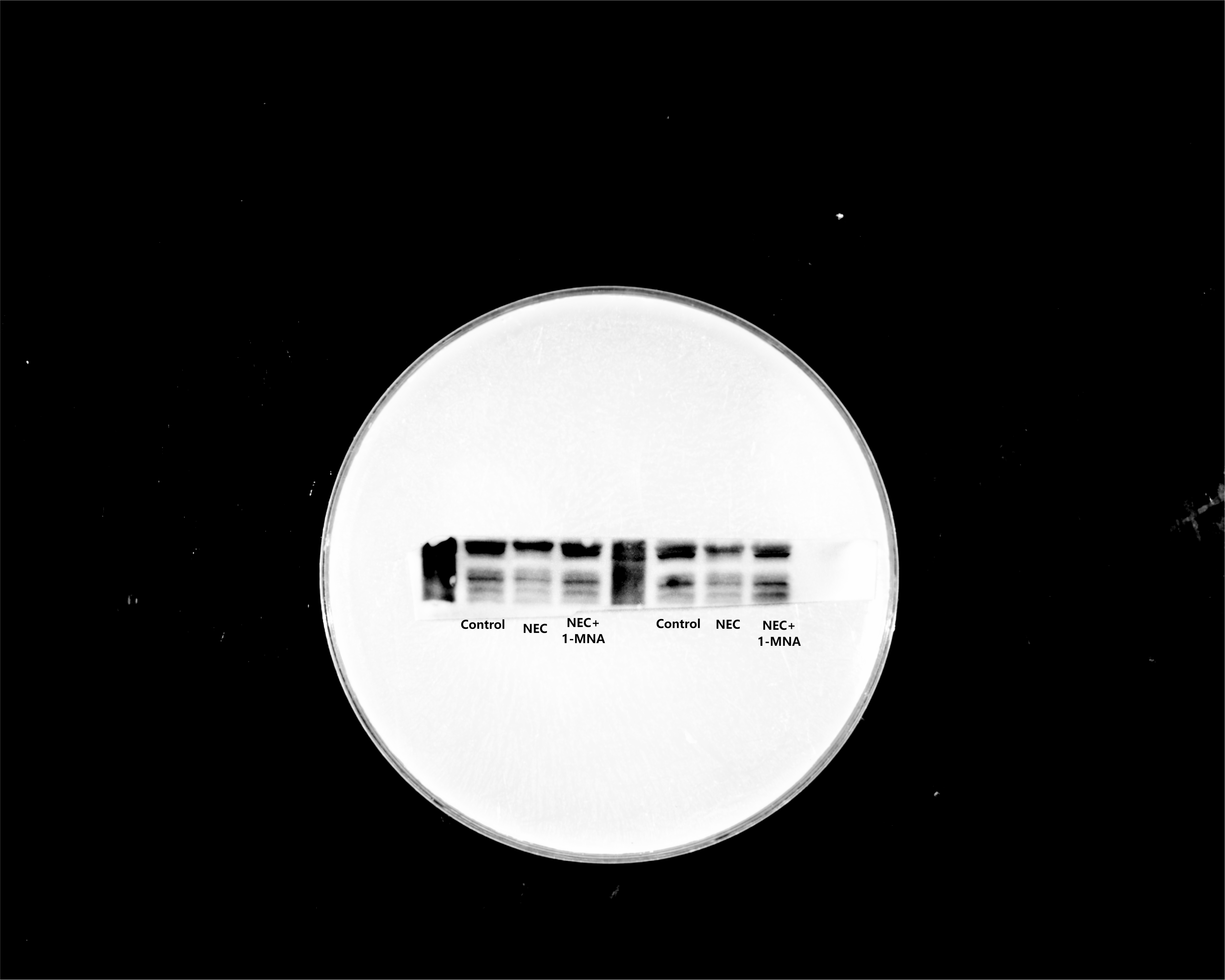

Supplement: S7 File — (ZIP) [file pone.0324068.s007.zip › IκB α(Figure IV)/IκB α 1.png]

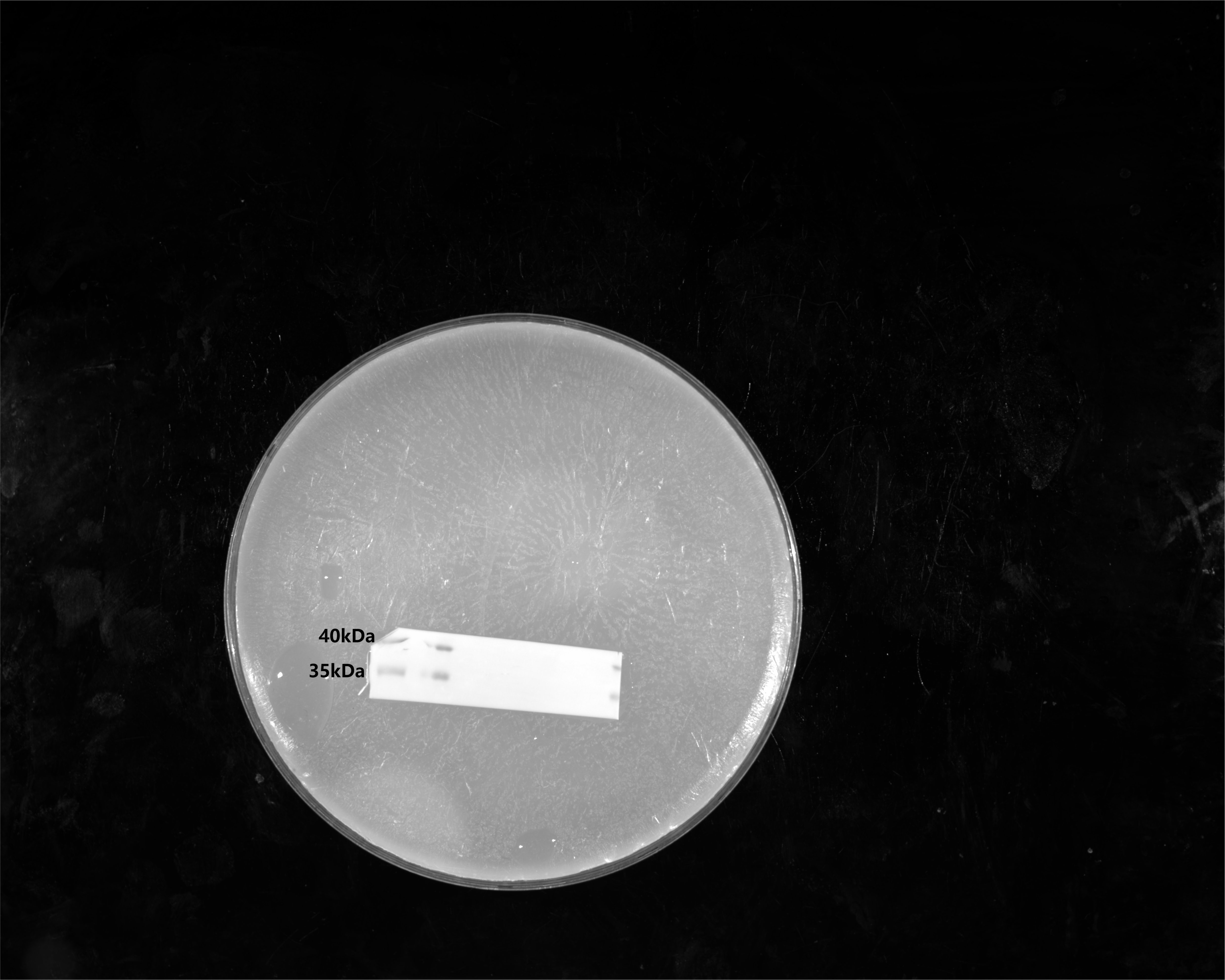

Supplement: S7 File — (ZIP) [file pone.0324068.s007.zip › IκB α(Figure IV)/IκB α 2 marker.png]

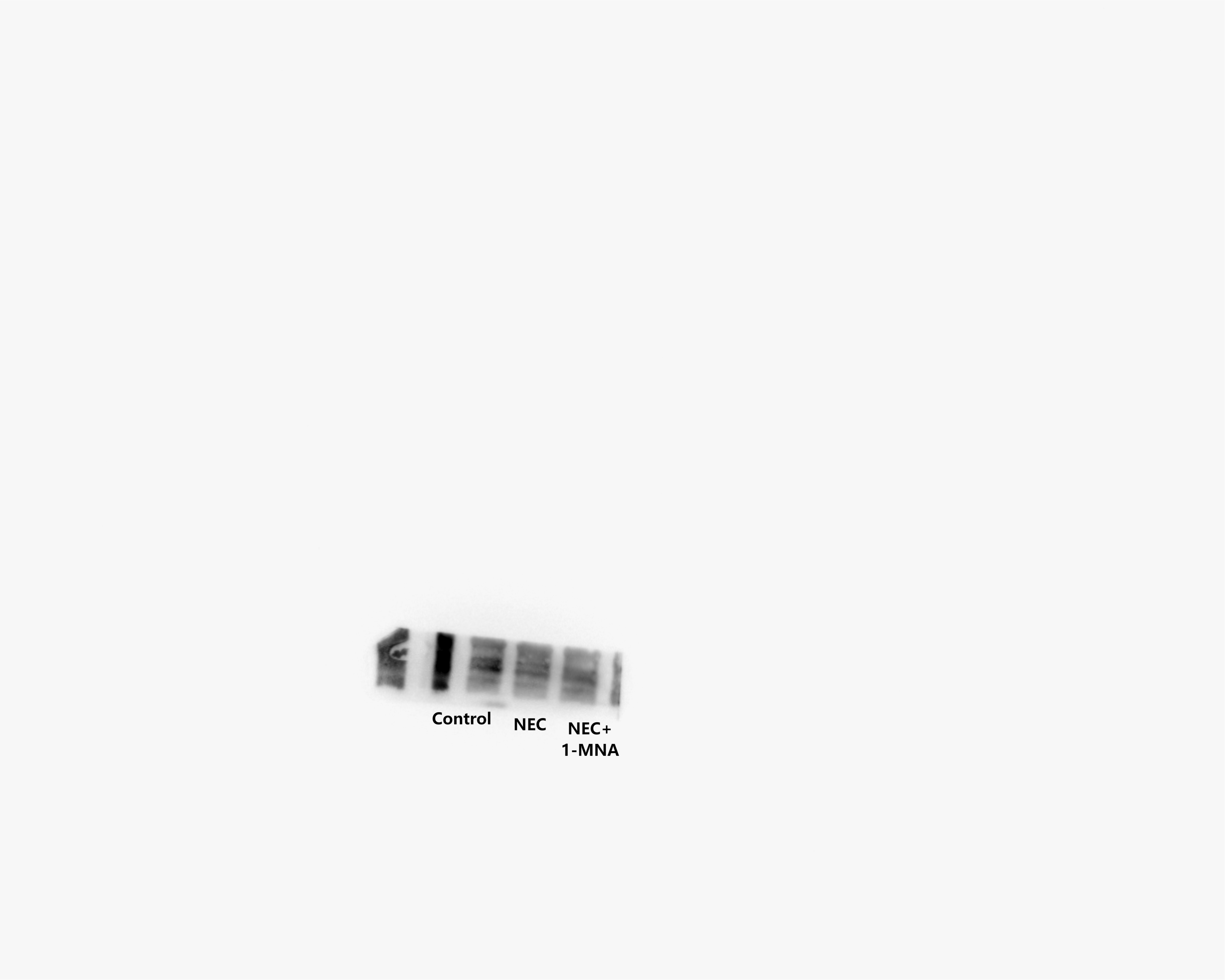

Supplement: S7 File — (ZIP) [file pone.0324068.s007.zip › IκB α(Figure IV)/IκB α 2.png]

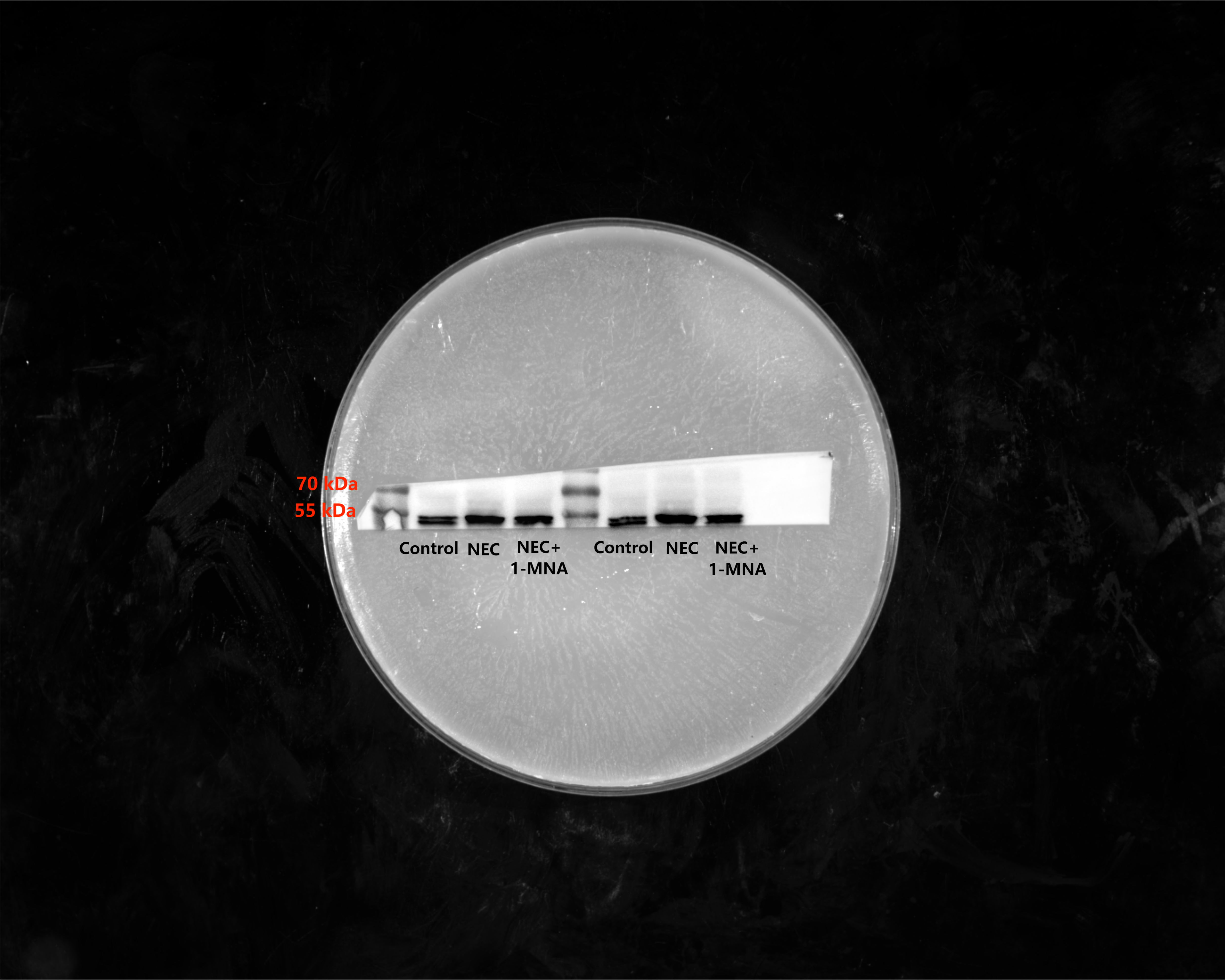

Supplement: S7 File — (ZIP) [file pone.0324068.s007.zip › IκB α(Figure IV)/β-tubulin for IκB α 1.png]

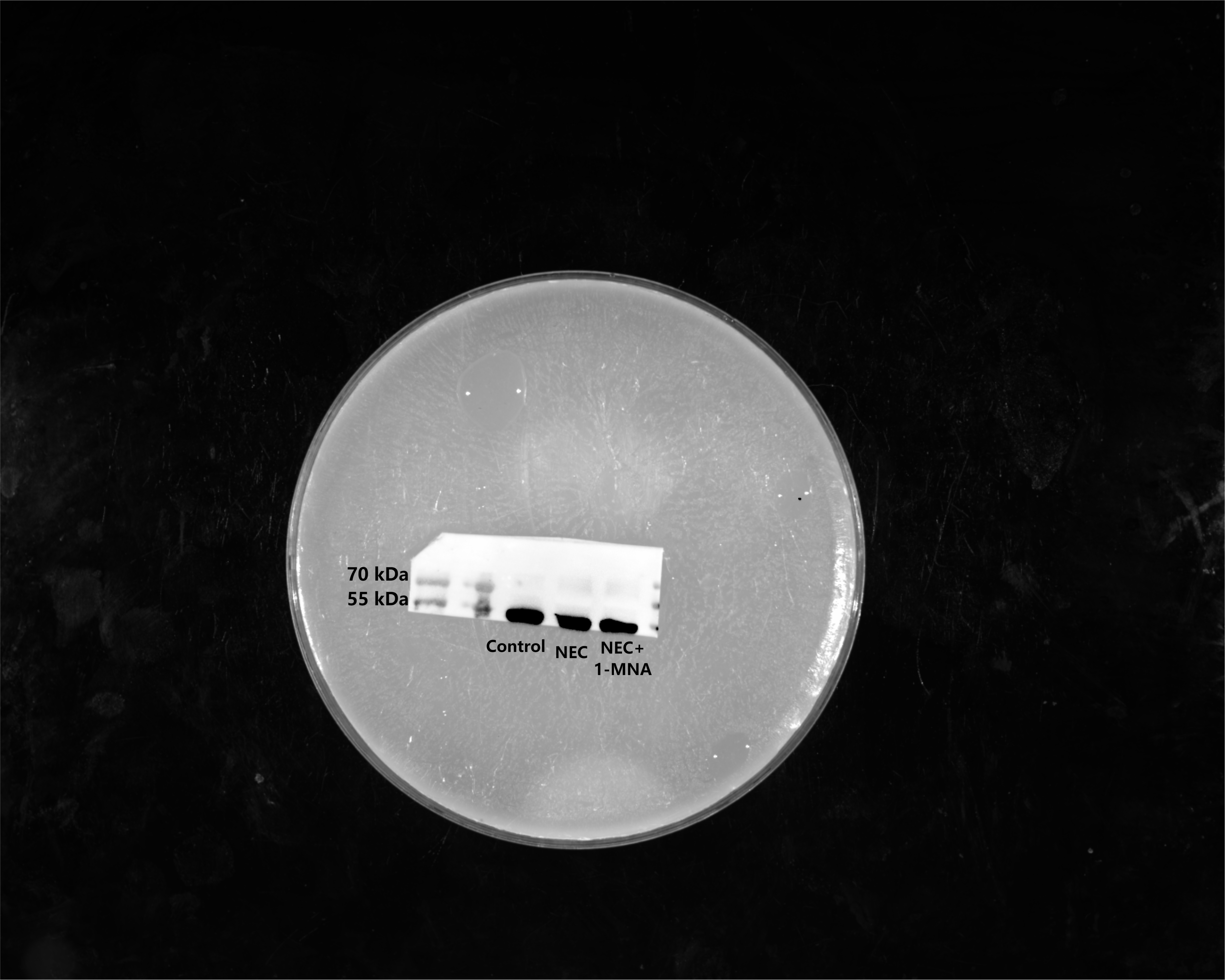

Supplement: S7 File — (ZIP) [file pone.0324068.s007.zip › IκB α(Figure IV)/β-tubulin for IκBα 2.png]
