# Supplementary material for: 1-methylnicotinamide attenuated inflammation and regulated flora in Necrotizing enterocolitis
Source: PLoS One. 2025 Jun 26;20(6):e0324068. doi: 10.1371/journal.pone.0324068 (PMC12200687; doi:10.1371/journal.pone.0324068)
Supplement: Attachment — Submitted filename: Response to Reviewers.docx [file pone.0324068.s009.docx]

Journal Requirements 1：The manuscript has been modified to conform to the templates as much as possible

Journal Requirements 2：western blot of the original image with molecular weight labeling is supplemented in S4-File.

Journal Requirements 3：Details of animal sacrifice have been added to the methods section.

Journal Requirements 4：A complete ethical statement has been added to the methods section.

Reviewer #1 question 1:Reagent codes have been supplemented in the Materials and methods sections.

Reviewer #1 question 2:Representative images for each score of the intestinal histological assessment have been supplemented in the S2-File.

Reviewer #1 question 3:Product code and dilution of antibody have been added in S1 Table.

Reviewer #1 question 4:Molecular weight in all Western imprinted images has been added in S4-File.

Reviewer #1 question 5:Higher magnification images of Figures 2D and 4D-F have been updated.

Reviewer #1 question 6:The negative control of Figure 3C has been added in the S3-File. The higher magnification figure 3C has been updated.

Reviewer #1 question 7:The full name of the first reference to the acronym has been added to the text.

Reviewer #2 question 1:The introduction explains the validity of the study and explains why we decided to focus on NEC pathology.

Reviewer #2 question 2:The product code of antibody and its dilution have been added in S1 Table.

Reviewer #2 question 3:The image resolution has been improved.

Reviewer #2 question 4:16S rRNA genewas targeted have been changed to16S rRNA gene was targeted.
